# Geo-Climatic Changes and Apomixis as Major Drivers of Diversification in the Mediterranean Sea Lavenders (*Limonium* Mill.)

**DOI:** 10.3389/fpls.2020.612258

**Published:** 2021-01-12

**Authors:** Konstantina Koutroumpa, Ben H. Warren, Spyros Theodoridis, Mario Coiro, Maria M. Romeiras, Ares Jiménez, Elena Conti

**Affiliations:** ^1^ Department of Systematic and Evolutionary Botany, University of Zurich, Zurich, Switzerland; ^2^ Institut de Systematique, Evolution, Biodiversité (ISYEB), Muséum National d’Histoire Naturelle, CNRS, Sorbonne Université, EPHE, UA, Paris, France; ^3^ Senckenberg Biodiversity and Climate Research Centre (BiK-F), Senckenberg Gesellschaft für Naturforschung, Frankfurt am Main, Germany; ^4^ Linking Landscape, Environment, Agriculture and Food (LEAF), Instituto Superior de Agronomia (ISA), Universidade de Lisboa, Lisboa, Portugal; ^5^ Centre for Ecology, Evolution and Environmental Changes (cE3c), Faculdade de Ciências, Universidade de Lisboa, Lisboa, Portugal; ^6^ IES Pedra da Auga, Ponteareas, Spain

**Keywords:** Messinian salinity crisis, Mediterranean climate, sea-level fluctuations, asexual reproduction, in situ diversification, island biogeography, Macaronesia, long-distance dispersal

## Abstract

The Mediterranean realm, comprising the Mediterranean and Macaronesian regions, has long been recognized as one of the world’s biodiversity hotspots, owing to its remarkable species richness and endemism. Several hypotheses on biotic and abiotic drivers of species diversification in the region have been often proposed but rarely tested in an explicit phylogenetic framework. Here, we investigate the impact of both species-intrinsic and -extrinsic factors on diversification in the species-rich, cosmopolitan *Limonium*, an angiosperm genus with center of diversity in the Mediterranean. First, we infer and time-calibrate the largest *Limonium* phylogeny to date. We then estimate ancestral ranges and diversification dynamics at both global and regional scales. At the global scale, we test whether the identified shifts in diversification rates are linked to specific geological and/or climatic events in the Mediterranean area and/or asexual reproduction (apomixis). Our results support a late Paleogene origin in the proto-Mediterranean area for *Limonium*, followed by extensive *in situ* diversification in the Mediterranean region during the late Miocene, Pliocene, and Pleistocene. We found significant increases of diversification rates in the “Mediterranean lineage” associated with the Messinian Salinity Crisis, onset of Mediterranean climate, Plio-Pleistocene sea-level fluctuations, and apomixis. Additionally, the Euro-Mediterranean area acted as the major source of species dispersals to the surrounding areas. At the regional scale, we infer the biogeographic origins of insular endemics in the oceanic archipelagos of Macaronesia, and test whether woodiness in the Canarian *Nobiles* clade is a derived trait linked to insular life and a biotic driver of diversification. We find that *Limonium* species diversity on the Canary Islands and Cape Verde archipelagos is the product of multiple colonization events followed by *in situ* diversification, and that woodiness of the Canarian endemics is indeed a derived trait but is not associated with a significant shift to higher diversification rates. Our study expands knowledge on how the interaction between abiotic and biotic drivers shape the uneven distribution of species diversity across taxonomic and geographical scales.

## Introduction

Biodiversity on Earth is unevenly distributed. Species richness varies across habitats, geographic regions, and taxonomic groups, raising long-standing questions about the ecological and evolutionary mechanisms underpinning this variation. Factors that drive speciation and extinction (i.e., species diversification) have altered biodiversity in space and time. Drivers of diversification include abiotic factors, for example, tectonic processes and climatic events (Barnosky, 2001), and biotic factors, for example, reproductive traits, ploidy levels, hybridization, and habit (Soltis et al., 2019). Numerous studies have focused on identifying a single key trait and linking it to shifts in diversification rates (e.g., Mayhew et al., 2008; de Vos et al., 2014; Howard et al., 2020). However, both biotic and abiotic factors can act synergistically toward changes in diversification (e.g., Bouchenak-Khelladi et al., 2015; Donoghue and Sanderson, 2015; Condamine et al., 2018). Analyses of spatio-temporal evolution and drivers of diversification in species-rich lineages are crucial to clarify the role of past biotic and abiotic changes in the origins of species diversity and predict how lineages will be affected by ongoing environmental changes.

Flowering plant diversity is partitioned taxonomically, geographically, and environmentally. Angiosperms comprise more than 13,000 genera (Christenhusz and Byng, 2016) ranging in size from a single to thousands of species, yet only about 70 genera are characterized as species rich (with ≥500 species; Frodin, 2004; Mabberley, 2017). Furthermore, plant diversity is concentrated in 36 global biodiversity hotspots that cover only 16% of Earth’s surface but harbor more than 50% of endemic vascular plants and are undergoing remarkable loss of habitat (Myers et al., 2000; Mittermeier et al., 2011). The Mediterranean has been identified as one of the world’s biodiversity hotspots by Mittermeier et al. (2011) and Critical Ecosystem Partnership Fund (2019). These authors defined the Mediterranean hotspot as comprising the Mediterranean region (i.e., mainland areas surrounding the Mediterranean Sea and the islands in it) and Macaronesia (i.e., Canaries, Cape Verde, Azores, Madeira, and Selvagen Islands). The Mediterranean region and Macaronesia combined are also often referred to as the Mediterranean realm (e.g., Comes, 2004; Mansion et al., 2009). This hotspot covers only 1.6% of the Earth’s surface, yet accommodates 10% of its total plant species richness, representing the third richest hotspot with approximately 25,000 species, more than half of which are endemic (Medail and Quezel, 1997; Blondel et al., 2010; Critical Ecosystem Partnership Fund, 2019). Its geographic location at the crossroads of three continents (Europe, Africa, and Asia) makes the Mediterranean a large contact zone for taxa of different biogeographic origins (e.g., Eurasian Circumboreal, Irano-Turanian, and Saharo-Arabian), which, together with taxa that originated and diversified *in situ*, form its remarkably diverse flora (Blondel and Aronson, 1999).

The Mediterranean region has undergone multiple geological and climatic upheavals (Thompson, 2005). Geologically, the region originated from two ancient, independent ocean basins: the Alpine Tethys Ocean (opened during the Middle to Late Jurassic and related to the opening of the Central Atlantic) in the Northwest and the Neotethys Ocean (opened from the Triassic to the Jurassic between Laurasia and Gondwana) in the Southeast (van Hinsbergen et al., 2020 and references therein). From the Cretaceous to the early Miocene, a continuing convergence of tectonic plates brought Europe and Africa progressively closer (Rosenbaum et al., 2002). In the late Miocene, uplift at the continental margins of Iberia and Africa triggered extensive basin desiccation (Duggen et al., 2003; Gargani and Rigollet, 2007). This period, known as the Messinian Salinity Crisis (MSC, from ca. 5.96 to 5.33 Ma; Krijgsman et al., 1999), has been described as “one of the most dramatic events on Earth during the Cenozoic era” (Hsü et al., 1973; Duggen et al., 2003).

The MSC and the onset of the Mediterranean climate (3.2–2.8 Ma; Suc, 1984) were landmark events in the evolution of diversity in the Mediterranean region (Fiz-Palacios and Valcárcel, 2013). The creation of saline deserts during the MSC (Hsü et al., 1973) produced land bridges between islands and continental areas that potentially facilitated migrations of plants with the necessary dispersal properties and salt-tolerance (e.g., halophytes). The MSC is considered to have facilitated speciation in arid-adapted lineages and extinction in sub-tropical Tertiary lineages (Rodríguez-Sánchez et al., 2008; Jiménez-Moreno et al., 2010; Crowl et al., 2015). The refilling of the basin at the end of the MSC disrupted previously formed land bridges, thus promoting vicariance, and mitigated aridity (García-Castellanos et al., 2009), thus possibly causing extinction of arid-adapted lineages (Fiz-Palacios and Valcárcel, 2013). While the effects of MSC on the Mediterranean flora are still debated, the positive effects of the emergence of the Mediterranean climatic regime on diversification are corroborated by multiple studies (e.g., Valente et al., 2010; Fiz-Palacios and Valcárcel, 2011). Furthermore, several plant lineages show a temporal period of reduced diversification rate from the Messinian event to the onset of the Mediterranean climate that has been variably attributed to either mass extinction, rate stasis, or a combination of the two (Fiz-Palacios and Valcárcel, 2013).

During the late Pliocene-early Pleistocene, cooler and dryer conditions were implicated in several extinctions (Bessedik et al., 1984), while Pleistocene glacial cycles and eustatic sea-level changes (2.58–0.01 Ma; Lisiecki and Raymo, 2007) further impacted Mediterranean plant diversification and distributions. Pleistocene geoclimatic oscillations caused species range contractions and expansions as plant populations fragmented and merged in response to the appearance and disappearance of dispersal barriers through time, with contrasting effects on diversification of different Mediterranean plant lineages (Nieto Feliner, 2014). Furthermore, different types of islands (oceanic and continental), substrates, and microclimates provided opportunities for adaptation and speciation driven by both ecological and geographical barriers. Overall, geomorphological and climatic processes, coupled with a long history of human activities in the Mediterranean, created a mosaic of heterogeneous habitats, where a diversity of abiotic factors had profound impacts on diversification.

In addition to extrinsic environmental factors, inherent biological features also play a key role in diversification. For example, hybridization, polyploidy, plant habit, and reproductive strategies have all been invoked to explain species divergence and eco-geographical differentiation in plants (Rieseberg et al., 2003; Goldberg et al., 2010; Goldberg and Igić, 2012; Soltis et al., 2013). In particular, asexual reproduction *via* apomixis (i.e., cloning through seeds; Asker and Jerling, 1992) can promote rapid diversification by enabling reproductively isolated genotypes to form rapidly, providing reproductive assurance in the absence of pollinators and/or mates, and offering an escape from sterility in newly formed polyploid hybrid individuals (Baker, 1955; Darlington, 1958; Majeský et al., 2017). Apomixis can enable the establishment of a new population from a single individual, an analogous effect to that of selfing as proposed by Baker (1955). Furthermore, in oceanic island systems, such as Macaronesia, the evolution of woodiness from herbaceous ancestors following island colonization has been proposed as a key driver of insular radiations (Nürk et al., 2019). Thus, elucidating the timing, space, and rates of diversification of Mediterranean and Macaronesian lineages and attempting to correlate them with likely biotic and abiotic drivers is essential to explain the evolution of biodiversity in the Mediterranean hotspot (Comes, 2004).

To achieve a deeper understanding of diversification dynamics in the Mediterranean realm, it is necessary to focus on widely distributed plant groups with high species diversity in this region and biotic traits that could act as triggers of diversification given the unique geo-climatic history of the area. *Limonium* Mill. (sea lavender; Plumbaginaceae) qualifies as such a group. It is a species-rich genus (ca. 600 species; Koutroumpa et al., 2018) in the top 0.005% of angiosperm genera for size (according to Frodin’s, 2004 criteria) and has a cosmopolitan distribution with center of diversity in the Mediterranean (ca. 70% of the species, mostly endemics, occur in the region). *Limonium* species, characterized as facultative halophytes (i.e., salt tolerant), grow predominantly on saline and metal-rich soils of mainland and coastal areas (Erben, 1978). They represent an important component of coastal vegetation in Mediterranean ecosystems and contribute the dominant species in several vegetation types (e.g., *Crithmo-Limonietea* class; Brullo et al., 2017). *Limonium* displays marked variation of chromosome numbers, ranging from 12 to 18 in diploids and from 24 to 72 in polyploids (Erben, 1979; Brullo and Erben, 2016). Sea lavenders can reproduce both sexually and asexually *via* apomixis, with most sexual species characterized by pollen-stigma dimorphism and sporophytic self-incompatibility (Baker, 1966). Apomixis occurs exclusively in polyploid taxa, some of presumed hybrid origin (Erben, 1979). The combined effects of polyploidy, hybridization, and apomixis have been suggested as having shaped *Limonium* diversity in the Mediterranean region (e.g., Ingrouille, 1984; Palacios et al., 2000; Lledó et al., 2005). Previous phylogenetic and taxonomic analyses concluded that all described apomictic species of *Limonium*, together with some sexual species, belong to a single large clade formed by the vast majority of Mediterranean endemics, named the “Mediterranean lineage” by Koutroumpa et al. (2018). The same study placed the Macaronesian endemics in four clades of the *Limonium* tree, with the majority of them included in the *Nobiles* and *Jovibarba-Ctenostachys* clades. The *Nobiles* clade consists entirely of Canarian endemics with a woody (suffruticose) habit. The *Jovibarba-Ctenostachys* clade comprises endemics of the Canaries and Cape Verde archipelagos together with endemics in Morocco and Hispaniola (Malekmohammadi et al., 2017; Koutroumpa et al., 2018). A previous phylogenetic study, limited to ca. 8% of *Limonium* species and based on a single biogeographic calibration, inferred a late Miocene (ca. 6–7 Ma) origin for the Mediterranean clade of *Limonium* with most diversification during the Messinian and Pliocene (Lledó et al., 2005). The same study inferred the diversification of Macaronesian clades between the late Miocene and Pliocene. Owing to its large size, worldwide distribution, and uneven diversity among regions, *Limonium* is an ideal group to elucidate the factors shaping the partitioning of biodiversity through space and time, warranting a new study with enhanced sampling and methodology.

Here, we generate and time calibrate the largest phylogeny to date of *Limonium* and Plumbaginaceae. By reconstructing ancestral areas of distribution, inferring the tempo of diversification, and estimating diversification rate dynamics, we address the following questions. At the global scale we ask: (1) When and where did the genus originate and diversify? (2) Can we detect significant shifts of diversification rates and link them to specific abiotic (major geological and/or climatic events) and/or biotic factors (apomixis)? More specifically, we test whether diversification rates are constant or heterogeneous in *Limonium*, and whether potential rate changes are concomitant with changes in extrinsic and intrinsic variables. At the regional scale, we focus on island biogeography, trait evolution, and diversification of Macaronesian *Limonium* by asking: (1) What are the biogeographic origins of island endemics in Macaronesia? (2) Did the transition from herbaceousness to woodiness precede or follow island colonization in the Canarian *Nobiles* clade? Specifically, we hypothesize that woodiness is a derived state linked to insular life, as suggested by several studies (e.g., Carine et al., 2010; Lens et al., 2013), rather than an ancestral state preserved in islands, as proposed by the “islands-as-museums” hypothesis (Cronk, 1997). (3) Did the transition to woodiness trigger an increase in diversification rate in the Canarian *Nobiles* clade, as found in other insular clades (Nürk et al., 2019)? Our findings highlight the importance of both extrinsic and intrinsic factors in generating the remarkable plant diversity of a global biodiversity hotspot, the Mediterranean realm.

## Materials and Methods

### Taxon and Molecular Sampling

We sampled more than one-third of all *Limonium* species (i.e., 216 taxa representing 214 species: 212 species were identified at the species level, one species was identified at the subspecies level, and one species was represented by three varieties) together with 66 species of 20 other Plumbaginaceae genera. Additionally, 20 species from the sister family of Polygonaceae (both subfamilies sampled) were used as outgroup taxa for phylogenetic inference (Supplementary Table S1). Our sampling of *Limonium* includes representatives from all taxonomic subdivisions and phylogenetic clades (Malekmohammadi et al., 2017; Koutroumpa et al., 2018) and spans its cosmopolitan distribution. Sampling of Plumbaginaceae and Polygonaceae provided appropriate nodes for fossil and secondary calibrations. Overall, our molecular dataset comprised 302 taxa and a total of 1,039 sequences from one nuclear (261 ITS sequences) and three chloroplast (291 *trnL-F*, 243 *rbcL*, and 244 *matK* sequences) markers. Here, we increase taxon sampling by 15 taxa compared to the previously published phylogeny of *Limonium* and Plumbaginaceae of Koutroumpa et al. (2018). Molecular methods for 70 newly generated sequences and alignments followed Koutroumpa et al. (2018).

### Phylogenetic and Dating Analyses

Phylogenies were estimated using maximum likelihood (ML) and Bayesian inference (BI) as implemented in RAxML 8.2.10 (Stamatakis, 2014) and MrBayes 3.2.6 (Ronquist et al., 2012), respectively, following conditions described in Koutroumpa et al. (2018; except for BI of supermatrices for which chains ran for 30 million generations). Trees were inferred for each locus separately and in concatenation (concatenated datasets: 3-loci cpDNA matrix and 4-loci “Supermatrix”). We inspected the resulting cpDNA and ITS trees to detect incongruences between well-supported clades (as defined in Koutroumpa et al., 2018) and identify “rogue taxa” (i.e., taxa placed in different well-supported clades in the cpDNA vs. the ITS tree). Given the small number of conflicts thus identified, we additionally inferred phylogenies for a Supermatrix from which we excluded ITS sequences of “rogue clades and taxa” (hereafter “Supermatrix-cpDNA-like”) and a Supermatrix from which we excluded cpDNA sequences of “rogue clades and taxa” (hereafter “Supermatrix-ITS-like”). Trees from these two trimmed supermatrices have higher resolution than those from the three-loci cpDNA matrix and ITS matrix.

Divergence time estimates were performed in BEAST 2.5.1 (Bouckaert et al., 2019) for ITS, cpDNA, “Supermatrix,” “Supermatrix-ITS-like,” and “Supermatrix-cpDNA-like” datasets, using five calibration points. Following a comprehensive review of literature for the Plumbaginaceae-Polygonaceae fossil record and dated angiosperm phylogenies, and following guidelines for nodal assignment of fossils (Magallon and Sanderson, 2001; Rutschmann et al., 2007), we calibrated four nodes (two within Plumbaginaceae and two within Polygonaceae), and the stem node of Plumbaginaceae, forming a total of five calibration points (see Supplementary Figures S1, S2); the details of the calibration process are described below. Although fossils for Plumbaginaceae are sparse, two internal nodes could be calibrated: an *Armeria*-type pollen fossil from upper Miocene (Van Campo, 1976; Muller, 1981) was used as a minimum age constraint (5.333 Ma) for the Limonieae crown-node (the origin of *Armeria*-type pollen according to Costa et al.’s, 2019 ancestral state estimates), and a pollen fossil of *Ceratolimon* cf. *feei* (former *Limoniastrum*; Beucher, 1975; Muller, 1981) from the Pliocene was used as minimum age constraint (2.58 Ma) for the *Ceratolimon* stem-node. We also used two fossils from Polygonaceae: a *Coccoloba* pollen fossil from the upper Miocene (Graham, 1976; Muller, 1981) was used to assign a minimum age (5.333 Ma) to the *Coccoloba* stem-node and *Muehlenbeckia*-type pollen (†*Rhoipites muehlenbeckiaformis*; Macphail, 1999) from the upper Eocene was used as minimum age constraint (33.9 Ma) for the *Muehlenbeckia* stem-node. Finally, we used a secondary calibration for the stem of Plumbaginaceae based on age estimates from the dated angiosperm phylogeny of Magallón et al. (2015) [mean: 67.9 Ma and 95% Highest Posterior Density (HPD): 65.63–78.21 Ma]. We employed uniform priors for fossil calibrations with a hard maximum bound of 78.21 Ma (Magallón et al.’s, 2015 HPD max age for Plumbaginaceae-Polygonaceae split) and minimum bounds mentioned above. Employing the conservative approach of uniform priors, we consider that fossils can provide only minimum ages and that paucity of fossil records hinders the use of more informative priors (similar to other angiosperm studies, e.g., Bouchenak-Khelladi et al., 2014; Boucher et al., 2016). For the secondary calibration, we ran analyses using either a normal (mean age of 67.9 Ma and SD of 5.25 Myr) or a uniform prior (65.63–78.21 Ma), which produced very similar age estimates (Supplementary Table S2). Thus, we present results from analyses with uniform priors following Schenk (2016), who demonstrated reduced error in uniform vs. normal priors for secondary calibrations.

Divergence times were inferred using a relaxed uncorrelated lognormal clock and a nucleotide substitution model-averaging method (bModelTest tool; Bouckaert and Drummond, 2017) for each of the two partitions (ITS vs. cpDNA). bModelTest integrates over 203 time-reversible models while simultaneously estimating other parameters (estimates weighted by the support of each model). Independent runs of 200 million generations were combined (after removing up to 22.5% as burn-in) using LogCombiner 2.5.1, and maximum clade credibility (MCC) trees were constructed in TreeAnnotator 2.5.1 (Drummond et al., 2012). Chain convergence was verified in Tracer 1.7.1 [Effective Sample Size (ESS) >200 for all parameters; Rambaut et al., 2018]. The 95% HPD intervals of inferred ages for the five datasets overlapped with each other (Supplementary Table S2). Thus, we performed all subsequent analyses on the *Limonium* clade pruned from the “Supermatrix-ITS-like” and “Supermatrix-cpDNA-like” MCC trees (see also justification above), unless otherwise specified. BEAST and MrBayes analyses were performed using the CIPRES Science Gateway (Miller et al., 2010).

### Biogeographic Analyses

**Figure 1:**
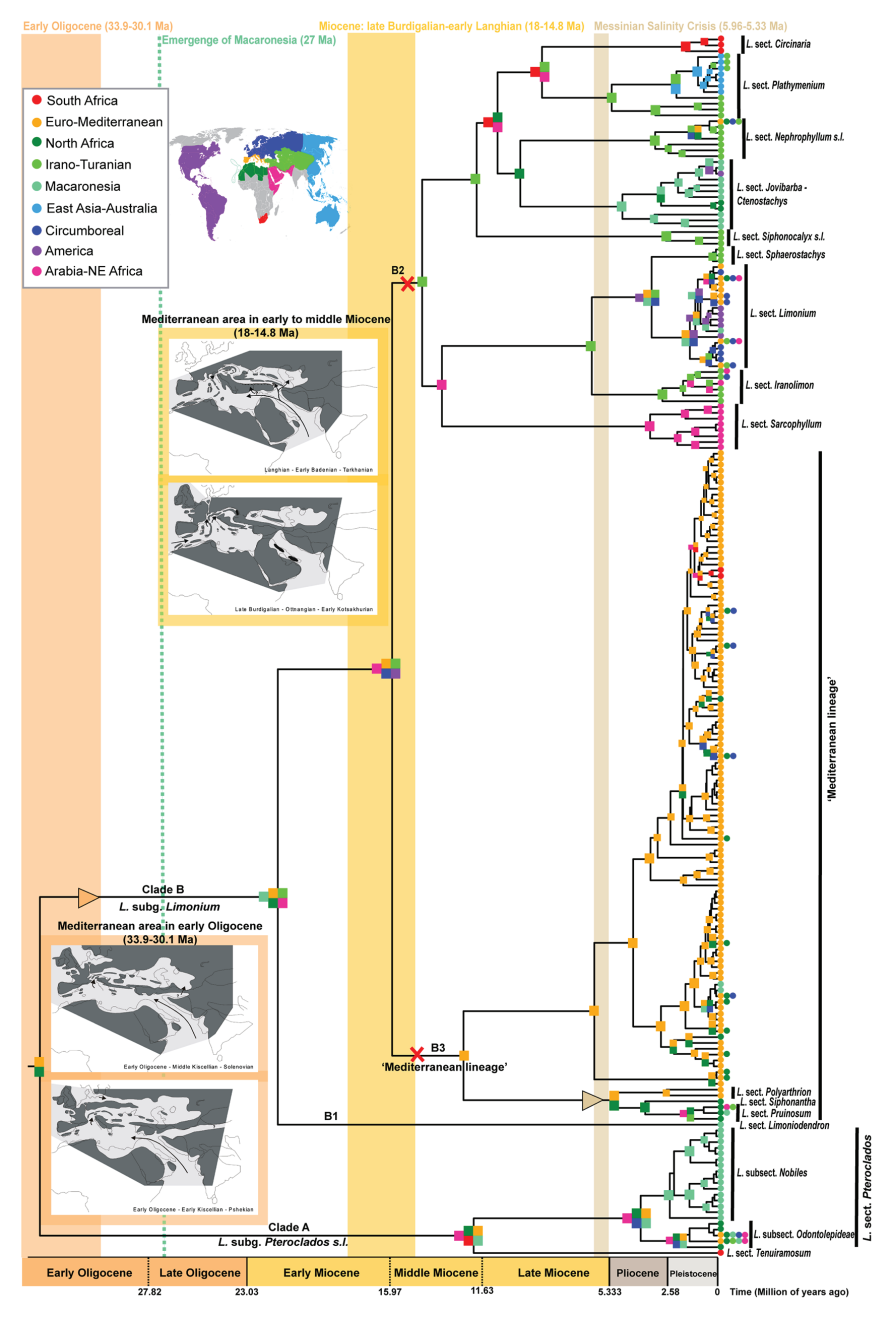
Time calibrated phylogeny and biogeography of *Limonium* based on “Supermatrix-ITS-like” dataset. The nine areas used for biogeographic inference are color coded as in the world map and corresponding list in the upper left inset. Colored squares on nodes indicate the most likely ancestral areas; due to space limitations, some nodes have no colored squares: in these cases, ranges are the same as in their respective ancestral nodes. Triangles and X’s along branches indicate range expansions and contractions, respectively, that occurred when geological events significantly impacted *Limonium*’s distribution and diversification (i.e., in the early Oligocene, early to middle Miocene and during the Messinian). These periods are marked by vertical color bands. In two of these periods, namely in the early Oligocene and the early to middle Miocene, paleomaps show the Mediterranean geographic configuration in each time frame (paleomaps are from the article of Rögl, 1999 published in Geologica Carpathica journal under the CC BY-NC-ND 4.0 license). Uncertainties in ancestral range estimates are in Supplementary Figure S4.

We estimated ancestral ranges for *Limonium* on “Supermatrix-ITS-like” and “Supermatrix-cpDNA-like” MCC trees using the R package BioGeoBEARS 1.1.2 (Matzke, 2013; R Core Team, 2018). Considering current species distributions, patterns of endemism, and the floristic regions occupied by the species, we identify nine major biogeographic areas: South Africa, Euro-Mediterranean, North Africa, Irano-Turanian, Macaronesia, East Asia-Australia, Circumboreal, America, and Arabia-NE Africa. Taxa were coded as present or absent in these areas (Figure 1 and Supplementary Table S3). We tested DEC (Dispersal-Extinction-Cladogenesis, Ree and Smith, 2008) and DIVA-like models (Dispersal-Vicariance Analysis, Ronquist, 1997). We chose not to use the BayArea-like model (Landis et al., 2013) since it does not allow for vicariance or “subset-within-area” speciation (allowed in DIVA-like and/or DEC models), which are both plausible considering species distributions and the broad areas defined in our study.

Time-stratified analyses with dispersal multiplier matrices were also employed to account for differences in dispersal probabilities due to changes in distances and connectivities between areas over time (Supplementary Table S4). In all biogeographic analyses, Macaronesia was allowed as an ancestral range only after 27 Ma, corresponding to the age of the oldest extant volcanic islands, i.e., the Selvagen Islands (Borges et al., 2008; Carine et al., 2010; Triantis et al., 2010; Fernández-Palacios et al., 2011). Considering the uncertainty surrounding the reconstruction of past sea levels (Miller et al., 2005; Rohling et al., 2014), we chose the age of 27 Ma as the most widely accepted, hence conservative estimate for the oldest emerged island in Macaronesia. Unlikely disjunct ranges (i.e., non-neighboring, geographically unconnected areas over the inferred existence of *Limonium*, starting ca. 40 Ma) were removed from the list of possible states. All models were also run with *j* parameter, which accounts for founder-event speciation. However, due to criticism by Ree and Sanmartín (2018) concerning “degenerate” inferences of +*j* models and inappropriate statistical comparisons of models with and without *j*, and considering that our inferences with and without *j* parameter closely match each other, we present only results of models without *j* and compare their likelihoods using AIC and AICc. We additionally estimated the type and number of biogeographical events using Biogeographical Stochastic Mapping (BSM; Matzke, 2016; Dupin et al., 2017) in BioGeoBEARS 1.1.2, providing the best-fitting model for each MCC tree and summarizing event frequencies over 200 discrete stochastic maps.

At a finer biogeographic scale, we investigated the origins of island endemics in Macaronesia and analyzed a clade (*Jovibarba-Ctenostachys* clade) comprising endemics in the Canaries, Cape Verde, Morocco, and Hispaniola in the Caribbean (four coded areas). Since the *Jovibarba-Ctenostachys* clade shows topological differences between datasets, we pruned it from cpDNA, ITS and “Supermatrix” MCC trees, ran DEC, DIVA-like, and BayArea-like models and compared results for all three trees. In contrast to the genus-wide biogeographic analyses, we included also the BayArea-like model for the biogeographic analyses of the *Jovibarba-Ctenostachys* clade because the absence of vicariance and “subset-within-area” speciation of this model was not considered a limitation for a lineage consisting exclusively of endemics, most of which are insular. Their biogeographic history could only be explained by dispersal, extinction, and sympatric speciation, all of which are accounted for in the BayArea-like model.

### Diversification Analyses

We estimated speciation and extinction rate dynamics of *Limonium* applying recently developed methods to both “Supermatrix-ITS-like” and “Supermatrix-cpDNA-like” MCC trees. First, we tested for shifts in diversification rates along tree branches and through time, using Bayesian Analysis of Macroevolutionary Mixture (BAMM 2.5; Rabosky et al., 2014). Sampling fractions per *Limonium* clade/section (following the classification of Koutroumpa et al., 2018: Supplementary Table S3) were provided in order to account for missing species (Supplementary Table S5). The R package BAMMtools 2.1.6 (Rabosky et al., 2014) was used to choose appropriate priors for BAMM analyses, analyze, and visualize results. BAMM was run with four independent chains of 100 million generations each and convergence was confirmed with ESS >200 for all parameters. The absence of recent, comprehensive monographic treatments for *Limonium* prevents us from performing analyses that account for potential discrepancies among different taxonomic treatments (e.g., Faurby et al., 2016). Moreover, taxonomic uncertainties could especially affect the apomictic species of the “Mediterranean lineage” (Haveman, 2013; Majeský et al., 2017). Acknowledging that taxonomic uncertainties could affect estimates of diversification rate dynamics (Faurby et al., 2016; Fernández-Mazuecos et al., 2019), we ran preliminary sensitivity analyses in BAMM, where we arbitrarily assumed that the number of species for the “Mediterranean lineage” is only half of the currently accepted number of “Mediterranean lineage” species (i.e., ca. 200 vs. 400 species), and thus assigned a higher sampling fraction to this lineage to account for missing species in analyses of both MCC trees (i.e., ½ instead of ¼; the former being the fraction used in the preliminary analyses). The signal in our data was so strong that even in these preliminary analyses that underestimated the number of species in the “Mediterranean lineage,” a shift in rates was recovered for this lineage in both MCC trees, despite the extreme and rather unrealistic taxonomic scenario.

Second, we tested for episodic tree-wide rate shifts caused, for example, by a mass extinction event, such as the one hypothesized for the temporal gap of diversification from the MSC to the onset of the Mediterranean climate in other Mediterranean plant lineages. We used the CoMET algorithm implemented in the R package TESS 2.1 (Höhna et al., 2015) to test for signals of mass extinction events and for shifts in speciation and extinction rates over time. Changes in speciation and extinction are modeled to occur at discrete time points and affect all clades in a tree simultaneously (episodic birth-death). We ran models assuming one or two rate shifts and allowing mass extinction events. A global sampling fraction of *Limonium* was provided to account for sampling probability of extant taxa (“Supermatrix-ITS-like”: 0.35 and “Supermatrix-cpDNA-like”: 0.36). We also used the automatic stopping functionality and specified a minimum ESS of 1,000 to allow MCMC simulations to reach convergence.

Third, we fitted different diversification models with constant or varying speciation and/or extinction rates through time and in relation to paleoenvironmental variables (Morlon et al., 2010; Condamine et al., 2013) using the R package RPANDA 1.6 (Morlon et al., 2016). Analyses were repeated using different initial parameters and choosing the solution with the highest likelihood for each model. Models were compared using AICc and a multimodel inference approach was employed for parameter estimates of time-dependent analyses (Burnham and Anderson, 2002). According to Morlon et al. (2011), rate heterogeneity can affect diversification rate estimates if such heterogeneity is not accounted for. Thus, apart from fitting models to the entire tree, RPANDA allowed us to test and account for rate heterogeneity by fitting models to subclades for which shifts were inferred in BAMM analyses (see section Results) and to the remaining phylogeny after removing these subclades. Models with or without rate shifts were then compared using the AICc. Global or clade-specific sampling fractions were assigned in analyses of either the entire phylogeny or the subclades defined above (in heterogeneity analyses), respectively, to account for incomplete taxon sampling. Paleoenvironmental variables used in RPANDA analyses as potential correlates of diversification rates were (i) global temperature throughout the Cenozoic (Zachos et al., 2008), fitted to the entire tree or all parts of the tree in heterogeneity analyses and (ii) past sea-level estimates (Miller et al., 2005; Rohling et al., 2014), fitted to Mediterranean subclade(s) that comprise coastal species (heterogeneity analyses; see environmental curves in Supplementary Figure S3). Specifically, the recent sea-level reconstructions of Rohling et al. (2014) in the Mediterranean are limited to the past 5.3 Myr and thus were fitted to the temporally corresponding part of the Mediterranean subclade, requiring that a few early-diverging taxa were removed from the subclade.

### State-Dependent Diversification

We tested for the effect of reproductive system on diversification rates using several state-dependent speciation and extinction (SSE) models. We assembled available information on reproductive strategies, coding 175 *Limonium* taxa (out of the 216 taxa included in the trees) as sexual or apomictic based on the literature (see Supplementary Table S6). Tips with missing reproductive information were dropped from “Supermatrix-ITS-like” and “Supermatrix-cpDNA-like” MCC trees before analyses.

First, we used BiSSE (Maddison et al., 2007) implemented in the R package diversitree 0.9–11 (FitzJohn et al., 2009; FitzJohn, 2012) to evaluate 10 models in which speciation, extinction, and transition rates varied or remained equal between states; two of these models had zero extinction (pure-birth models). Since we lack information on reproductive system for many *Limonium* species, we conducted three analyses with three different sampling fractions for each state (sexual vs. apomictic) to account for missing species in the phylogeny and tested the robustness of the results. In the first analysis, we assumed that the same proportion of sexuals vs. apomicts sampled in the entire *Limonium* phylogeny also applies to unsampled taxa (i.e., global sampling fraction). In the other two analyses, we incorporated prior knowledge that all described apomicts are included in the “Mediterranean lineage” (while species in all other lineages are sexual) and assumed either 50–50 or 40–60% of sexuals vs. apomicts in this lineage; we also accounted for uneven sampling in major lineages of *Limonium* by using the “make.bisse.uneven” function. We consider the latter sampling scenario as more realistic, since the predominance of apomicts over sexuals in the Mediterranean has been documented by Brullo and Erben (2016). Finally, we accounted for confidence intervals of parameter estimates by running a Bayesian MCMC with 50,000 steps using an exponential prior (following FitzJohn, 2012) and the best-fitting model for each analysis. Chain convergence was checked using the R package coda (Plummer et al., 2006) and results were plotted after removing 5,000 steps as burn-in.

Second, due to criticism of BiSSE concerning incorrect assignments of rate differences for neutral observed states in phylogenies, where rate heterogeneity is caused by other factors (Maddison and FitzJohn, 2015; Rabosky and Goldberg, 2015), we applied the Hidden SSE (HiSSE; Beaulieu and O’Meara, 2016) model to specifically account for other unmeasured factors impacting diversification rates. We tested seven models (model details in Beaulieu and O’Meara, 2016): a full BiSSE-like model, two HiSSE models with equal or varying transition rates (one rate for transitions among hidden states and two rates for transitions between sexual reproduction and apomixis), and four character-independent diversification (CID) models assuming that diversification is trait-independent but not constant across the tree (CID-2 and CID-4 models match complexity of BiSSE and HiSSE models, respectively). We also used the same three sampling scenarios for sexuals vs. apomictic states explained above, but without assigning uneven lineage-specific sampling probabilities (not implemented in HiSSE). Furthermore, we inferred the model-averaged rate estimates for all tips in both MCC trees and for all analyses to examine whether diversification rates varied between sexuals and apomicts. In BiSSE and HiSSE analyses, the tested models were compared with AIC.

Third, we used FiSSE (Rabosky and Goldberg, 2017) as a nonparametric test for reproductive system-dependent diversification in *Limonium*. This test is used for additional validation of results obtained from the other two state-dependent models (Rabosky and Goldberg, 2017). FiSSE shows the lowest “false positive” rates compared to BiSSE and HiSSE, but its power in detecting state-dependent diversification is much lower than BiSSE for trees with <300 tips (Rabosky and Goldberg, 2017). Although our trees are relatively small for the power limits of FiSSE, we expect that a significant state-dependent diversification, if detected, would show that the signal of our data is strong enough to overcome FiSSE’s power limitations. Conversely, finding non-significant state-dependent diversification does not allow the possibility that this result is due to FiSSE’s power limitations to be excluded, especially when the other two methods support state-dependent diversification. Here, we did not account for incomplete taxon sampling because this is not implemented in FiSSE.

Since most *Limonium* species occur in the Euro-Mediterranean area, we also tested the effect of this range on diversification rates using the fast Hidden Geographical SSE model (fGeoHiSSE; Caetano et al., 2018). *Limonium* species were coded as present only in the Euro-Mediterranean area or only in other areas or widespread in both the Euro-Mediterranean and other areas. Missing species were accommodated by providing sampling fractions for each of these three range categories. We fitted four models: two range-independent and two range-dependent diversification models, each either with or without a hidden area. Models were compared *via* AIC.

At shallower phylogenetic levels, we pruned the *Limonium* subgen. *Pteroclados s.l.* lineage (clade A in Figure 1; see also Koutroumpa et al., 2018) from “Supermatrix-ITS-like” and “Supermatrix-cpDNA-like” MCC trees, and tested whether woodiness (i.e., suffruticose habit) of Canarian *Limonium* endemics in *Nobiles* clade (Bramwell and Bramwell, 1974, 1990; Mesa et al., 2001; Marrero and Almeida, 2003; Kunkel, 2012; Jiménez et al., 2017) had an impact on diversification rates. In this fully sampled *Pteroclados s.l.* lineage, all other clades apart from *Nobiles* consist of herbaceous species. We used BiSSE and HiSSE analyses, with models described above, to test for habit-dependent diversification in the *Pteroclados s.l.* lineage. Simulation studies have suggested that while it is feasible to use SSE methods for analyses of small-size clades such as *Pteroclados s.l.* (e.g., Gamisch, 2016), their power in detecting state-dependent diversification depends on the strength of speciation rate asymmetry in the tree. If state-dependent diversification were detected, this would mean that speciation rate asymmetry between woody and herbaceous taxa is sufficiently high (i.e., >2.5-fold) to be detected despite small clade size, as demonstrated by simulations (Gamisch, 2016; Kodandaramaiah and Murali, 2018). Conversely, if state-independent diversification were supported, this would not automatically exclude the existence of moderate levels of speciation rate asymmetry (i.e., <2.5-fold), but might simply mean that such levels are insufficient to be recovered by the SSE methods in small clades. We also tested whether woodiness is a derived state for the *Pteroclados s.l.* lineage employing model-averaged ancestral state estimations implemented in HiSSE package.

## Results

### Molecular Phylogenies, Divergence Time, and Biogeographic Estimates

Phylogenies inferred with ML and BI for each dataset recovered very similar topologies. Only a small number of conflicts were found between cpDNA and ITS trees, all located within *Limonium* (i.e., “rogue clades”: subclades within “Mediterranean lineage,” *Circinaria* clade and *Jovibarba-Ctenostachys* clade, and six “rogue taxa”; see Supplementary Figures S1, S2 and Malekmohammadi et al., 2017; Koutroumpa et al., 2018). The 50% majority-rule BI trees for all three Supermatrices showed better resolution (“Supermatrix”: 181/300, “Supermatrix-ITS-like”: 177/293, and “Supermatrix-cpDNA-like”: 176/300 nodes resolved, i.e., have posterior probabilities ≥0.5) than ITS and cpDNA trees (154/259 and 142/298 nodes resolved, respectively). Our results strongly support monophyly for Plumbaginaceae subfamilies (Plumbaginoideae and Limonioideae), tribes (Aegialitideae and Limonieae) and genera (except for *Plumbago* and *Acantholimon*; both non-monophyletic), and for major clades of *Limonium* corresponding to infrageneric subdivisions (except for section *Schizhymenium*). Hereafter, results for “Supermatrix-ITS-like” tree and “Supermatrix-cpDNA-like” tree will be reported first and second, respectively, separated by a vertical bar, unless otherwise specified.

Divergence time estimates were almost identical between “Supermatrix-ITS-like” and “Supermatrix-cpDNA-like” MCC trees (detailed age estimates and HPDs in Table 1; Supplementary Table S2 and Supplementary Figures S1, S2). The origin of Plumbaginaceae is placed between the Upper Cretaceous and the early Eocene (95% HPD: 52–77 Ma), with a median age at early Paleocene (ca. 65.7 Ma). The median crown ages for Plumbaginoideae and Limonioideae are ca. 29.5 Ma (95% HPD: ca. 18–43 Ma) and ca. 57 Ma (95% HPD: ca. 43–71 Ma), respectively. Combining divergence time and biogeographic estimates for *Limonium* (DEC without dispersal-multiplier matrices selected as best-fitting model; Figure 1; Supplementary Table S7 and Supplementary Figures S4, S5), we infer a late Paleogene origin (ca. 33 Ma median crown age; 95% HPD: ca. 24–44 Ma) in the proto-Mediterranean region (i.e., Euro-Mediterranean and North-Africa|North Africa: most likely states, yet with low probability) for the genus. The two subgenera *Pteroclados s.l.* and *Limonium* originated during Miocene, ca. 12 Ma (95% HPD: ca. 6–20 Ma) and ca. 21 Ma (95% HPD: ca. 15–29 Ma), respectively, within widespread regions. A widespread range was also inferred (with low to moderate probabilities) for the most recent common ancestor (MRCA) of clades B2 (comprising mostly non-Mediterranean species) and B3 (“Mediterranean lineage”) in the middle Miocene (ca. 15.5 Ma; 95% HPD: ca. 11–21 Ma). This widespread ancestor gave rise to an endemic Euro-Mediterranean MRCA of the “Mediterranean lineage” (B3 crown-node; ca. 12 Ma) and either an endemic Irano-Turanian MRCA for the mostly non-Mediterranean B2 clade according to “Supermatrix-ITS-like” tree (ca. 14.4 Ma; Figure 1; Supplementary Figure S4) or a widespread MRCA for the B2 clade in the same ancestral widespread range according to “Supermatrix-cpDNA-like” tree (ca. 14 Ma; Supplementary Figure S5). In clade B2, dispersals, vicariance events and/or range contractions from middle Miocene through Plio-Pleistocene produced ancestors with endemic ranges in the Irano-Turanian, Arabia-NE Africa, East Asia-Australia, South Africa, America, and Macaronesia. Early divergence events of the “Mediterranean lineage” (clade B3) in late Miocene-early Pliocene were accompanied by dispersal from Euro-Mediterranean to North Africa, while the origin of the larger subclade of B3 (ca. 6 Ma median crown age) coincides with the MSC. Extensive speciation within the Euro-Mediterranean area during the Pleistocene was inferred for clade B3, with only little dispersal from Euro-Mediterranean to Macaronesia, North-Africa, Circumboreal and South Africa. Some of these biogeographic results should be interpreted with caution given the limited phylogenetic resolution within the “Mediterranean lineage.” Detailed nodal biogeographic reconstructions and uncertainties in ancestral range estimates for both trees are in Supplementary Figures S4, S5.

**Table 1:** Divergence time estimates for major clades in the “Supermatrix-ITS-like” and “Supermatrix-cpDNA-like” maximum clade credibility (MCC) trees.

Crown-nodes	Median ages (95% HPD)	
	“Supermatrix-ITS-like”	“Supermatrix-cpDNA-like”
Plumbaginaceae-Polygonaceae split (Root)	75.69 (69.03–78.21)	75.78 (69.18–78.21)
Plumbaginaceae	65.68 (52.01–77.03)	65.68 (52.07–77.2)
Plumbaginoideae	29.42 (18.71–42.76)	29.6 (18.36–43.23)
Limonioideae	56.92 (42.83–70.6)	57.2 (42.56–70.65)
Limonieae	40.35 (29.88–51.58)	40.1 (29.65–51.22)
*Limonium*	33.07 (23.68–44.23)	33.18 (23.39–43.76)
*Limonium* subg. *Pteroclados s.l.*	11.89 (6.25–19.97)	11.82 (6.2–19.45)
*Limonium* sect. *Pteroclados*	3.74 (2.11–5.86)	3.71 (2.11–5.82)
*Limonium* sect. *Pteroclados* subsect. *Nobiles*	2.32 (1.31–362)	2.29 (1.29–3.54)
*Limonium* subg. *Limonium*	21.46 (14.79–29.74)	21.12 (14.69–28.96)
*Limonium* clades B2-B3 split^*^	15.87 (11.11–21.52)	15.56 (11.12–21.12)
*Limonium* clade B2^*^	14.41 (10.03–19.57)	14.12 (9.89–19.05)
*Limonium* clade B3: “Mediterranean lineage”^*^	12.39 (8.09–17.33)	12.23 (8.05–16.93)
Larger subclade of “Mediterranean lineage”^**^	6 (3.72–8.99)	5.68 (3.41–8.46)

Median ages and 95% Highest Posterior Density (HPD) intervals (in parentheses) for the crown-nodes of clades are in million years (Ma). Dating results are from analyses with uniform priors assigned to Plumbaginaceae stem-node (secondary calibration).

* Coding of clades within *Limonium* follow Koutroumpa et al. (2018).

** The subclade has slightly different species composition between the two Supermatrices, due to the incongruent position of some Aegean species (for details see Koutroumpa et al., 2018).

**Figure 2:**
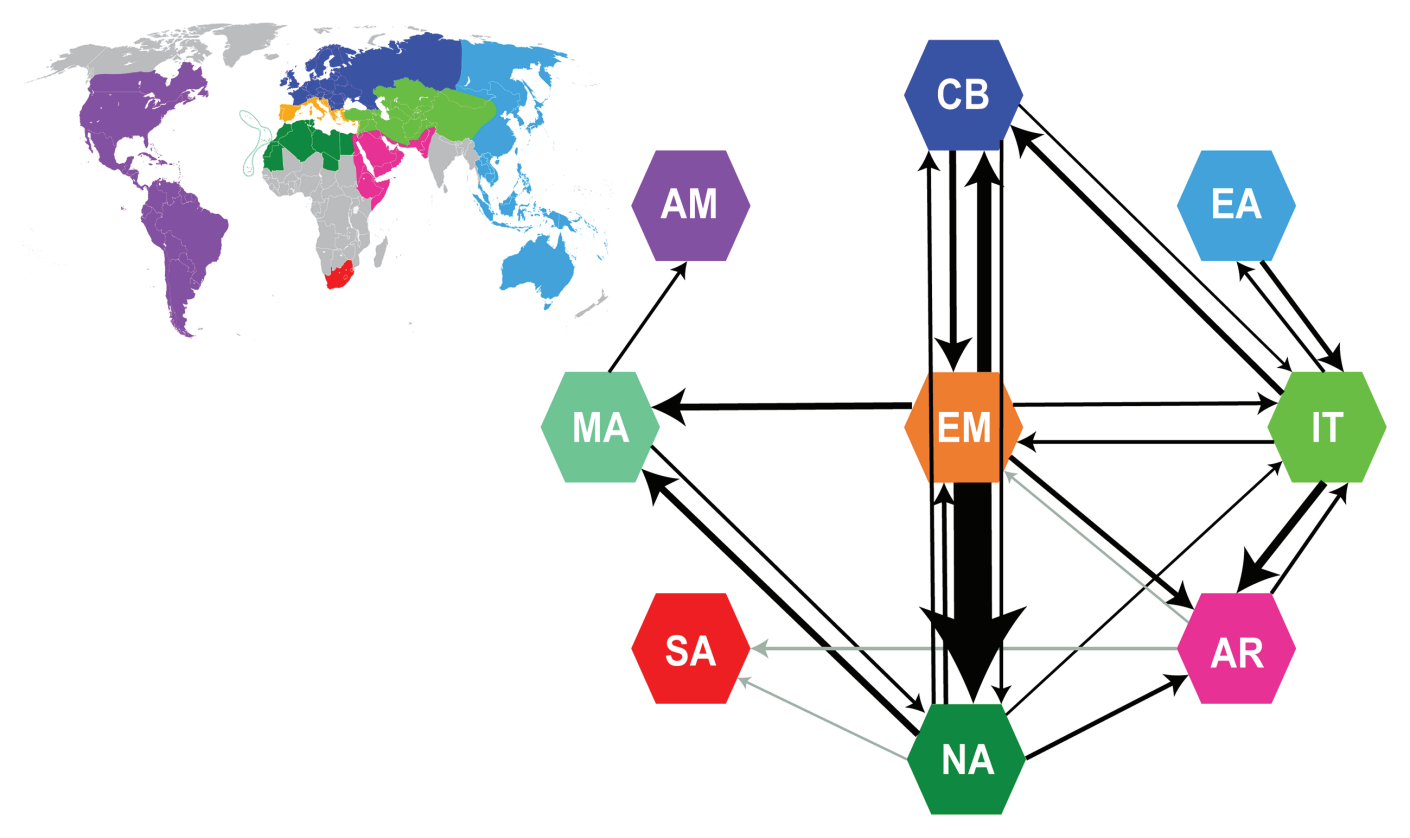
Summary of dispersal events from Biogeographical Stochastic Mapping (BSM) analyses. Arrows and their thickness represent the direction and frequency of dispersals between areas, respectively. Only event counts with a mean of ≥0.9 in both trees (“Supermatrix-ITS-like” and “Supermatrix-cpDNA-like”) are depicted as arrows (detailed results of BSM analyses in Supplementary Table S8). Gray arrows represent dispersal events with a mean ≥0.9 in only one of the two trees. The nine areas used in BSM analyses are color coded as in the world map in the upper left inset (same color coding of areas as in Figure 1). AM, America; AR, Arabia-NE Africa; CB, Circumboreal; EA, East Asia-Australia; EM, Euro-Mediterranean; IT, Irano-Turanian; MA, Macaronesia; NA, North Africa; SA, South Africa.

BSM analyses estimated 191|196 (SD: ±8| ± 7.79) within-area speciation events, 67|63 (±2.71| ± 2.06) range expansion events and 16|18 (±2.18| ± 1.71) vicariance events. The large number of sympatric speciation events (ca. 70% of all biogeographic events) was expected, given the large size of the defined areas. Most dispersal events occurred from Euro-Mediterranean to North Africa (ca. 14) and from Euro-Mediterranean to Circumboreal (ca. 5), while all other combinations of areas had <3.5 events (Figure 2; Supplementary Table S8). Overall, the major source area for dispersals was the Euro-Mediterranean (ca. 40% of estimated dispersal events), followed by North Africa (ca. 15%) and Irano-Turanian (ca. 14%), while the most common sink was North Africa (ca. 28%), followed by Circumboreal (16%). The longest-distance dispersals occurred from Macaronesia to the Americas, and from Arabia-NE Africa and North Africa (or Euro-Mediterranean region) to South Africa (Supplementary Table S8). Movements between areas were largely asymmetrical for most pairs of areas. For example, dispersals from Euro-Mediterranean to North Africa were ca. eight times more common than those in the opposite direction.

**Figure 3:**
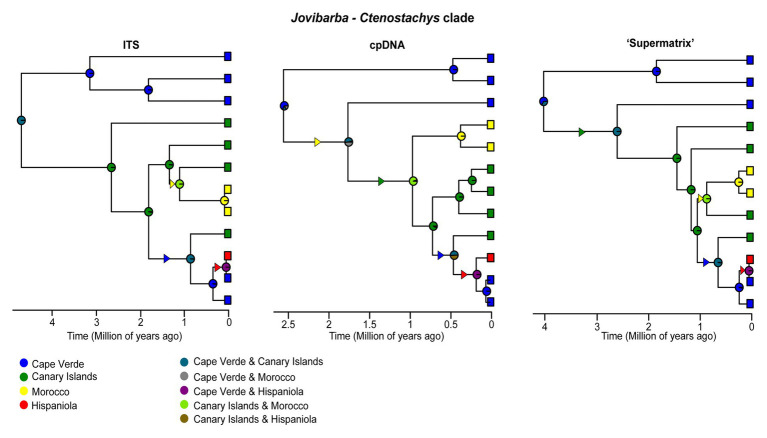
Ancestral range estimates for the *Jovibarba – Ctenostachys* clade of *Limonium* cropped from the ITS, cpDNA and “Supermatrix” MCC trees. The four areas used for biogeographic analyses and their combined ranges are color coded as in the left and right column, respectively, of the inset at the bottom. Pie charts on the nodes of trees represent relative probabilities of the inferred ancestral ranges. Triangles along branches indicate range expansions and their colors indicate the colonized area.

Focusing on island biogeography in Macaronesia, origins of island endemics included both neighboring continents and archipelagos (Supplementary Figures S4, S5; Figure 3). Macaronesian endemics were placed in five distinct clades, indicating at least five independent long distance dispersal (LDD) events (Supplementary Figures S4, S5). The *Nobiles* clade, comprising 16 Canarian endemics, originated ca. 2.3 Ma, while colonization of the Macaronesian area predated the origin of this clade and occurred from a widespread ancestor (Euro-Mediterranean and North Africa|Euro-Mediterranean, North and South Africa, Circumboreal and Arabia-NE Africa) between late Oligocene and early Pliocene. The Canarian endemic *L. dendroides* (clade B1) represents an early diverged lineage (ca. 21 Ma) that originated *via* dispersal from neighboring areas (i.e., North Africa or Euro-Mediterranean). The same dispersal pattern holds true for the closely related *L. bollei* and *L. lowei* (nested within “Mediterranean lineage”) endemic to Canaries and Madeira, respectively, which diversified in the late Pleistocene. The Azorean endemic (*L. eduardi-diasii*) originated in the late Pleistocene in a clade comprising American endemics, either *via* a recent colonization from the Americas (“Supermatrix-cpDNA-like”) or an earlier colonization (late Miocene-Pliocene) following range expansion from Irano-Turanian toward Euro-Mediterranean, Circumboreal, Macaronesia, and the Americas (“Supermatrix-ITS-like”). Fine-scale biogeographic estimations for the *Jovibarba-Ctenostachys* clade (DIVA-like selected as best-fitting model; Figure 3; Supplementary Table S9) that diversified during Plio-Pleistocene revealed two independent colonizations of Cape Verde (one probably from North Africa and the other from Canaries) and a LDD event from Cape Verde to Hispaniola. In this clade, the biogeographic origin of Canarian endemics is either from North Africa or Cape Verde and for Moroccan endemics either from Canaries or Cape Verde (inferences varied due to unresolved relationships). BSM reconstructs an overall total of seven dispersal events to Macaronesia (giving rise to native Macaronesian species as well as endemics) including two from North Africa and two from the Euro-Mediterranean. Furthermore, Macaronesia is inferred to be the source pool for four colonizations of continental areas, including one to the Americas and one to North Africa (Figure 2; Supplementary Table S8).

### Diversification Dynamics

**Figure 4:**
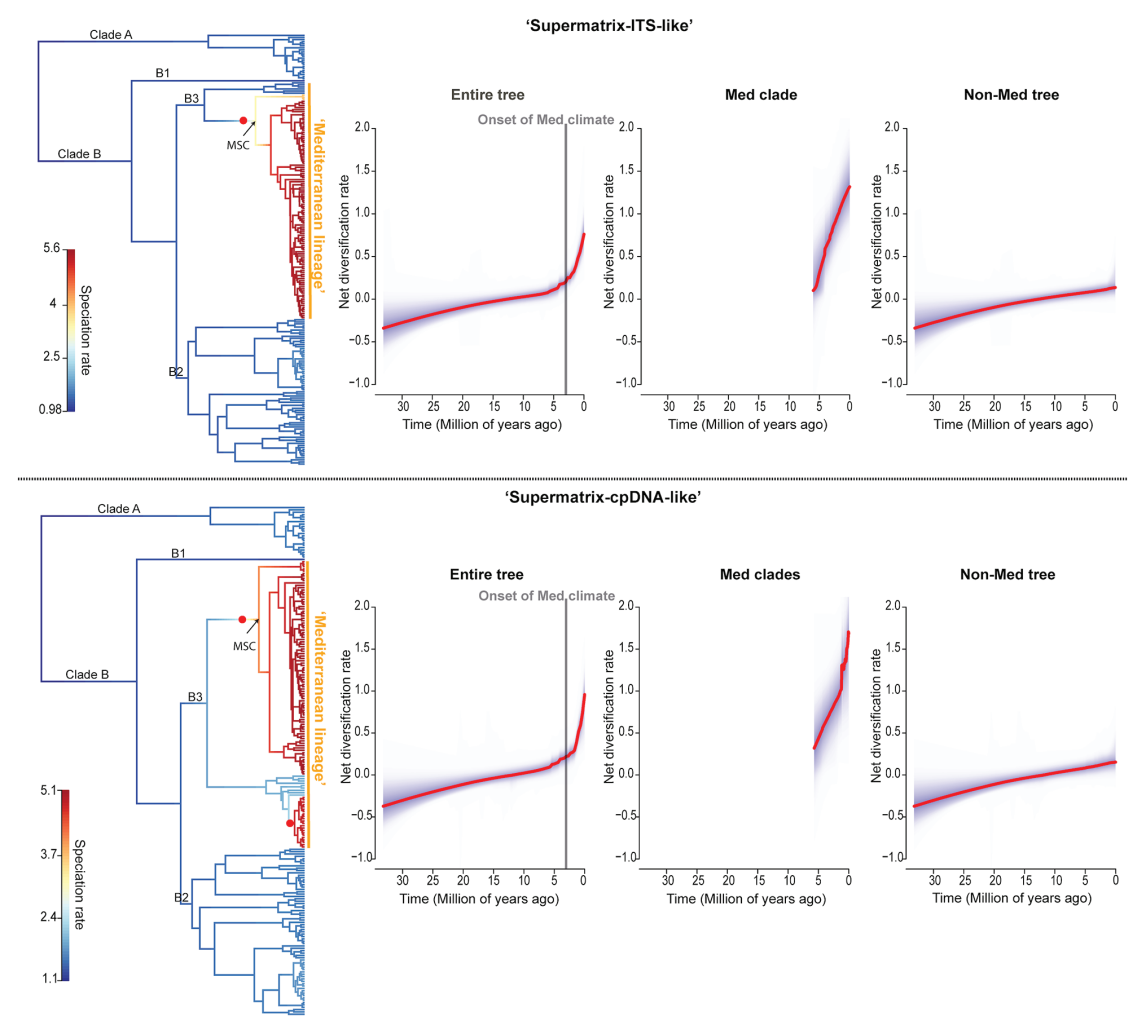
Diversification dynamics of *Limonium* across clades and through time. Results of BAMM analyses for “Supermatrix-ITS-like” and “Supermatrix-cpDNA-like” trees are presented in the top and bottom row, respectively. The first figure at the left of each row shows rate heterogeneity across clades for *Limonium* (phylorate plot) and the other three figures show the net diversification rates through time (rate-through-time plots). In the phylorate plots, branches are colored from blue to red to indicate low to high speciation rates, respectively, and the best rate-shift configuration is denoted by a red circle (the 95% credible set of rate shift configurations are in Supplementary Figure S7). The rate-through-time plots show the net diversification rates for the entire *Limonium* phylogeny (“Entire tree”), the Mediterranean clade(s) for which a shift was detected by BAMM (“Med clade(s)”), and the remaining *Limonium* phylogeny after removing the Mediterranean clade(s) (“Non-Med tree”). Significant shift(s) in diversification rates were found within the “Mediterranean lineage” of *Limonium*, while the steep increase in net diversification rates over the past 2–3 Myr is concomitant with the Plio-Pleistocene climatic oscillations and the establishment of the Mediterranean climate. MSC, Messinian Salinity Crisis.

BAMM analyses strongly reject constant diversification along clades and through time for *Limonium* (Supplementary Figure S6). Instead, they reveal significant shift(s) toward higher net diversification rates within the “Mediterranean lineage” (Figure 4; Supplementary Figure S7). In both trees, there is a shift close to the origin of the larger subclade of this lineage (crown age of subclade: ca. 6 Ma), while an additional shift within the smaller subclade for “Supermatrix-cpDNA-like” tree is explained by the conflicting topologies obtained from plastid and nuclear genomes for some Aegean species. Further evidence supports an 18–20-fold increase in net diversification rate for Mediterranean subclades compared to background rate (1.07 vs. 0.06|1.18 vs. 0.06). The rate-through-time plots show an increase in net diversification rates starting ca. 6 Ma that intensifies during the past 2–3 Myr (Figure 4).

TESS analyses found no significant mass extinction event or episodic change of speciation or extinction rates that could concurrently affect all clades of *Limonium* (Bayes Factors: 2lnBF<6; Supplementary Figure S8). RPANDA analyses of rate heterogeneity further corroborated results from BAMM. Models with rate shift(s), allowing distinct patterns of rate variation for the “Mediterranean lineage” subclade(s), were strongly supported over models assuming no rate shift for *Limonium* (*Δ*AICc>30; Table 2). We inferred a ca. 30-fold higher net diversification rate for the larger “Mediterranean lineage” subclade compared to the background rate (1.21 vs. 0.04|1.11 vs. 0.04; Table 2). For this subclade, a model of constant speciation and exponentially decreasing extinction over time (“Supermatrix-ITS-like”) or a pure-birth model with exponentially increasing speciation over time (“Supermatrix-cpDNA-like”) had the lowest AICc values (Supplementary Table S10). In the slightly reduced version of this subclade (with Rohling et al.’s, 2014 sea-level data fitted), the highest support was assigned to a model with constant speciation and extinction positively correlated to past sea-level for “Supermatrix-ITS-like,” while for “Supermatrix-cpDNA-like” that the model was the second best-fitting after a pure-birth model (Supplementary Table S10). For the backbone tree [i.e., remaining tree after removing “Mediterranean lineage” subclade(s)], a constant birth-death model received the lowest AICc values, while for the entire tree a model with exponentially varying speciation and extinction, both positively correlated to paleo-temperature, was best-fitting. In all analyses, we observed model uncertainty, with several alternative models explaining diversification dynamics (i.e., ΔAICc<2; Supplementary Table S10). Paleoenvironment-dependent models always received some support, with past temperature and sea-level having similar impacts on speciation and extinction rates.

**Table 2:** Diversification rate heterogeneity analyses in *Limonium* for both “Supermatrix-ITS-like” and “Supermatrix-cpDNA-like” trees (RPANDA analyses).

A				
“Supermatrix-ITS-like” tree	No rate shift	Heterogeneity analysis One rate shift (Med subclade)	ΔAICc	
Log-likelihood	−257.734	−241.163		
AICc	523.664	492.679	30.985	
“Supermatrix-cpDNA-like” tree	No rate shift	Heterogeneity analysis Two rate shifts (Med subclades)	ΔAICc	
Log-likelihood	−247.652	−227.627		
AICc	503.493	468.141	35.352	
B				
“Supermatrix-ITS-like” tree				
	Entire tree	Med subclade	Non-Med tree	
Speciation rate	4.86	6.91	3.07	
Extinction rate	4.75	5.70	3.03	
Net diversification rate	0.11	1.21	0.04	
“Supermatrix-cpDNA-like” tree				
	Entire tree	Med1 subclade	Med2 subclade	Non-Med tree
Speciation rate	5.17	5.46	9.18	3.27
Extinction rate	5.08	4.35	3.23	3.23
Net diversification rate	0.09	1.11	5.95	0.04

**(A)** Likelihoods and AIC comparisons between a model assuming no shifts across the tree (“No rate shift”) and a model assuming one and two rate shifts for “Supermatrix-ITS-like” and “Supermatrix-cpDNA-like” tree, respectively (“Heterogeneity analysis”); **(B)** Multimodel averaged speciation, extinction and net diversification rate estimates of time-dependent diversification analyses on the entire tree, the Mediterranean subclade(s) (“Med subclade” and “Med1 subclade”: the larger Mediterranean subclade in “Supermatrix-ITS-like” and “Supermatrix-cpDNA-like” trees, respectively, for which a shift was detected by BAMM; “Med2 subclade”: the small Mediterranean subclade for which a shift was detected by BAMM in “Supermatrix-cpDNA-like” tree) and the tree after removing the Mediterranean subclade(s) (“Non-Med tree”).

**Figure 5:**
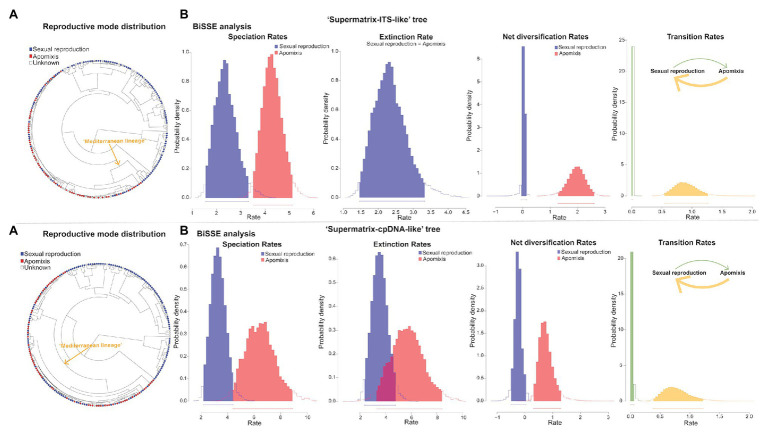
Diversification rate analyses in *Limonium* dependent upon apomictic vs. sexual reproduction. Results for “Supermatrix-ITS-like” and “Supermatrix-cpDNA-like” trees are presented in the top and bottom rows, respectively. **(A)** Phylogenetic distribution of sexual reproduction and apomixis. **(B)** Bayesian parameter inferences from BiSSE analyses employing the best fitting model under the sampling scenario that assumes a 40–60% of sexuals vs. apomicts in the “Mediterranean lineage.” For “Supermatrix-ITS-like” tree, a model with equal extinction rates was the best (see the single parameter estimate for both reproductive modes in the Extinction Rate graph in the top row) and for “Supermatrix-cpDNA-like” tree, a model with all rates varying was the best (Supplementary Table S11). The results show that speciation and net diversification rates are higher for apomictic than sexual taxa in both trees, extinction rates are also higher for apomicts but only in “Supermatrix-cpDNA-like” tree, while transitions from apomixis to sexual reproduction were more common that in the opposite direction.

BiSSE, HiSSE, and FiSSE analyses consistently supported the reproductive strategy-dependent diversification for *Limonium*, with apomixis leading to higher speciation and net diversification rates. In BiSSE, a model with varying speciation and transition rates and equal extinction rates between sexual reproduction and apomixis received the highest support in all analyses (i.e., for both MCC trees and different sampling scenarios), except for one analysis that supported a full BiSSE model (Supplementary Table S11). In most BiSSE analyses, up to two additional models received some statistical support (i.e., ΔAIC<2), yet all these alternative models supported the state-dependent diversification. Bayesian parameter estimations revealed higher speciation and net diversification rates for apomixis, and higher transition rates from apomixis to sexual reproduction than in the opposite direction (Figure 5). Additionally, higher extinction rate for apomixis was observed in the analysis supporting a full BiSSE model (Figure 5; Supplementary Table S11). In HiSSE, either a BiSSE-like model or a HiSSE model with three varying transition rates (see Section Materials and Methods) was supported in all analyses (Supplementary Table S12). Model-averaged parameter estimates for species in our trees recovered higher mean speciation, extinction, and net diversification rates for apomicts across all trees and analyses (Table 3). Given that a precise estimation of extinction from phylogenies of extant taxa is challenging (Pyron and Burbrink, 2013; but see Morlon et al., 2011 showing inference of realistic extinction rates), we focus our discussion on the general patterns rather than the estimates of extinction rates *per se* that need to be taken with caution. Complementary FiSSE analyses reported a ca. 2-fold higher speciation rate for apomixis (2.2|1.9 vs. 1.2|1.3), with the difference in rates being either significant (*p* = 0.016; “Supermatrix-ITS-like” tree) or only marginally non-significant (*p* = 0.0599; “Supermatrix-ITS-like” tree). Conversely, range-dependent diversification analysis (fGeoHiSSE) found no effect of presence in the Euro-Mediterranean area on diversification rates. Instead, analyses supported a model with diversification rate shifts across the tree, yet independent of the ranges (Supplementary Table S13).

**Table 3:** Mean speciation, extinction, and net diversification rate values from model-averaged estimates at the tips of each tree (“Supermatrix-ITS-like” and “Supermatrix-cpDNA-like”), as inferred from ancestral state reconstructions in HiSSE for the two MCC trees and under three different sampling scenarios of apomicts and sexuals.

“Supermatrix-ITS-like” tree		
Global Sampling Fraction		
	Sexuals	Apomicts
Speciation	4.18	7.57
Extinction	4.34	7.09
Net diversification	−0.16	0.47
Sampling Fractions with 50–50% sexuals vs. apomicts in “Mediterranean lineage”		
	Sexuals	Apomicts
Speciation	3.68	10.40
Extinction	4.22	9.87
Net diversification	−0.53	0.53
Sampling Fractions with 40–60% sexuals vs. apomicts in “Mediterranean lineage”		
	Sexuals	Apomicts
Speciation	2.96	9.88
Extinction	3.41	9.45
Net diversification	−0.46	0.43
“Supermatrix-cpDNA-like” tree		
Global Sampling Fraction		
	Sexuals	Apomicts
Speciation	4.27	7.99
Extinction	4.65	6.98
Net diversification	−0.38	1.01
Sampling Fractions with 50–50% sexuals vs. apomicts in “Mediterranean lineage”		
	Sexuals	Apomicts
Speciation	4.26	7.99
Extinction	4.65	6.97
Net diversification	−0.39	1.02
Sampling Fractions with 40–60% sexuals vs. apomicts in “Mediterranean lineage”		
	Sexuals	Apomicts
Speciation	3.40	12.27
Extinction	4.03	11.65
Net diversification	−0.63	0.62

**Figure 6:**
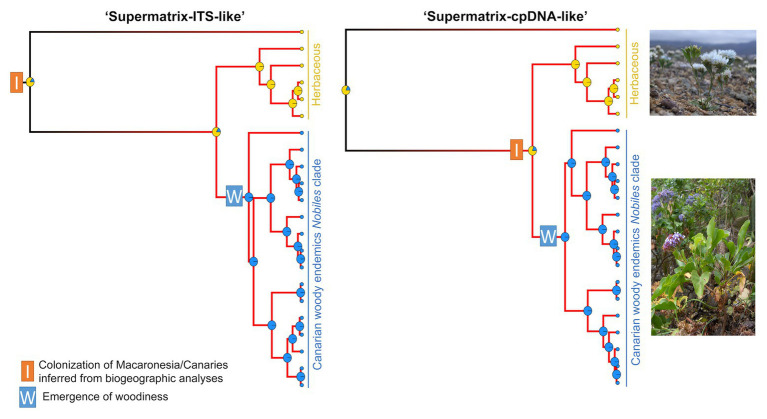
Evolution of habit in relation to island colonization for *Pteroclados s.l.* clade of *Limonium* from “Supermatrix-ITS-like” and “Supermatrix-cpDNA-like” MCC trees (model-averaged HiSSE analyses). Yellow at the tips and nodes denotes herbaceous habit and blue denotes woody habit. The pies at the nodes represent the relative probabilities of the inferred ancestral states. Net-diversification rates increase from black to red along the branches. Shift to woodiness followed island colonization but did not drive a significant increase of diversification rates. Photos of the herbaceous *L. lobatum* (top photo) and the woody-suffruticose *L. arboreum* (bottom photo) from Ares Jiménez.

State-dependent diversification analyses in *Pteroclados s.l.* clade showed no effect of habit (herbaceous habit vs. woodiness) on diversification rates (Supplementary Tables S14, S15). BiSSE analyses reported the lowest AIC for the constant rates model, although some state-dependent models with varying rates between woodiness and herbaceousness received partial support (i.e., ΔAIC<2). This result could be attributed to state-independent rate heterogeneity within *Pteroclados s.l.* clade, which BiSSE cannot test/account for, as revealed by HiSSE (i.e., the highest support for the CID-2 model with equal transition rates; Figure 6). However, caution should be taken when interpreting these results due to known limitations of SSE models in analyses of small-size clades such as the *Pteroclados s.l.* (see sections Materials and Methods and Discussion). Model-averaged ancestral state estimations combined with biogeographic results inferred woodiness as a derived state linked to insular life (Figure 6).

## Discussion

Identifying biotic and abiotic factors that drive the compartmentalization of biodiversity is central to biology (Benton, 2009, 2015). With its large size (ca. 600 species), wide geographic range (six continents), and species richness concentrated in the Mediterranean region (ca. 70% of all species), *Limonium* is a paramount example of uneven taxonomic and geographic plant diversity. Our macroevolutionary study on sea lavenders is one of the few to test the impact of both species-extrinsic and -intrinsic factors on diversification by using carefully selected methods of biogeographic and diversification-rate analyses (Lagomarsino et al., 2016; Condamine et al., 2018; Letsch et al., 2018), thus expanding the knowledge of the tempo and mode of plant diversification. We found significant increases of diversification rates associated with both geo-climatic events (namely, the MSC, onset of Mediterranean climate, and Pleistocene sea-level fluctuations) and intrinsic species traits, such as apomixis. The significant role of apomixis in shaping the Mediterranean radiation of *Limonium* had been previously proposed and is here explicitly tested for the first time. Our study provides new insights into the origins of Mediterranean biodiversity and highlights a significant role and interplay of both biotic and abiotic factors in promoting species diversification.

### Geological and Climatic Processes Shaping Species Distributions and Diversification

Our biogeographic and dating analyses of the largest *Limonium* phylogeny to date, combined with paleo-geological, paleo-climatic, and paleo-vegetational evidence (e.g., Rögl, 1999; Böhme, 2003; Thompson, 2005; Pound and Salzmann, 2017), allow us to propose likely processes that shaped the spatio-temporal evolution of the genus. Our analyses support the origin of *Limonium* in the late Paleogene (ca. 33 Ma) most probably in the proto-Mediterranean region (Figure 1; Table 1; Supplementary Figures S4, S5). During this period, major tectonic activities changed the configuration of Eurasia. Europe, formerly an archipelago during the Eocene, became more continental, and the Mediterranean Sea separated from the newly formed intercontinental Paratethys Sea (Rögl, 1999; Figure 1). The newly created land corridors may have facilitated the initial range expansion of *Limonium* from the Euro-Mediterranean into the Irano-Turanian region (Figure 1). Furthermore, the origin of *Limonium* in the early Oligocene is marked by a climatic transition from sub-tropical to more seasonal conditions along the Euro-Mediterranean coasts. At that time, vegetation representatives of seasonal climates (i.e., warm temperate conifer forests and sclerophyll woodland) spread from the Iberian Peninsula to the coasts of France, and xerophytic shrubland occurred for the first time in the central Mediterranean coasts partially replacing subtropical paleo-biomes formerly dominating the Mediterranean area (Pound and Salzmann, 2017).

The divergence of the “Mediterranean lineage” (clade B3) of *Limonium* from its mostly non-Mediterranean sister lineage (clade B2) during the Miocene (Table 1; Figure 1) follows a temporal pattern of divergence found in many other Mediterranean angiosperm lineages (Vargas et al., 2018) and is concomitant with paleo-geological and -climatic changes. These two largest lineages of *Limonium*, clades B2 and B3, diverged during the middle Miocene (ca. 15.5 Ma) from an ancestor that was widespread in the Euro-Mediterranean, Irano-Turanian, and other regions (Figure 1; Supplementary Figure S5). Prior to this split, in the early Miocene and specifically late Burdigalian, the Arabian plate that was connected to Africa collided with the Anatolian plate for the first time, cutting off the seaway between the Mediterranean Sea and the Indian Ocean (Figure 1). In the early Langhian and concomitant with the divergence between B2 and B3 clades, a marine transgression flooded the entire Mediterranean and Paratethys causing the re-opening of the seaway between South Anatolia and Arabia and the formation of other seaways in Eastern Anatolia and probably also in the Aegean or Western Anatolian regions (Rögl, 1999; Figure 1). These short-lived seaways may have enabled vicariant speciation followed by range contraction giving origin to a Euro-Mediterranean ancestor for clade B3 and an Irano-Turanian ancestor for clade B2 (“Supermatrix-ITS-like” tree; Figure 1), or peripatric speciation (i.e., temporary peripheral isolate in Euro-Mediterranean) giving origin to a Euro-Mediterranean ancestor for clade B3 and a widespread ancestor in the same ancestral range for clade B2 (“Supermatrix-cpDNA-like” tree; Supplementary Figure S5). Moreover, the early Langhian split between B2 and B3 clades occurred during the Mid-Miocene Climatic Optimum (i.e., a global warming event at ca. 17–15 Ma; Böhme, 2003) and specifically in a period of increased seasonality in Central Europe and the Mediterranean area (up to 6 dry months; Böhme, 2003; Thompson, 2005). At that time, the Mediterranean flora began to resemble the current vegetation due to the extinction of several tropical and subtropical elements in the region (Thompson, 2005).

The diverse Mediterranean flora is composed of a combination of elements that immigrated to the region from other areas, and those that arose *in situ* (Thompson, 2005). Elucidating the relative roles of repeated colonization vs. *in situ* speciation is necessary to understand the processes that shaped Mediterranean diversity. Our results show that the great majority of Euro-Mediterranean *Limonium* species have an autochthonous origin (ca. 100 sympatric speciation vs. ca. 7 dispersal events; see BSM results above), supporting Thompson’s (2005) hypothesis that *in situ* diversification contributed the main component of the heterogeneous Mediterranean flora. Additionally, the Euro-Mediterranean area received a few *Limonium* species, mostly from the neighboring Irano-Turanian, North African, and Circumboreal regions, consistent with previous studies on other taxa (e.g., Mansion et al., 2008, 2009; Salvo et al., 2010; Manafzadeh et al., 2014). The Euro-Mediterranean area also served as a major source of dispersals to other areas (see also tribe Antirrhineae: Gorospe et al., 2020), especially North Africa (Figure 2). Accordingly, diversity in this latter region was shaped mostly by dispersals (ca. 18 events, of which 14 from the Euro-Mediterranean area) and less by *in situ* speciation (ca. 3 events). Strong dispersal directionalities between areas (Figure 2) were also documented in Solanaceae by Dupin et al. (2017), who found a close relationship between species richness of an area and the number of dispersals from this area, a pattern congruent with our results. The inferred higher number of species dispersals from the Euro-Mediterranean “source” to the North African “sink” than to any other area could be explained by geoclimatic events that connected these two areas in the past, and by the availability of suitable habitats on their coastlines. Overall, the directional asymmetry of dispersals between the Euro-Mediterranean and other areas, consisting in more emigration than immigration to the region, suggests that extensive *in situ* speciation is the pump that promotes species movement from one main area (here the Euro-Mediterranean) to many others.

Proposed abiotic landmarks in the evolution of Mediterranean biodiversity include the Messinian Salinity Crisis (ca. 6–5.33 Ma), the onset of the Mediterranean climate (3.2–2.8 Ma), and Pleistocene geo-climatic fluctuations (Fiz-Palacios and Valcárcel, 2013). Indeed, we found that these events played a key role in the diversification of the “Mediterranean lineage” of *Limonium*. Although Mediterranean sea lavenders originated already in the middle Miocene (ca. 12 Ma; Figure 1; Table 1), all major extant lineages started to diversify only about 6 Ma concomitantly with the onset of the MSC, as a product of a significant, clade-specific shift toward higher diversification rates in the largest subclade of the “Mediterranean lineage” (phylorate plots; Figure 4). Thus, the salinity crisis that massively increased the availability of saline habitats across the Mediterranean represented an ecological opportunity for a salt tolerant genus like *Limonium* to experience a major episode of increased diversification rates. Conversely, the MSC apparently caused the extinction of other genera in the region, including the subtropical mangrove *Avicennia* (Thompson, 2005). Furthermore, the land corridors available across the Mediterranean at that time (see Mediterranean Paleogeographic Reconstruction During the MSC in Anzidei et al., 2014) might have provided venues for the colonization of North Africa from the Euro-Mediterranean (Figures 1, 2), or vice versa, as documented for *Borago* (Mansion et al., 2009). The refilling of the Mediterranean Sea at the end of the MSC may have promoted further vicariant speciation of *Limonium* in the Euro-Mediterranean and North Africa (Figure 1; see also Romeiras et al., 2016).

Diversification continued to increase exponentially also in the past 2–3 Myr, after the establishment of the Mediterranean climate and during the Pleistocene climatic oscillations (rate-through-time plots; Figure 4), corroborating results from other Mediterranean plant taxa (e.g., Valente et al., 2010; Fiz-Palacios and Valcárcel, 2011). Here, we explicitly tested the impact of paleo-temperature and past sea-level on diversification rates for the larger Mediterranean subclade. A pattern supported in all analyses was that extinction rates were higher when sea-level and temperature were higher (Supplementary Table S10). Additionally, our results supported heterogeneous, yet range-independent diversification of *Limonium* (Supplementary Table S13), demonstrating that occurrence in the Euro-Mediterranean region *per se* cannot explain the burst of diversification rates. Rather, we conclude that it was the combination of environmental changes experienced by *Limonium* in this region and intrinsic biotic traits (i.e., apomixis) that triggered the increase in diversification rates of the Mediterranean lineage, giving rise to its current high diversity of more than 400 species (see below).

### Apomixis as a Biotic Driver of Diversification and the Interplay Between Biotic and Abiotic Factors in Bolstering Diversification Rates

In explaining the origins of the Mediterranean flora, questions have been raised about the relative role of geological and climatic history, and intrinsic biological processes such as polyploidization and hybridization (Thompson, 2005). In *Limonium*, as in other angiosperms (Hörandl and Hojsgaard, 2012), polyploidy and hybridization are usually associated with apomixis (Erben, 1978, 1979), and the combined effects of these three biotic factors were proposed to have shaped the Mediterranean radiation species (e.g., Palacios et al., 2000; Lledó et al., 2005, 2011). While several apomictic *Limonium* species are characterized as obligate apomicts (e.g., Palacios and González-Candelas, 1997; Arrigoni and Diana, 1999; Palacios et al., 1999; Khan et al., 2012; Róis et al., 2016), facultative apomixis (i.e., occasional sexual reproduction in apomicts) has also been documented for some species (e.g., D’Amato, 1949; Hjelmqvist and Grazi, 1964; Artelari, 1989; Artelari and Georgiou, 2002; Georgakopoulou et al., 2006). At the macroevolutionary level, our analyses show for the first time that apomixis is associated with an acceleration of diversification rates. Speciation rates are consistently higher in apomictic than sexual taxa, while extinction rates are either equal or higher in apomicts (Figure 5; Table 3; Supplementary Table S11).

The higher extinction rate inferred for apomicts than sexuals is congruent with the labile nature of apomicts proposed by Darlington (1958) and Holsinger (2000), who regarded apomixis as a “blind alley of evolution” due to reduced recombination, hence lower genetic variation in apomicts, eventually driving them to extinction. This view was based on the assumption that apomixis is an irreversible, derived trait. However, our results suggest that transitions from apomixis to sexuality are very common in *Limonium* (Figure 5) and corroborate recent studies that support apomixis-to-sex reversals enabled by the usually facultative nature of apomixis in angiosperms (Hörandl et al., 2007; Hörandl and Hojsgaard, 2012; Hand and Koltunow, 2014; Hojsgaard et al., 2014; Hojsgaard and Hörandl, 2015; Carman et al., 2019; Sharma and Bhat, 2020). While the result that transitions from apomixis to sexuality are more common than vice versa is strongly supported for both MCC trees in *Limonium*, it should be interpreted with caution because the limited phylogenetic resolution in the “Mediterranean lineage” could affect the inferred transition rates. The molecular genetic basis of apomixis is unknown in *Limonium*. However, in some plant genera, the molecular pathway of apomixis seems to be superimposed onto the pathway of sexual reproduction, rather than being completely independent, thus apomixis can revert to sexuality relatively easily (e.g., Hand and Koltunow, 2014 and references therein). This explanation for the molecular basis of apomixis is consistent with the occurrence of both apomictic and sexually developed seeds in facultative apomicts, suggesting that, if an ovule fails to initiate the apomictic pathway, sexual reproduction is activated, since the sexual pathway remains available. Importantly, in addition to enabling apomixis-to-sex reversals, the occurrence of “a little bit of sex” in apomicts can prevent the genomic decay caused by the absence of meiotic recombination, which can lead to extinction (Hörandl and Hojsgaard, 2012; Hojsgaard and Hörandl, 2015; Hodač et al., 2019).

Our results point toward a synergistic relationship between apomixis and sea-level fluctuations in driving the diversification of Mediterranean sea lavenders. In the Euro-Mediterranean region, the majority of *Limonium* species occur in coastal areas (which are vast, including many islands). Geo-climatic changes caused intense sea-level oscillations that had the highest frequency during the Plio-Pleistocene (Supplementary Figure S3), directly impacting coastal habitats. High sea levels triggered inundation and loss of available coastal habitats for *Limonium*, thus increasing extinction (see positive correlation of extinction rates with past sea levels for the “Mediterranean lineage” in Supplementary Table S10). Additionally, when sea levels are high, some populations may split and diversify allopatrically. Conversely, low sea levels create new areas that allow populations to expand, occasionally coming in contact and hybridizing. The newly created polyploid hybrids can establish new populations by single individuals through apomixis, and further diversify (see Negative Correlation of Speciation Rates With Past Sea Levels for the “Mediterranean lineage” in Supplementary Table S10). Apomixis provides both the required escape from sterility for hybrid polyploids and reproductive assurance in the absence of pollinators or mates in newly colonized habitats (Baker, 1955; Darlington, 1958). Thus, sea-level fluctuations cause repeated events of species-range contraction and fragmentation when sea level is high, and expansion and reconnection when sea level is low, promoting the origin of incipient species that can become established through apomixis. Long-term survival of apomictic species can be enhanced through occasional sex enabling the filtering of deleterious mutations *via* purging selection (Hojsgaard and Hörandl, 2015; Hodač et al., 2019).

### Island Biogeography of Macaronesian Endemics and Insular Woodiness

Oceanic islands emerge from the sea empty of life. Their biodiversity is attributed to a combination of dispersals across the sea of species from neighboring areas and local diversification, producing high levels of endemicity (Cowie and Holland, 2006; Warren et al., 2015). *Limonium* endemics in Macaronesian archipelagos are of recent Plio-Pleistocene origin, except for *L. dendroides*, which diverged much earlier during the early Miocene (Supplementary Figures S4, S5). *Limonium dendroides* is an endangered Canarian endemic that is morphologically and taxonomically unique. It is characterized by at least two striking autapomorphies, i.e., arborescent habit and salt-glands in spikelet, and is the sole species of *L.* sect. *Limoniodendron* (Figure 1). The phylogenetic distinctiveness of *L. dendroides*, indicated by its isolated long branch sister to the large clade comprising all other species of subgenus *Limonium*, suggests that *L. dendroides* is either an old species or it is the only surviving species of a once larger clade that has undergone extensive extinction (Warren et al., 2018). Many plant taxa in Macaronesia comprise multiple endemic species that stemmed from a single long-distance dispersal followed by *in situ* diversification (e.g., Böhle et al., 1996; Francisco-Ortega et al., 1996; Barber et al., 2007; Kim et al., 2008; Caujapé-Castells, 2011). In *Limonium*, however, Macaronesian diversity was shaped by multiple LDD events (ca. 7), mostly from the neighboring Euro-Mediterranean and North African areas, followed by *in situ* speciation (ca. 22 events; BSM results). The Canarian and Cape Verde archipelagos were colonized repeatedly (at least four times and twice, respectively), as found in several studies of other Macaronesian taxa (Carine et al., 2004; Sanmartín et al., 2008 and references therein; Navarro-Pérez et al., 2015; Jaén-Molina et al., 2020). The availability of different ecological niches in oceanic islands may facilitate independent colonization by different species, possibly *via* reducing competition. For example, the two *Limonium* endemic lineages stemming from independent dispersals to Cape Verde occupy distinct habitats, namely wet mountainous cliffs and coasts (Romeiras et al., 2015). Thus, habitat heterogeneity of oceanic island systems favors colonization by a diversity of species pre-adapted to contrasting ecological niches.

While oceanic islands have been traditionally regarded as major sinks of biodiversity (Carlquist, 1974), their role as sources of biodiversity for neighboring continents or archipelagos has been rarely documented (e.g., Mort et al., 2002; Carine et al., 2004). Our results support four LDD events for *Limonium* from Macaronesia including at least one to North Africa, but also Trans-Atlantic LDD to the Americas (Figure 2; Supplementary Table S8). Fine-scale analysis of *Jovibarba-Ctenostachys* clade reveals a dispersal event from Macaronesia to Hispaniola and another to Morocco, and one or two inter-archipelago dispersals between Canaries and Cape Verde (Figure 3). These results highlight the significance of oceanic archipelagos in Macaronesia as a source flora for LDD to archipelagos within and outside the region, and to neighboring continents (see also Gruenstaeudl et al., 2017; Jaén-Molina et al., 2020). Furthermore, the noteworthy long-distance dispersal from Cape Verde across the Atlantic Ocean to Hispaniola (Caribbean) was probably facilitated by both the light diaspores of these species and sea currents, such as the North Equatorial Current, as suggested for other angiosperms (Renner, 2004). Our results further suggest that different dispersal properties in Macaronesian lineages may explain the geographically broad range of the *Jovibarba-Ctenostachys* clade vs. the within-archipelago range of the Canarian *Nobiles* clade. Indeed, reduced dispersal abilities have been documented for the latter clade (Jiménez et al., 2017), supporting the hypothesis of post-colonization loss of dispersal ability in colonists of oceanic islands followed by extensive *in situ* diversification (Carlquist, 1966; MacArthur and Wilson, 1967).

Woodiness is very common in oceanic islands (Darwin, 1859; Wallace, 1878; Carlquist, 1965, 1974; Lens et al., 2013; Burns, 2019). Two competing hypotheses about the origin of insular woodiness have been proposed, which differ in whether woodiness evolved before or after island colonization. The “islands-as-museums” hypothesis views woodiness as a relictual trait already present in continental taxa that colonized islands and later went extinct from the continent (Cronk, 1992, 1997). Alternatively, woodiness has been interpreted as a derived trait that evolved *in situ* from herbaceous colonists subsequent to island colonization (e.g., Nürk et al., 2019). Our results in *Limonium* support the latter hypothesis by showing that woodiness is a derived trait in *Pteroclados s.l.* that emerged after colonization of the Canaries (woody *Nobiles* clade; Figure 6). The derived nature of woodiness is also supported by most studies on woody insular plants (e.g., *Echium*: Böhle et al., 1996; *Lavatera*: Fuertes-Aguilar et al., 2002; *Sideritis*: Barber et al., 2002; *Sanctambrosia*: Kool and Thulin, 2017), while fewer studies support its relictual nature (e.g., *Tolpis*: Moore et al., 2002; *Descurainia*: Goodson et al., 2006).

Woodiness on oceanic islands has been regarded as a key innovation linked to higher diversification rates when it is associated with disparity in growth forms (e.g., arborescent shrubs, subshrubs, trees, cushion forms, woody lianas, and giant rosette plants), because it enables the exploration of a broader niche space (Nürk et al., 2019). However, our results suggest that insular woodiness in the Canarian *Nobiles* clade of *Limonium* is either not linked to accelerated diversification (Figure 6; Supplementary Tables S14, S5) or a shift in rates for woody vs. herbaceous taxa is too moderate for the SSE methods to detect it, considering their limited power in analyses of small-size clades (Gamisch, 2016; Kodandaramaiah and Murali, 2018). A possible explanation for the result that the evolution of woodiness on the Canary Islands did not trigger a significant shift to much higher diversification rates may be that, in Canarian *Limonium*, woodiness is not associated with a diversity of woody growth forms (as found by Nürk et al., 2019), since all species in *Nobiles* clade have similar subshrub suffruticose habit. The lack of diversity in woody forms observed in species of the *Nobiles* clade may have limited their ability to radiate in a way that can be detected as a significant shift of diversification rates.

## Conclusion

At the global scale, our study shows that the evolution of *Limonium* in space and time was shaped by major geologic and climatic processes that resulted in its cosmopolitan distribution and extensive diversification in the Mediterranean area. The increased diversification of Mediterranean sea lavenders was enabled by the ability of these taxa to reproduce asexually, *via* apomixis, which allowed them to survive and diversify during climatic oscillations. Our results show that the joint effect of biotic and abiotic factors is responsible for the current diversity of *Limonium*. At the regional scale, focusing on the insular endemics of Macaronesia, diversity on oceanic islands was shaped by multiple colonization events from neighboring continents and archipelagos, and *in situ* speciation. The Macaronesian islands have also served as sources for dispersals to other archipelagos (Caribbean) and continents (North Africa and America). In addition, woodiness in the Canarian sea lavenders (*Nobiles* clade) is a derived trait linked to insularity but not too much higher diversification rates. Our study highlights the importance of analyzing multiple abiotic and biotic factors, and their interactions, to achieve an in-depth understanding of evolution in hotspots of biodiversity.

## Data Availability Statement

The datasets presented in this study can be found in online repositories. The names of the repository/repositories and accession number(s) can be found in the article/Supplementary Material.

## Author Contributions

EC and KK contributed to conception and design of the study. AJ, BW, KK, and MR provided material and data for analyses. KK performed all analyses with help from BW, MC, and ST and wrote the first draft of the manuscript. All authors contributed to manuscript revision, read, and approved the final version.

### Conflict of Interest

The authors declare that the research was conducted in the absence of any commercial or financial relationships that could be construed as a potential conflict of interest.

## References

[ref1] Anzidei M. Lambeck K. Antonioli F. Furlani S. Mastronuzzi G. Serpelloni E. (2014). Coastal structure, sea-level changes and vertical motion of the land in the Mediterranean. Geol. Soc. Lond., Spec. Publ. 388, 453–479. 10.1144/SP388.20

[ref2] Arrigoni P. V. Diana S. (1999). Karyology, chorology and bioecology of the genus *Limonium* (Plumbaginaceae) in Sardinia. Plant Biosyst. 133, 63–71. 10.1080/11263509909381533

[ref3] Artelari R. (1989). Biosystematic study of the genus *Limonium* (Plumbaginaceae) in the Aegean area (Greece). I. some Limonium species from the Kikladhes islands. Willdenowia 18, 399–408.

[ref4] Artelari R. Georgiou O. (2002). Biosystematic study of the genus *Limonium* (Plumbaginaceae) in the Aegean area, Greece. III. Limonium on the islands Kithira and Antikithira and the surrounding islets. Nord. J. Bot. 22, 483–502. 10.1111/j.1756-1051.2002.tb01402.x

[ref5] Asker S. Jerling L. (1992). Apomixis in plants. Boca Raton, FL: CRC Press.

[ref6] Baker H. G. (1955). Self-compatibility and establishment after “long-distance” dispersal. Evolution 9, 347–349. 10.2307/2405656

[ref7] Baker H. G. (1966). The evolution, functioning and breakdown of heteromorphic incompatibility systems. I. the Plumbaginaceae. Evolution 20, 349–368. 10.2307/2406635, PMID: 28562969

[ref8] Barber J. C. Finch C. C. Francisco-Ortega J. Santos-Guerra A. Jansen R. K. (2007). Hybridization in Macaronesian *Sideritis* (Lamiaceae): evidence from incongruence of multiple independent nuclear and chloroplast sequence datasets. Taxon 56, 74–88. 10.2307/25065737

[ref9] Barber J. C. Francisco-Ortega J. Santos-Guerra A. Turner K. G. Jansen R. K. (2002). Origin of Macaronesian *Sideritis* L.(Lamioideae: Lamiaceae) inferred from nuclear and chloroplast sequence datasets. Mol. Phylogenet. Evol. 23, 293–306. 10.1016/S1055-7903(02)00018-0, PMID: 12099789

[ref10] Barnosky A. D. (2001). Distinguishing the effects of the red queen and court jester on Miocene mammal evolution in the northern Rocky Mountains. J. Vertebr. Paleontol. 21, 172–185. 10.1671/0272-4634(2001)021[0172:DTEOTR]2.0.CO;2

[ref11] Beaulieu J. M. O’Meara B. C. (2016). Detecting hidden diversification shifts in models of trait-dependent speciation and extinction. Syst. Biol. 65, 583–601. 10.1093/sysbio/syw022, PMID: 27016728

[ref12] Benton M. J. (2009). The red queen and the court jester: species diversity and the role of biotic and abiotic factors through time. Science 323, 728–732. 10.1126/science.1157719, PMID: 19197051

[ref13] Benton M. J. (2015). Exploring macroevolution using modern and fossil data. Proc. R. Soc. B 282:20150569. 10.1098/rspb.2015.0569, PMID: 26063844 PMC4590474

[ref14] Bessedik M. Guinet P. Suc J. P. (1984). Données paléofloristiques en Méditerranée Nord-occidentale depuis l'Aquitanien. Rev. Paléobiol. 25–31.

[ref15] Beucher F. (1975). Étude palynologique de formations néogènes et quaternaires au Sahara Nord-occidental. Vol. 20 Paris: Centre national de la recherche scientifique.

[ref16] Blondel J. Aronson J. (1999). Biology and wildlife of the Mediterranean region. New York: Oxford University Press.

[ref17] Blondel J. Aronson J. Bodiou J. Y. Boeuf G. (2010). The Mediterranean region: Biological diversity in space and time. New York: Oxford University Press.

[ref18] Böhle U. R. Hilger H. H. Martin W. F. (1996). Island colonization and evolution of the insular woody habit in *Echium* L. (Boraginaceae). Proc. Natl. Acad. Sci. U. S. A. 93, 11740–11745. 10.1073/pnas.93.21.11740, PMID: 8876207 PMC38128

[ref19] Böhme M. (2003). The Miocene climatic optimum: evidence from ectothermic vertebrates of Central Europe. Palaeogeogr. Palaeoclimatol. Palaeoecol. 195, 389–401. 10.1016/S0031-0182(03)00367-5

[ref20] Borges P. A. Abreu C. Aguiar A. M. F. Carvalho P. Jardim R. Melo I. (eds.) (2008). “A list of the terrestrial fungi, flora and fauna of Madeira and Selvagens archipelagos” in Direcção regional do Ambiente da Madeira and Universidade dos Açores. Horta: Angra do Heroísmo and Ponta Delgada.

[ref21] Bouchenak-Khelladi Y. Muasya A. M. Linder H. P. (2014). A revised evolutionary history of Poales: origins and diversification. Bot. J. Linn. Soc. 175, 4–16. 10.1111/boj.12160

[ref22] Bouchenak-Khelladi Y. Onstein R. E. Xing Y. Schwery O. Linder H. P. (2015). On the complexity of triggering evolutionary radiations. New Phytol. 207, 313–326. 10.1111/nph.13331, PMID: 25690582

[ref23] Boucher F. C. Zimmermann N. E. Conti E. (2016). Allopatric speciation with little niche divergence is common among alpine Primulaceae. J. Biogeogr. 43, 591–602. 10.1111/jbi.12652

[ref24] Bouckaert R. R. Drummond A. J. (2017). bModelTest: Bayesian phylogenetic site model averaging and model comparison. BMC Evol. Biol. 17:42. 10.1186/s12862-017-0890-6, PMID: 28166715 PMC5294809

[ref25] Bouckaert R. Vaughan T. G. Barido-Sottani J. Duchêne S. Fourment M. Gavryushkina A.. (2019). BEAST 2.5: an advanced software platform for Bayesian evolutionary analysis. PLoS Comput. Biol. 15:e1006650. 10.1371/journal.pcbi.1006650, PMID: 30958812 PMC6472827

[ref26] Bramwell D. Bramwell Z. I. (1974). Wild flowers of the Canary Islands. London: Stanley Thornes.

[ref27] Bramwell D. Bramwell Z. I. (1990). Flores silvestres de las Islas Canarias. Vol. 1 Madrid: Ed. Rueda.

[ref28] Brullo S. Brullo C. Cambria S. Del Galdo G. G. Minissale P. (2017). Phytosociological investigation on the class Crithmo maritimi-Limonieteain Greece. Plant Sociol. 54, 3–57. 10.7338/pls2017541/01

[ref29] Brullo S. Erben M. (2016). The genus *Limonium* (Plumbaginaceae) in Greece. Phytotaxa 240, 1–212. 10.11646/phytotaxa.240.1.1

[ref30] Burnham K. P. Anderson D. R. (2002). Model selection and multimodel inference. A practical information-theoretic approach. 2nd Edn. New York: Springer.

[ref31] Burns K. C. (2019). Evolution in isolation: The search for an island syndrome in plants. Cambridge: Cambridge University Press.

[ref32] Caetano D. S. O'Meara B. C. Beaulieu J. M. (2018). Hidden state models improve state-dependent diversification approaches, including biogeographical models. Evolution 72, 2308–2324. 10.1111/evo.13602, PMID: 30226270

[ref33] Carine M. A. Russell S. J. Santos-Guerra A. Francisco-Ortega J. (2004). Relationships of the Macaronesian and Mediterranean floras: molecular evidence for multiple colonizations into Macaronesia and back-colonization of the continent in *Convolvulus* (Convolvulaceae). Am. J. Bot. 91, 1070–1085. 10.3732/ajb.91.7.1070, PMID: 21653463

[ref34] Carine M. A. Santos-Guerra A. Guma I. R. Reyes-Betancourt J. A. (2010). “Endemism and evolution of the Macaronesian flora” in Beyond cladistics: The branching of a paradigm. eds. Williams D. M. Knapp S. (Oakland, CA: University of California Press), 101–124.

[ref35] Carlquist S. (1965). Island life; a natural history of the islands of the world. New York: The Natural History Press.

[ref36] Carlquist S. (1966). The biota of long-distance dispersal. III. Loss of dispersibility in the Hawaiian flora. Brittonia 18, 310–335. 10.2307/2805148

[ref37] Carlquist S. (1974). Island biology. New York: Columbia University Press.

[ref38] Carman J. G. Mateo de Arias M. Gao L. Zhao X. Kowallis B. M. Sherwood D. A.. (2019). Apospory and diplospory in diploid *Boechera* (Brassicaceae) may facilitate speciation by recombination-driven apomixis-to-sex reversals. Front. Plant Sci. 10:724. 10.3389/fpls.2019.00724, PMID: 31214233 PMC6555261

[ref39] Caujapé-Castells J. (2011). “Jesters, red queens, boomerangs and surfers: a molecular outlook on the diversity of the Canarian endemic flora” in The biology of island floras. eds. Bramwell D. Caujapé-Castells J. (Cambridge: Cambridge University Press), 284–324.

[ref40] Christenhusz M. J. Byng J. W. (2016). The number of known plants species in the world and its annual increase. Phytotaxa 261, 201–217. 10.11646/phytotaxa.261.3.1

[ref41] Comes H. P. (2004). The Mediterranean region-a hotspot for plant biogeographic research. New Phytol. 164, 11–14. 10.1111/j.1469-8137.2004.01194.x 33873489

[ref42] Condamine F. L. Rolland J. Höhna S. Sperling F. A. Sanmartín I. (2018). Testing the role of the red queen and court jester as drivers of the macroevolution of Apollo butterflies. Syst. Biol. 67, 940–964. 10.1093/sysbio/syy009, PMID: 29438538

[ref43] Condamine F. L. Rolland J. Morlon H. (2013). Macroevolutionary perspectives to environmental change. Ecol. Lett. 16, 72–85. 10.1111/ele.12062, PMID: 23331627

[ref44] Costa J. Torices R. Barrett S. C. (2019). Evolutionary history of the buildup and breakdown of the heterostylous syndrome in Plumbaginaceae. New Phytol. 224, 1278–1289. 10.1111/nph.15768, PMID: 30825331

[ref45] Cowie R. H. Holland B. S. (2006). Dispersal is fundamental to biogeography and the evolution of biodiversity on oceanic islands. J. Biogeogr. 33, 193–198. 10.1111/j.1365-2699.2005.01383.x

[ref46] Critical Ecosystem Partnership Fund (2019). Biodiversity hotspots. Available at: https://www.cepf.net/our-work/biodiversity-hotspots (Accessed November 15, 2019).

[ref47] Cronk Q. C. B. (1992). Relict floras of Atlantic islands: patterns assessed. Biol. J. Linn. Soc. Lond. 46, 91–103. 10.1111/j.1095-8312.1992.tb00852.x

[ref48] Cronk Q. C. B. (1997). Islands: stability, diversity, conservation. Biodivers. Conserv. 6, 477–493. 10.1023/A:1018372910025

[ref49] Crowl A. A. Visger C. J. Mansion G. Hand R. Wu H. H. Kamari G.. (2015). Evolution and biogeography of the endemic *Roucela* complex (Campanulaceae: campanula) in the eastern Mediterranean. Ecol. Evol. 5, 5329–5343. 10.1002/ece3.1791, PMID: 30151135 PMC6102515

[ref50] D’Amato F. (1949). Triploidia e apomissia in *Statice oleaefolia* Scop. Var. *confusa* Godr. Caryologia 2, 71–84. 10.1080/00087114.1949.10797527

[ref51] Darlington C. D. (1958). The evolution of genetic systems. 2nd Edn. Edinburgh: Oliver & Boyd.

[ref52] Darwin C. (1859). On the origin of species by means of natural selection. London: J Murray.

[ref53] de Vos J. M. Hughes C. E. Schneeweiss G. M. Moore B. R. Conti E. (2014). Heterostyly accelerates diversification via reduced extinction in primroses. Proc. R. Soc. B 281:20140075. 10.1098/rspb.2014.0075, PMID: 24759859 PMC4043087

[ref54] Donoghue M. J. Sanderson M. J. (2015). Confluence, synnovation, and depauperons in plant diversification. New Phytol. 207, 260–274. 10.1111/nph.13367, PMID: 25778694

[ref55] Drummond A. J. Suchard M. A. Xie D. Rambaut A. (2012). Bayesian phylogenetics with BEAUti and the BEAST 1.7. Mol. Biol. Evol. 29, 1969–1973. 10.1093/molbev/mss075, PMID: 22367748 PMC3408070

[ref56] Duggen S. Hoernle K. Van Den Bogaard P. Rüpke L. Morgan J. P. (2003). Deep roots of the Messinian salinity crisis. Nature 422, 602–606. 10.1038/nature01553, PMID: 12686997

[ref57] Dupin J. Matzke N. J. Särkinen T. Knapp S. Olmstead R. G. Bohs L. (2017). Bayesian estimation of the global biogeographical history of the Solanaceae. J. Biogeogr. 44, 887–899. 10.1111/jbi.12898

[ref58] Erben M. (1978). Die Gattung Limonium im südwestmediterranen Raum. Mitt. Bot. Staatssamml. München. 14, 361–631.

[ref59] Erben M. (1979). Karyotype differentiation and its consequences in Mediterranean «*Limonium*». Webbia 34, 409–417. 10.1080/00837792.1979.10670178

[ref60] Faurby S. Eiserhardt W. L. Svenning J. C. (2016). Strong effects of variation in taxonomic opinion on diversification analyses. Methods Ecol. Evol. 7, 4–13. 10.1111/2041-210X.12449

[ref61] Fernández-Mazuecos M. Blanco-Pastor J. L. Juan A. Carnicero P. Forrest A. Alarcón M.. (2019). Macroevolutionary dynamics of nectar spurs, a key evolutionary innovation. New Phytol. 222, 1123–1138. 10.1111/nph.15654, PMID: 30570752

[ref62] Fernández-Palacios J. M. De Nascimento L. Otto R. Delgado J. D. García-del-Rey E. Arévalo J. R. (2011). A reconstruction of Palaeo-Macaronesia, with particular reference to the long-term biogeography of the Atlantic island laurel forests. J. Biogeogr. 38, 226–246. 10.1111/j.1365-2699.2010.02427.x

[ref63] FitzJohn R. G. (2012). Diversitree: comparative phylogenetic analyses of diversification in R. Methods Ecol. Evol. 3, 1084–1092. 10.1111/j.2041-210X.2012.00234.x

[ref64] FitzJohn R. G. Maddison W. P. Otto S. P. (2009). Estimating trait-dependent speciation and extinction rates from incompletely resolved phylogenies. Syst. Biol. 58, 595–611. 10.1093/sysbio/syp067, PMID: 20525612

[ref65] Fiz-Palacios O. Valcárcel V. (2011). Imbalanced diversification of two Mediterranean sister genera (*Bellis* and *Bellium*, Asteraceae) within the same time frame. Plant Syst. Evol. 295, 109–118. 10.1007/s00606-011-0468-5

[ref66] Fiz-Palacios O. Valcárcel V. (2013). From Messinian crisis to Mediterranean climate: a temporal gap of diversification recovered from multiple plant phylogenies. Perspect. Plant Ecol. Evol. Syst. 15, 130–137. 10.1016/j.ppees.2013.02.002

[ref67] Francisco-Ortega J. Jansen R. K. Santos-Guerra A. (1996). Chloroplast DNA evidence of colonization, adaptive radiation, and hybridization in the evolution of the Macaronesian flora. Proc. Natl. Acad. Sci. U. S. A. 93, 4085–4090. 10.1073/pnas.93.9.4085, PMID: 11607675 PMC39491

[ref68] Frodin D. G. (2004). History and concepts of big plant genera. Taxon 53, 753–776. 10.2307/4135449

[ref69] Fuertes-Aguilar J. Ray M. F. Francisco-Ortega J. Santos-Guerra A. Jansen R. K. (2002). Molecular evidence from chloroplast and nuclear markers for multiple colonizations of *Lavatera* (Malvaceae) in the Canary Islands. Syst. Bot. 27, 74–83. 10.1043/0363-6445-27.1.74

[ref70] Gamisch A. (2016). Notes on the statistical power of the binary state speciation and extinction (BiSSE) model. Evol. Bioinforma. 12, 165–174. 10.4137/EBO.S39732, PMID: 27486297 PMC4962954

[ref71] García-Castellanos D. Estrada F. Jiménez-Munt I. Gorini C. Fernández M. Vergés J.. (2009). Catastrophic flood of the Mediterranean after the Messinian salinity crisis. Nature 462, 778–781. 10.1038/nature08555, PMID: 20010684

[ref72] Gargani J. Rigollet C. (2007). Mediterranean Sea level variations during the Messinian salinity crisis. Geophys. Res. Lett. 34:L10405. 10.1029/2007GL029885

[ref73] Georgakopoulou A. Manousou S. Artelari R. Georgiou O. (2006). Breeding systems and cytology in Greek populations of five *Limonium* species (Plumbaginaceae). Willdenowia 36, 741–750. 10.3372/wi.36.36209

[ref74] Goldberg E. E. Igić B. (2012). Tempo and mode in plant breeding system evolution. Evolution 66, 3701–3709. 10.1111/j.1558-5646.2012.01730.x, PMID: 23206129

[ref75] Goldberg E. E. Kohn J. R. Lande R. Robertson K. A. Smith S. A. Igić B. (2010). Species selection maintains self-incompatibility. Science 330, 493–495. 10.1126/science.1194513, PMID: 20966249

[ref76] Goodson B. E. Santos-Guerra A. Jansen R. K. (2006). Molecular systematics of *Descurainia* (Brassicaceae) in the Canary Islands: biogeographic and taxonomic implications. Taxon 55, 671–682. 10.2307/25065643

[ref77] Gorospe J. M. Monjas D. Fernández-Mazuecos M. (2020). Out of the Mediterranean region: worldwide biogeography of snapdragons and relatives (tribe Antirrhineae, Plantaginaceae). J. Biogeogr. 47, 2442–2456. 10.1111/jbi.13939

[ref78] Graham A. (1976). Studies in neotropical paleobotany. II. The Miocene communities of Veracruz, Mexico. Ann. Mo. Bot. Gard. 63, 787–842. 10.2307/2395250

[ref79] Gruenstaeudl M. Carstens B. C. Santos-Guerra A. Jansen R. K. (2017). Statistical hybrid detection and the inference of ancestral distribution areas in *Tolpis* (Asteraceae). Biol. J. Linn. Soc. Lond. 121, 133–149. 10.1093/biolinnean/blw014

[ref80] Hand M. L. Koltunow A. M. (2014). The genetic control of apomixis: asexual seed formation. Genetics 197, 441–450. 10.1534/genetics.114.163105, PMID: 24939990 PMC4063905

[ref81] Haveman R. (2013). Freakish patterns-species and species concepts in apomicts. Nord. J. Bot. 31, 257–269. 10.1111/j.1756-1051.2013.00158.x

[ref82] Hjelmqvist H. Grazi F. (1964). Studies on variation in embryo sac development. Bot. Notiser 117, 141–166.

[ref83] Hodač L. Klatt S. Hojsgaard D. Sharbel T. F. Hörandl E. (2019). A little bit of sex prevents mutation accumulation even in apomictic polyploid plants. BMC Evol. Biol. 19:170. 10.1186/s12862-019-1495-z, PMID: 31412772 PMC6694583

[ref84] Höhna S. May M. R. Moore B. R. (2015). TESS: an R package for efficiently simulating phylogenetic trees and performing Bayesian inference of lineage diversification rates. Bioinformatics 32, 789–791. 10.1093/bioinformatics/btv651, PMID: 26543171

[ref85] Hojsgaard D. Hörandl E. (2015). A little bit of sex matters for genome evolution in asexual plants. Front. Plant Sci. 6:82. 10.3389/fpls.2015.00082, PMID: 25750646 PMC4335465

[ref600] Holsinger K. E. (2000). Reproductive systems and evolution in vascular plants. Proc. Natl. Acad. Sci. U. S. A. 97, 7037–7042. 10.1073/pnas.97.13.7037 10860968 PMC34381

[ref700] Hojsgaard D. Klatt S. Baier R. Carman J. G. Hörandl E. (2014). Taxonomy and biogeography of apomixis in angiosperms and associated biodiversity characteristics. Crit. Rev. Plant Sci. 33, 414–427. 10.1080/07352689.2014.898488, PMID: 27019547 PMC4786830

[ref86] Hörandl E. Grossniklaus U. van Dijk P. J. Sharbel T. (2007). Apomixis: Evolution, mechanisms and perspectives, Regnum Vegetabile Vol. 147 Rugell, Liechtenstein: Gantner Verlag.

[ref87] Hörandl E. Hojsgaard D. (2012). The evolution of apomixis in angiosperms: a reappraisal. Plant Biosyst. 146, 681–693. 10.1080/11263504.2012.716795

[ref88] Howard C. C. Landis J. B. Beaulieu J. M. Cellinese N. (2020). Geophytism in monocots leads to higher rates of diversification. New Phytol. 225, 1023–1032. 10.1111/nph.16155, PMID: 31469440

[ref89] Hsü K. J. Ryan W. B. Cita M. B. (1973). Late Miocene desiccation of the Mediterranean. Nature 242, 240–244. 10.1038/242240a0

[ref90] Ingrouille M. J. (1984). A taxometric analysis of *Limonium* (Plumbaginaceae) in Western Europe. Plant Syst. Evol. 147, 103–118. 10.1007/BF00984583

[ref91] Jaén-Molina R. Marrero-Rodríguez Á. Caujapé-Castells J. Ojeda D. I. (2020). Molecular phylogenetics of *Lotus* (Leguminosae) with emphasis in the tempo and patterns of colonization in the Macaronesian region. Mol. Phylogenet. Evol. 154:106970. 10.1016/j.ympev.2020.106970, PMID: 33031929

[ref92] Jiménez A. Weigelt B. Santos-Guerra A. Caujapé-Castells J. Fernández-Palacios J. M. Conti E. (2017). Surviving in isolation: genetic variation, bottlenecks and reproductive strategies in the Canarian endemic *Limonium macrophyllum* (Plumbaginaceae). Genetica 145, 91–104. 10.1007/s10709-017-9948-z, PMID: 28108874

[ref93] Jiménez-Moreno G. Fauquette S. Suc J. P. (2010). Miocene to Pliocene vegetation reconstruction and climate estimates in the Iberian Peninsula from pollen data. Rev. Palaeobot. Palynol. 162, 403–415. 10.1016/j.revpalbo.2009.08.001

[ref94] Khan Z. Santpere G. Traveset A. (2012). Breeding system and ecological traits of the critically endangered endemic plant *Limonium barceloi* (Gil and Llorens) (Plumbaginaceae). Plant Syst. Evol. 298, 1101–1110. 10.1007/s00606-012-0619-3

[ref95] Kim S. C. McGowen M. R. Lubinsky P. Barber J. C. Mort M. E. Santos-Guerra A. (2008). Timing and tempo of early and successive adaptive radiations in Macaronesia. PLoS One 3:e2139. 10.1371/journal.pone.0002139 18478126 PMC2367450

[ref96] Kodandaramaiah U. Murali G. (2018). What affects power to estimate speciation rate shifts? PeerJ 6:e5495. 10.7717/peerj.5495, PMID: 30155369 PMC6108317

[ref97] Kool A. Thulin M. (2017). A giant spurrey on a tiny island: on the phylogenetic position of *Sanctambrosia manicata* (Caryophyllaceae) and the generic circumscriptions of *Spergula*, *Spergularia* and *Rhodalsine*. Taxon 66, 615–622. 10.12705/663.6

[ref98] Koutroumpa K. Theodoridis S. Warren B. H. Jiménez A. Celep F. Doğan M.. (2018). An expanded molecular phylogeny of Plumbaginaceae, with emphasis on *Limonium* (sea lavenders): taxonomic implications and biogeographic considerations. Ecol. Evol. 8, 12397–12424. 10.1002/ece3.4553, PMID: 30619554 PMC6308857

[ref550] Krijgsman W. Hilgen F. J. Raffi I. Sierro F. J. Wilson D. S. (1999). Chronology, causes and progression of the Messinian salinity crisis. Nature 400, 652–655. 10.1038/23231

[ref100] Kunkel G. (2012). Biogeography and ecology in the Canary Islands. The Hague: Springer Science & Business Media.

[ref101] Lagomarsino L. P. Condamine F. L. Antonelli A. Mulch A. Davis C. C. (2016). The abiotic and biotic drivers of rapid diversification in Andean bellflowers (Campanulaceae). New Phytol. 210, 1430–1442. 10.1111/nph.13920, PMID: 26990796 PMC4950005

[ref102] Landis M. J. Matzke N. J. Moore B. R. Huelsenbeck J. P. (2013). Bayesian analysis of biogeography when the number of areas is large. Syst. Biol. 62, 789–804. 10.1093/sysbio/syt040, PMID: 23736102 PMC4064008

[ref103] Lens F. Davin N. Smets E. del Arco M. (2013). Insular woodiness on the Canary Islands: a remarkable case of convergent evolution. Int. J. Plant Sci. 174, 992–1013. 10.1086/670259

[ref104] Letsch H. Gottsberger B. Metzl C. Astrin J. Friedman A. L. McKenna D. D.. (2018). Climate and host-plant associations shaped the evolution of ceutorhynch weevils throughout the Cenozoic. Evolution 72, 1815–1828. 10.1111/evo.13520, PMID: 30040114 PMC6175111

[ref105] Lisiecki L. E. Raymo M. E. (2007). Plio-Pleistocene climate evolution: trends and transitions in glacial cycle dynamics. Quat. Sci. Rev. 26, 56–69. 10.1016/j.quascirev.2006.09.005

[ref106] Lledó M. D. Crespo M. B. Fay M. F. Chase M. W. (2005). Molecular phylogenetics of *Limonium* and related genera (Plumbaginaceae): biogeographical and systematic implications. Am. J. Bot. 92, 1189–1198. 10.3732/ajb.92.7.1189, PMID: 21646141

[ref107] Lledó M. D. Karis P. O. Crespo M. B. Fay M. F. Chase M. W. (2011). “Endemism and evolution in macaronesian and mediterranean Limonium taxa” in The biology of island floras. eds. Bramwell D. Caujapé-Castells J. (Cambridge: Cambridge University Press), 325–337.

[ref108] Mabberley D. J. (2017). Mabberley's plant-book: A portable dictionary of plants, their classification and uses. 4th Edn. Cambridge: Cambridge University Press.

[ref109] MacArthur R. H. Wilson E. O. (1967). Island biogeography. Princeton: Princeton University Press.

[ref110] Macphail M. K. (1999). Palynostratigraphy of the Murray Basin, inland southeastern Australia. Palynology 23, 197–240. 10.1080/01916122.1999.9989528

[ref111] Maddison W. P. FitzJohn R. G. (2015). The unsolved challenge to phylogenetic correlation tests for categorical characters. Syst. Biol. 64, 127–136. 10.1093/sysbio/syu070, PMID: 25209222

[ref112] Maddison W. P. Midford P. E. Otto S. P. (2007). Estimating a binary character's effect on speciation and extinction. Syst. Biol. 56, 701–710. 10.1080/10635150701607033, PMID: 17849325

[ref113] Magallón S. Gómez-Acevedo S. Sánchez-Reyes L. L. Hernández-Hernández T. (2015). A metacalibrated time-tree documents the early rise of flowering plant phylogenetic diversity. New Phytol. 207, 437–453. 10.1111/nph.13264, PMID: 25615647

[ref114] Magallon S. Sanderson M. J. (2001). Absolute diversification rates in angiosperm clades. Evolution 55, 1762–1780. 10.1111/j.0014-3820.2001.tb00826.x, PMID: 11681732

[ref115] Majeský Ľ. Krahulec F. Vašut R. J. (2017). How apomictic taxa are treated in current taxonomy: a review. Taxon 66, 1017–1040. 10.12705/665.3

[ref116] Malekmohammadi M. Akhani H. Borsch T. (2017). Phylogenetic relationships of *Limonium* (Plumbaginaceae) inferred from multiple chloroplast and nuclear loci. Taxon 66, 1128–1146. 10.12705/665.8

[ref117] Manafzadeh S. Salvo G. Conti E. (2014). A tale of migrations from east to west: the Irano-Turanian floristic region as a source of Mediterranean xerophytes. J. Biogeogr. 41, 366–379. 10.1111/jbi.12185

[ref118] Mansion G. Rosenbaum G. Schoenenberger N. Bacchetta G. Rosselló J. A. Conti E. (2008). Phylogenetic analysis informed by geological history supports multiple, sequential invasions of the Mediterranean Basin by the angiosperm family Araceae. Syst. Biol. 57, 269–285. 10.1080/10635150802044029, PMID: 18425714

[ref119] Mansion G. Selvi F. Guggisberg A. Conti E. (2009). Origin of Mediterranean insular endemics in the Boraginales: integrative evidence from molecular dating and ancestral area reconstruction. J. Biogeogr. 36, 1282–1296. 10.1111/j.1365-2699.2009.02082.x

[ref120] Marrero Á. Almeida R. (2003). Novedades taxonómicas del género *Limonium* mill. Subsecc. Nobiles en gran Canaria (islas Canarias) (Plumbaginaceae-Staticoideae). Vieraea 31, 391–406.

[ref121] Matzke N. J. (2013). Probabilistic historical biogeography: new models for founder-event speciation, imperfect detection, and fossils allow improved accuracy and model-testing. Front. Biogeogr. 5, 242–248. 10.21425/F5FBG19694

[ref122] Matzke N. J. (2016). “Stochastic mapping under biogeographical models.” PhyloWiki BioGeoBEARS website, 2016. Available at: http://phylo.wikidot.com/biogeobears#stochastic_mapping (Accessed September 15, 2020).

[ref123] Mayhew P. J. Jenkins G. B. Benton T. G. (2008). A long-term association between global temperature and biodiversity, origination and extinction in the fossil record. Proc. R. Soc. B 275, 47–53. 10.1098/rspb.2007.1302, PMID: 17956842 PMC2562410

[ref124] Medail F. Quezel P. (1997). Hot-spots analysis for conservation of plant biodiversity in the Mediterranean Basin. Ann. Mo. Bot. Gard. 84, 112–127. 10.2307/2399957

[ref125] Mesa R. Santos A. Oval J. P. Voggenreiter V. (2001). *Limonium relicticum*, una nueva especie Para La Gomera, islas Canarias (Plumbaginaceae). Vieraea 29, 111–118.

[ref126] Miller K. G. Kominz M. A. Browning J. V. Wright J. D. Mountain G. S. Katz M. E.. (2005). The Phanerozoic record of global sea-level change. Science 310, 1293–1298. 10.1126/science.1116412, PMID: 16311326

[ref127] Miller M. A. Pfeiffer W. Schwartz T. (2010). “Creating the CIPRES Science Gateway for inference of large phylogenetic trees” in Proceedings of the Gateway Computing Environments Workshop (GCE), Nov 14, 2010, New Orleans, LA, 1–8.

[ref128] Mittermeier R. A. Turner W. R. Larsen F. W. Brooks T. M. Gascon C. (2011). “Global biodiversity conservation: the critical role of hotspots” in Biodiversity hotspots. eds. Zachos F. E. Habel J. C. (Berlin, Heidelberg: Springer), 3–22.

[ref129] Moore M. J. Francisco-Ortega J. Santos-Guerra A. Jansen R. K. (2002). Chloroplast DNA evidence for the roles of island colonization and extinction in *Tolpis* (Asteraceae: Lactuceae). Am. J. Bot. 89, 518–526. 10.3732/ajb.89.3.518, PMID: 21665651

[ref130] Morlon H. Lewitus E. Condamine F. L. Manceau M. Clavel J. Drury J. (2016). RPANDA: an R package for macroevolutionary analyses on phylogenetic trees. Methods Ecol. Evol. 7, 589–597. 10.1111/2041-210X.12526

[ref131] Morlon H. Parsons T. L. Plotkin J. B. (2011). Reconciling molecular phylogenies with the fossil record. Proc. Natl. Acad. Sci. U. S. A. 108, 16327–16332. 10.1073/pnas.1102543108, PMID: 21930899 PMC3182748

[ref132] Morlon H. Potts M. D. Plotkin J. B. (2010). Inferring the dynamics of diversification: a coalescent approach. PLoS Biol. 8:e1000493. 10.1371/journal.pbio.1000493, PMID: 20927410 PMC2946937

[ref133] Mort M. E. Soltis D. E. Soltis P. S. Francisco-Ortega J. Santos-Guerra A. (2002). Phylogenetics and evolution of the Macaronesian clade of Crassulaceae inferred from nuclear and chloroplast sequence data. Syst. Bot. 27, 271–288. 10.1043/0363-6445-27.2.271

[ref134] Muller J. (1981). Fossil pollen records of extant angiosperms. Bot. Rev. 47, 1–140.

[ref135] Myers N. Mittermeier R. A. Mittermeier C. G. Da Fonseca G. A. Kent J. (2000). Biodiversity hotspots for conservation priorities. Nature 403, 853–858. 10.1038/35002501, PMID: 10706275

[ref136] Navarro-Pérez M. L. Vargas P. Fernández-Mazuecos M. López J. Valtueña F. J. Ortega-Olivencia A. (2015). Multiple windows of colonization to Macaronesia by the dispersal-unspecialized *Scrophularia* since the late Miocene. Perspect. Plant Ecol. Evol. Syst. 17, 263–273. 10.1016/j.ppees.2015.05.002

[ref137] Nieto Feliner G. N. (2014). Patterns and processes in plant phylogeography in the Mediterranean Basin. A review. Perspect. Plant Ecol. 16, 265–278. 10.1016/j.ppees.2014.07.002

[ref138] Nürk N. M. Atchison G. W. Hughes C. E. (2019). Island woodiness underpins accelerated disparification in plant radiations. New Phytol. 224, 518–531. 10.1111/nph.15797, PMID: 30883788 PMC6766886

[ref139] Palacios C. González-Candelas F. (1997). Lack of genetic variability in the rare and endangered *Limonium cavanillesii* (Plumbaginaceae) using RAPD markers. Mol. Ecol. 6, 671–675. 10.1046/j.1365-294X.1997.00232.x 9421917

[ref140] Palacios C. Kresovich S. González-Candelas F. (1999). A population genetic study of the endangered plant species *Limonium dufourii* (Plumbaginaceae) based on amplified fragment length polymorphism (AFLP). Mol. Ecol. 8, 645–657. 10.1046/j.1365-294X.1999.t01-1-00597.x

[ref141] Palacios C. Rosselló J. A. González-Candelas F. (2000). Study of the evolutionary relationships among *Limonium* species (Plumbaginaceae) using nuclear and cytoplasmic molecular markers. Mol. Phylogenet. Evol. 14, 232–249. 10.1006/mpev.1999.0690, PMID: 10679157

[ref800] Plummer M. Best N. Cowles K. Vines K. (2006). CODA: convergence diagnosis and output analysis for MCMC. R news 6, 7–11.

[ref142] Pound M. J. Salzmann U. (2017). Heterogeneity in global vegetation and terrestrial climate change during the late Eocene to early Oligocene transition. Sci. Rep. 7:43386. 10.1038/srep43386, PMID: 28233862 PMC5324063

[ref143] Pyron R. A. Burbrink F. T. (2013). Phylogenetic estimates of speciation and extinction rates for testing ecological and evolutionary hypotheses. Trends Ecol. Evol. 28, 729–736. 10.1016/j.tree.2013.09.007, PMID: 24120478

[ref145] Rabosky D. L. Goldberg E. E. (2015). Model inadequacy and mistaken inferences of trait-dependent speciation. Syst. Biol. 64, 340–355. 10.1093/sysbio/syu131, PMID: 25601943

[ref146] Rabosky D. L. Goldberg E. E. (2017). FiSSE: a simple nonparametric test for the effects of a binary character on lineage diversification rates. Evolution 71, 1432–1442. 10.1111/evo.13227, PMID: 28316067

[ref147] Rabosky D. L. Grundler M. Anderson C. Title P. Shi J. J. Brown J. W. (2014). BAMM tools: an R package for the analysis of evolutionary dynamics on phylogenetic trees. Methods Ecol. Evol. 5, 701–707. 10.1111/2041-210X.12199

[ref148] Rambaut A. Drummond A. J. Xie D. Baele G. Suchard M. A. (2018). Posterior summarization in Bayesian phylogenetics using tracer 1.7. Syst. Biol. 67, 901–904. 10.1093/sysbio/syy032, PMID: 29718447 PMC6101584

[ref144] R Core Team (2018). R: a language and environment for statistical computing. R foundation for statistical computing, Vienna, Austria. Available at: https://www.R-project.org/ (Accessed September 15, 2020).

[ref149] Ree R. H. Sanmartín I. (2018). Conceptual and statistical problems with the DEC+ J model of founder-event speciation and its comparison with DEC via model selection. J. Biogeogr. 45, 741–749. 10.1111/jbi.13173

[ref150] Ree R. H. Smith S. A. (2008). Maximum likelihood inference of geographic range evolution by dispersal, local extinction, and cladogenesis. Syst. Biol. 57, 4–14. 10.1080/10635150701883881, PMID: 18253896

[ref151] Renner S. (2004). Plant dispersal across the tropical Atlantic by wind and sea currents. Int. J. Plant Sci. 165, S23–S33. 10.1086/383334

[ref152] Rieseberg L. H. Raymond O. Rosenthal D. M. Lai Z. Livingstone K. Nakazato T.. (2003). Major ecological transitions in wild sunflowers facilitated by hybridization. Science 301, 1211–1216. 10.1126/science.1086949, PMID: 12907807

[ref153] Rodríguez-Sánchez F. Pérez-Barrales R. Ojeda F. Vargas P. Arroyo J. (2008). The strait of Gibraltar as a melting pot for plant biodiversity. Quat. Sci. Rev. 27, 2100–2117. 10.1016/j.quascirev.2008.08.006

[ref154] Rögl F. (1999). Mediterranean and paratethys. Facts and hypotheses of an Oligocene to Miocene paleogeography (short overview). Geol. Carpath. 50, 339–349.

[ref155] Rohling E. J. Foster G. L. Grant K. M. Marino G. Roberts A. P. Tamisiea M. E.. (2014). Sea-level and deep-sea-temperature variability over the past 5.3 million years. Nature 508, 477–482. 10.1038/nature13230, PMID: 24739960

[ref156] Róis A. S. Sádio F. Paulo O. S. Teixeira G. Paes A. P. Espírito-Santo D.. (2016). Phylogeography and modes of reproduction in diploid and tetraploid halophytes of *Limonium* species (Plumbaginaceae): evidence for a pattern of geographical parthenogenesis. Ann. Bot. 117, 37–50. 10.1093/aob/mcv138, PMID: 26424783 PMC4701142

[ref157] Romeiras M. M. Monteiro F. Duarte M. C. Schaefer H. Carine M. (2015). Patterns of genetic diversity in three plant lineages endemic to the Cape Verde Islands. AoB Plants 7, 1–11. 10.1093/aobpla/plv051, PMID: 25979965 PMC4501515

[ref158] Romeiras M. M. Vieira A. Silva D. N. Moura M. Santos-Guerra A. Batista D.. (2016). Evolutionary and biogeographic insights on the Macaronesian *Beta*-*Patellifolia* species (Amaranthaceae) from a time-scaled molecular phylogeny. PLoS One 11:e0152456. 10.1371/journal.pone.0152456, PMID: 27031338 PMC4816301

[ref159] Ronquist F. (1997). Dispersal-vicariance analysis: a new approach to the quantification of historical biogeography. Syst. Biol. 46, 195–203. 10.1093/sysbio/46.1.195

[ref160] Ronquist F. Teslenko M. Van Der Mark P. Ayres D. L. Darling A. Höhna L.. (2012). MrBayes 3.2: efficient Bayesian phylogenetic inference and model choice across a large model space. Syst. Biol. 61, 539–542. 10.1093/sysbio/sys029, PMID: 22357727 PMC3329765

[ref161] Rosenbaum G. Lister G. S. Duboz C. (2002). Relative motions of Africa, Iberia and Europe during alpine orogeny. Tectonophysics 359, 117–129. 10.1016/S0040-1951(02)00442-0

[ref162] Rutschmann F. Eriksson T. Salim K. A. Conti E. (2007). Assessing calibration uncertainty in molecular dating: the assignment of fossils to alternative calibration points. Syst. Biol. 56, 591–608. 10.1080/10635150701491156, PMID: 17654364

[ref163] Salvo G. Ho S. Y. Rosenbaum G. Ree R. Conti E. (2010). Tracing the temporal and spatial origins of island endemics in the Mediterranean region: a case study from the citrus family (*Ruta* L., Rutaceae). Syst. Biol. 59, 705–722. 10.1093/sysbio/syq046, PMID: 20841320

[ref164] Sanmartín I. Van Der Mark P. Ronquist F. (2008). Inferring dispersal: a Bayesian approach to phylogeny-based island biogeography, with special reference to the Canary Islands. J. Biogeogr. 35, 428–449. 10.1111/j.1365-2699.2008.01885.x

[ref165] Schenk J. J. (2016). Consequences of secondary calibrations on divergence time estimates. PLoS One 11:e0148228. 10.1371/journal.pone.0148228, PMID: 26824760 PMC4732660

[ref166] Sharma R. Bhat V. (2020). “Role of Apomixis in Perpetuation of Flowering Plants: Ecological Perspective” in Reproductive Ecology of Flowering Plants: Patterns and Processes. eds. Tandon R. Shivanna K. Koul M. (Singapore: Springer), 275–297.

[ref167] Soltis P. S. Folk R. A. Soltis D. E. (2019). Darwin review: angiosperm phylogeny and evolutionary radiations. Proc. R. Soc. B 286:20190099. 10.1098/rspb.2019.0099

[ref168] Soltis D. E. Mort M. E. Latvis M. Mavrodiev E. V. O'Meara B. C. Soltis P. S.. (2013). Phylogenetic relationships and character evolution analysis of Saxifragales using a supermatrix approach. Am. J. Bot. 100, 916–929. 10.3732/ajb.1300044, PMID: 23629845

[ref169] Stamatakis A. (2014). RAxML version 8: a tool for phylogenetic analysis and post-analysis of large phylogenies. Bioinformatics 30, 1312–1313. 10.1093/bioinformatics/btu033, PMID: 24451623 PMC3998144

[ref170] Suc J. P. (1984). Origin and evolution of the Mediterranean vegetation and climate in Europe. Nature 307, 429–432. 10.1038/307429a0

[ref171] Thompson J. D. (2005). Plant evolution in the Mediterranean. New York: Oxford University Press.

[ref172] Triantis K. A. Borges P. A. Hortal J. Whittaker R. J. (2010). “The Macaronesian province: patterns of species richness and endemism of arthropods” in Terrestrial arthropods of Macaronesia-biodiversity, ecology and evolution. eds. Serrano A. R. M. Borges P. A. V. Boieiro M. Oromí P. (Lisboa: Sociedade Portuguesa de Entomologia), 49–71.

[ref173] Valente L. M. Savolainen V. Vargas P. (2010). Unparalleled rates of species diversification in Europe. Proc. R. Soc. B 277, 1489–1496. 10.1098/rspb.2009.2163, PMID: 20106850 PMC2871840

[ref174] Van Campo É. (1976). La flore sporopollinique du gisement Miocène terminal de Venta del Moro. dissertation. Montpellier: Université Montpellier II Sciences et Techniques du Languedoc.

[ref175] Van Hinsbergen D. J. Torsvik T. H. Schmid S. M. Maţenco L. C. Maffione M. Vissers R. L. (2020). Orogenic architecture of the Mediterranean region and kinematic reconstruction of its tectonic evolution since the Triassic. Gondwana Res. 81, 79–229. 10.1016/j.gr.2019.07.009

[ref176] Vargas P. Fernández-Mazuecos M. Heleno R. (2018). Phylogenetic evidence for a Miocene origin of Mediterranean lineages: species diversity, reproductive traits and geographical isolation. Plant Biol. 20, 157–165. 10.1111/plb.12626, PMID: 28892240

[ref177] Wallace A. R. (1878). Tropical nature and other essays. London: MacMillan & Co.

[ref178] Warren B. H. Hagen O. Gerber F. Thébaud C. Paradis E. Conti E. (2018). Evaluating alternative explanations for an association of extinction risk and evolutionary uniqueness in multiple insular lineages. Evolution 72, 2005–2024. 10.1111/evo.13582, PMID: 30151822

[ref179] Warren B. H. Simberloff D. Ricklefs R. E. Aguilée R. Condamine F. L. Gravel D.. (2015). Islands as model systems in ecology and evolution: prospects fifty years after MacArthur-Wilson. Ecol. Lett. 18, 200–217. 10.1111/ele.12398, PMID: 25560682

[ref180] Zachos J. C. Dickens G. R. Zeebe R. E. (2008). An early Cenozoic perspective on greenhouse warming and carbon-cycle dynamics. Nature 451, 279–283. 10.1038/nature065, PMID: 18202643

