# Supplementary material for: Geo-Climatic Changes and Apomixis as Major Drivers of Diversification in the Mediterranean Sea Lavenders (Limonium Mill.)
Source: Front Plant Sci. 2021 Jan 12;11:612258. doi: 10.3389/fpls.2020.612258 (PMC7835328; doi:10.3389/fpls.2020.612258)
Supplement: Supplementary file 1 [file Data_Sheet_1.pdf]

frontiers

Supplementary Material

Contents

**Table S1** Taxon sampling including GenBank accession numbers and voucher information.	3
**Table S2** Divergence time estimates for major clades in the 'Supermatrix-ITS-like', 'Supermatrix-cpDNA-like', 'Supermatrix', ITS and cpDNA MCC trees.	22
**Table S3** Species distributions and assignments of species into the nine major areas for biogeographic analyses.	23
**Table S4** Time-stratified dispersal multiplier matrices used in biogeographic analyses.	37
**Table S5** Sampling fractions per section/clade of *Limonium* for the two MCC trees used in BAMM analyses.	38
**Table S6** *Limonium* species with their ploidy level, chromosome number in parenthesis, and their reproductive strategy.	38
**Table S7** Biogeographic models tested in this study, along with parameter estimates, log-likelihoods, AIC and AICc comparisons.	42
**Table S8** Number of dispersal events averaged across 200 Biogeographical Stochastic Maps (BSMs) with standard deviations in parentheses for analyses of the two MCC trees.	43
**Table S9** Biogeographical models tested for analyses of *Jovibarba-Ctenostachys* clade, along with parameter estimates, log-likelihoods, AIC and AICc comparisons.	44
**Table S10** Time and paleoenvironment-dependent diversification models fitted to the entire or parts of *Limonium* tree (RPANDA analyses) for the two MCC trees.	45
**Table S11** The fit of alternative BiSSE models used to test the effect of breeding systems in diversification rates for *Limonium* under three sampling scenarios for analyses of the two MCC trees.	50
**Table S12** The fit of alternative HiSSE models used to test the effect of breeding systems in diversification rates for *Limonium* under three sampling scenarios for analyses of the two MCC trees.	52
**Table S13** The fit of alternative GeoHiSSE models used to test the effect of Euro-Mediterranean range in diversification rates for *Limonium* for analyses of the two MCC trees.	54
**Table S14** The fit of alternative BiSSE models used to test the effect of woodiness in diversification rates for *L.* subg. *Pteroclados s.l.* clade in the two MCC trees.	55
**Table S15** The fit of alternative HiSSE models used to test the effect of woodiness in diversification rates for *L.* subg. *Pteroclados s.l.* clade in the two MCC trees.	56

Figure S1 Time calibrated phylogeny based on 'Supermatrix-ITS-like' dataset (MCC tree), with median ages on nodes and 95% HPD interval bars. 57

Figure S2 Time calibrated phylogeny based on 'Supermatrix-cpDNA-like' dataset (MCC tree), with median ages on nodes and 95% HPD interval bars. 58

Figure S3 Environmental variables through time and their curves used in RPANDA paleo-environment dependent diversification analyses. 59

Figure S4 Ancestral range estimates for 'Supermatrix-ITS-like' MCC tree. 60

Figure S5 Ancestral range estimates for 'Supermatrix-cpDNA-like' MCC tree. 62

Figure S6 Bar plots of prior and posterior distributions of the number of shifts from BAMM analyses for the two MCC trees. 64

Figure S7 The 95% credible set of shift configurations from BAMM analyses of the two MCC trees. 65

Figure S8 Results on episodic shifts of speciation and extinction rates, and mass extinction events from TESS analyses for the two MCC trees. 67

Supplementary References 71

Table S1 Taxon sampling including GenBank accession numbers and voucher information. For *Limonium,* we additionally include the collecting locality information of the samples. Newly generated sequences are marked with an asterisk.

Limonium species	Voucher	*trnL-F*	*rbcL*	*matK*	ITS
L. aegaeum **Erben & Brullo**	K. Koutroumpa 13 (Z): Greece, Crete, Malia-Agio Pneuma	MW289857*	MW289894*	MW289848*	-
L. albomarginatum **Brullo**	Artelari & Georgiou 1738 (UPA): Greece, Peloponnese, Gerolimenas	MH561056	MH582667	MH582899	MH582550
L. algarvense **Erben**	M.M. Romeiras 401 (LISC): Portugal, Algarve, National Park of Ria Formosa	MH561040	MH582715	MH582955	-
L. ammophilon **(Papatsou & Phitos) Domina**	K. Koutroumpa 781 (Z): Greece, Karpathos, Damatria	MH561021	MH582784	MH582855	MH582564
L. amopicum **Erben & Brullo**	K. Koutroumpa 796 (Z): Greece, Karpathos, Finiki	MH561029	MH582763	MH582876	MH582571
L. anatolicum **Hedge**	F. Celep 3924 (GAZI): Turkey, Ankara, Şereflikoçhisar; Akhani et al. 17999 (Hb. Akh.): Turkey, Aksaray	MH560985	MH582745	MH582839	JX983724
L. anthericoides **(Schltr.) R.A.Dyer**	E. Esterhuysen 36001 [E00770638] (E): South Africa, Western Cape, Bredassdorp	MH561136	-	-	MH582626
L. aphroditae **R.Artelari & Georgiou**	K. Koutroumpa 880 (Z): Greece, Kythira, Limnaria	MH561057	MH582669	MH582903	MH582574
L. aragonense **(Debeaux ex Willk.) Pignatti**	ipse 72/123 E [ZT-00077028] (ZT): Spain, Prov. Teruel, Villel	MH561086	MH582725	MH582908	MH582615
L. arboreum **(Willd.) Erben, A.Santos & Reyes-Bet.**	A. Jiménez AJ279-1 (Z): Spain, Canary Islands, Tenerife, Los Silos	MH561121	MW289898*	MH583006	MH582643
L. archaeothirae **Erben & Brullo**	Thomas Koutroumpas 942 (Z): Greece, Santorini, Kamari	MH561020	MH582764	MH582864	MH582592
L. articulatum **(Loisel.) Kuntze**	K. Koutroumpa 1001 (Z): Corse, Ficaghiola	MH561084	MH582670	MH582909	MH582579
L. asparagoides **(Batt.) Maire**	J. Lambinon 94/307 & G. Van Den Sande [P05091481] (P): Morocco, Kariet Arkmane	MH561035	-	-	MH582620
L. aucheri **(Girard) Greuter & Burdet**	E. Cabi & F. Celep 3901 (GAZI): Turkey, Muğla, Datça, Knidos	MH561034	MH582787	MH582865	MH582576
L. aureum **(L.) Hill ex Kuntze**	Sino-American-British Yushu Expedition (1996) 1901 [E00059615] (E): China, Qinghai Province, Chindu Xian, Xiwu Xiang; LiuJQ-08KLS-160	MH561103	JN187124	MH582970	JN187115

***L. auriculae-ursifolium* (Pourr.) Druce**	K. Koutroumpa 1003 (Z): France, Loire-Atlantique, La Turballe	MH561077	MH582699	MH582907	MH582605
***L. australe* (R. Br.) Kuntze**	V. Stajsic & J.G. Eichler 7008 [MEL 2379780] (MEL): Australia, Victoria, Jam Jerrup; Yonekura K., S. Nishiro & K. Tanak 99334 (TUS): Australia, New South Wales	MF083851	-	-	AB190860/ AB190861
***L. avei* (De Not.) Brullo & Erben**	R. Hand 4685 [B 100456127] (B): Cyprus, Larnaka	MF083878	-	MF190404	MF041895
***L. axillare* (Forssk.) Kuntze**	M. Malekmohammadi & al. 3974 (Hb. Akh.): Iran, Bushehr, Bidkhoon, Nayband National Park; Knees, Sabina Georgina 9036 (collected with R. Richer & Fran Gillespie) [E00647681] (E): Qatar, Madinat Ash Shamal, Umm Tais	MF083920	MW289893*	MH582887	JX983660
***L. beaumierianum* (Coss. ex Maire) Maire**	Kew DNA bank 1932	MH561139	MH582808	MH582993	MH582627
***L. bellidifolium* (Gouan) Dumort.**	E. Cabi & F. Celep 3912 (GAZI): Turkey, İzmir, Çiğli, Homa Dalyanı	MH561108	MH582789	MH582978	MH582518
***L. benmageci* Marrero Rodr.**	A. Jiménez AJben1: Spain, Canary islands, Gran Canaria, Punta de La Aldea	MH561122	MH582801	MH583004	-
***L. biflorum* (Pignatti) Pignatti**	M. Luceño & J. Pedrol 1618 JP [Z-000102463] (Z): Spain, Baleares, Formentera, Estany Pudent, Les Salines; Miquel A. Conesa 2009-46	MW289867*	KJ608050	MH582961	-
***L. binervosum* (G.E.Sm.) C.E.Salmon**	N. Nydegger 36863 [P05091530] (P): Portugal, Estremadura, N São Pedro de Moel	KP159566	MW289879*	-	MH582607
***L. bocconei* (Lojac.) Litard.**	Jürg Röthlisberger 7/8/1998 [Z-000102464] (Z): Italy, Sicilia, Isole Egadi	MH561076	MH582671	MH582910	MH582616
***L. bollei* (Webb ex Wangerin) Erben**	A. Jiménez, A. Santos-Guerra AJ256-1 (Z): Spain, Canary Islands, Lobos islet, Las Lagunillas	MH561036	MH582716	MH582956	-
***L. bonduellei* (T.Lestib.) Kuntze**	D.Podlech 52610 [P05091477] (P): Morocco, 5km NW de Tazenakht sur la route d'Ourzazate	MH561133	MH582809	MH582994	MH582628
***L. bonifaciense* Arrigoni & Diana**	K. Koutroumpa 1002 (Z): Corse, Bonifacio, Port Stagad	MH561058	MH582698	MH582911	MH582580
***L. bourgeaui* (Webb ex Webb) Kuntze**	A. Jiménez AJ223-1 (Z): Spain, Canary Islands, Lanzarote, Caleta de Famara	MH561128	MH582812	MH582995	MH582630

	TMP 13193 [L.2643266] (L): Argentina, Salina Chica; M. Weigend & al. 5941 [B 10 0066482]				
***L. brasiliense* (Boiss.) Kuntze**	(B): Argentina, Chubut	MH560981	MH582736	MF190434	MF041922
***L. brassicifolium* (Webb & Berthel.) Kuntze**	A. Santos-Guerra AS 3443-11 (ORT): Spain, Canary islands, Riscos de Juel, La Gomera	MH561130	MH582802	MH582999	MH582632
***L. braunii* (Bolle) A.Chev.**	MC. Duarte 3526 (LISC): Cape Verde, Santo Antão Island	MH560998	MH582752	MH582891	MH582526
***L. brevipetiolatum* R.Artelari & Erben**	K. Koutroumpa 943 (Z): Greece, Kefalonia, Argostoli, Fanari	MH560970	MH582731	MH582840	MH582544
***L. brunneri* (Webb. ex Boiss.) Kuntze**	C. Fernandes & M.M. Romeiras s.n. (LISC): Cape Verde, Sal Island	MH560999	MH582748	MH582890	MH582527
***L. caesium* (Girard) Kuntze**	Serra & Crespo s.n. (K)	MW289858*	MH582765	MH582879	MH582623
***L. californicum* (Boiss.) A.Heller**	Richard R. Halse 6313 [P05117997] (P): North America, Waldport, Oregon, Lint Slough	MH560983	MH582739	MH582841	MH582539
***L. calliopsium* Alf.Mayer**	K. Koutroumpa 668 (Z): Crete, Rethymno, Petre	MH561011	MH582766	MH582866	MH582552
***L. camposanum* M. Erben**	Miquel A. Conesa 2009-36: Spain, Mallorca; Palacios et al. JAR-95111: Spain, Mallorca, Cala Pi	-	KJ608041	-	AJ222841
***L. cancellatum* (Bertol.) Kuntze**	K. Koutroumpa 1004 (Z): Croatia, Island of Vis south of Rukavac village	MH561090	MH582672	MH582944	MH582604
***L. capense* (L.Bolus) L.Bolus**	Peter Goldblatt & J.C. Manning 10434 [WAG.1169163] (WAG): South Africa, Western Cape, Clanwilliam	MH560967	MH582727	-	MW290499*
***L. carnosum* (Boiss.) Kuntze**	H. Akhani & al. 18997 (Hb. Akh.): Iran, Azerbaijan, Khoy	MF083922	-	MF190451	JX983726
***L. carolinianum* (Walter) Britton**	K. Koutroumpa 1005 (Z): Canada, Nova Scotia, Colchester Co.; Abbott 24357 (FLAS)	MH560984	KJ773631	KJ772894	MH582547
***L. carpathum* (Rech.f.) Rech.f.**	K. Koutroumpa 766 (Z): Greece, Karpathos, Between Ammopi and Akrotiri Volakas	MH561025	MH582767	MH582856	-
***L. carpetanicum* Erben**	M. Nydegger 37193 [P05091517] (P): Spain, Ciudad Real, Alcazar de San Juan	MH561049	MH582713	-	-
***L. carthaginense* (Rouy) C.E.Hubb. & Sandwith**	M.W. Chase 705 (K)	MW289862*	MW289876*	MH582912	MH582582
***L. cephalonicum* R.Artelari**	K. Kougioumoutzis 20 Oct. 2014 (UPA): Greece, Ionian islands, Ithaki, Agia Ierousalim	MH561059	MH582673	MH582913	MH582599

***L. chersonesum* Erben & Brullo**	K. Koutroumpa 44 (Z): Greece, Crete, Hersonissos	MH561015	MW289895*	MH582867	-
***L. circaei* Pignatti**	Jürg Röthlisberger 14/5/99 [Z-000102468] (Z): Italy, Monte circeo, Lazio	MH561071	MH582674	MH582914	MH582583
***L. compactum* Erben & Brullo**	K. Koutroumpa 926 (Z): Greece, Peloponnese, Viglafia, Pounta beach	MH560971	MH582732	MH582842	-
***L. confusum* (Godr. & Gren.) Fourr.**	K. Koutroumpa 1006 (Z): France, Leucate	MH561082	MH582675	MH582945	-
***L. connivens* Erben**	T. Borsch 5072 (B): Spain, Mallorca	MF083916	-	MF190442	MF041930
***L. contortirameum* (Mabille) Erben**	K. Koutroumpa 1007 (Z): Corse, Tallare	MH561098	MH582676	MH582938	MH582586
***L. cordatum* (L.) Mill.**	Jürg Röthlisberger 19/7/2002 (Z): France, Nice	MH561083	MH582693	MH582946	MH582597
***L. cornarianum* Kypr. & R.Artelari**	K. Koutroumpa 751 (Z): Greece, Crete, Moni Kaspa gorge	MH561069	MH582694	MH582900	MH582565
***L. coronense* R.Artelari**	Gioume Ioanna No. 1 (UPA): Greece, Peloponnese, Kastro Koronis	MH561088	MH582677	MH582915	MH582598
***L. corsicum* Erben**	K. Koutroumpa 1009 (Z): Corse, Galeria	MH561085	MH582678	MH582916	MH582581
***L. cossonianum* Kuntze**	Lledó, M.D. & M.B. Crespo 7-IX-94, No. 10729 (ABH), [K000696171] (K): Spain, New Town, Alicante; Miquel A. Conesa 2009-11: Spain: Formentera; Palacios et al. JAR-97005: Baleares, Formentera	MW289860*	KJ608017	MH582962	AJ132331
***L. costae* (Willk.) Pignatti**	A. Jiménez, S. Blasco AJ287-1 (Z): Spain, Cuenca, El Hito	MH561097	-	MW289851*	MW290504*
***L. cosyrense* (Guss.) Kuntze**	K. Koutroumpa 1010 (Z)	MH561060	MH582679	MH582917	-
***L. crateriforme* Erben & Brullo**	K. Koutroumpa 151 (Z): Greece, Crete, Akrotiri Mavromouri	MH561016	MH582768	MH582857	MH582560
***L. creticum* R. Artelari**	K.Koutroumpa, M.Megariti, S.Pirintsos 2013.09.11-01 (Hb.UoC): Greece, Crete, Matala caves	MH561008	MH582762	MH582875	MH582575
***L. cumanum* (Ten.) Kuntze**	H. Akhani 16815 (Hb. Akh.): Italy, Naples, Capri Islands	MF083856	-	MF190382	JX983717
***L. cylindrifolium* (Forssk.) Verdc. ex Cufod.**	P. Hein YP3421 [B 100642219] (B): Yemen, Hadhramaut; I.S. Collenette 9246 [E00121225] (E): Saudi Arabia, Farasan Island, Al Husain Bay	MF083909	-	MH582888	MF041923

***L. cymuliferum* (Boiss.) Sauvage & Vindt**	Davis, P.H. 53418 [E00770648] (E): Algeria, Djelfa	MH561047	MH582724	-	MH582612
***L. cythereum* R.Artelari & Georgiou**	K. Koutroumpa 902 (Z): Greece, Kythira, Chalkos bay	MH561055	MH582680	MH582904	MH582554
***L. daveaui* Erben**	M.M. Romeiras 410 (LISC): Portugal, Estuário do Tejo	MH561078	MH582700	MH582918	MH582609
***L. delicatulum* (Girard) Kuntze**	M.D. Lledó & M.B. Crespo 7-IX-94, No 10730 (ABH), [K000696170] (K): Spain, Alicante, Santa Pola; Palacios et al. JAR-96018: Spain, Alicante, Cala Blanca, Javea	MW289868*	MW289880*	MH582963	AJ222851
***L. dendroides* Svent.**	A. Jiménez AJden4 (Z): Spain, Canary islands, La Gomera, El Azadoe	MW289872*	MH582796	MH582883	MH582625
***L. densissimum* (Pignatti) Pignatti**	Curco et al. 15-X-94 (ABH): Spain	MW289864*	MW289882*	MH582919	MH582610
***L. dichotomum* (Cav.) Kuntze**	A. Jiménez, S. Blasco AJ285 (Z): Spain, Madrid, Rivas Vaciamadrid; Palacios et al. JAR-96501: Spain, Madrid, Aranjuez	MH561092	MH582704	MH582920	AJ222858
***L. dichroanthum* (Rupr.) Ikonn.-Gal.**	K.R. Robertson, J.B. Taft, G.A. Lazkov 6076 [E00711141] (E): Kyrgyzstan, Naryn Oblasty	MH561104	MH582800	MH582971	-
***L. dodartii* (Girard) Kuntze**	K. Koutroumpa 1011 (Z): France, Vendée, Noirmoutier en l ìle, Roche Biron	KP159568	MH582701	MH582921	MH582606
***L. dufourii* (Girard) Kuntze**	ABH 670 (ABH); M.D. Lledó & M.B. Crespo s.n. (ABH): Spain; Palacios et al. JAR-96051: Spain, Valencia, Cullera	AJ391326	MW289877*	MH582922	AJ222840
***L. ebusitanum* (Font Quer) Font Quer**	Miquel A. Conesa 2009-38; G.Sag 1987-6-2 [P05086682] (P): Spain, Mallorca, Cap Regana	-	KJ608043	-	MH582603
***L. echioides* (L.) Mill.**	K. Koutroumpa 579 (Z): Greece, Gavdos, Korfos; Miquel A. Conesa 2009-47: Spain, Mallorca	MH561038	KJ608051	MH582966	MH582618
***L. effusum* (Boiss.) Kuntze**	E. Cabi & F. Celep 3916 (GAZI): Turkey, Balıkesir, Ayvalık	MH560972	MH582733	MH582966	MH582545
***L. elaphonisicum* Alf.Mayer**	K. Koutroumpa 589 (Z): Greece, Gavdos, Sarakiniko	MH561012	MH582769	MH582868	-
***L. erectum* Erben**	K. Koutroumpa 1012 (Z): Spain, Madrid, Alcalá de Henares	MH561093	MH582705	MH582950	MH582594
***L. estevei* Fern.Casas**	No 9231 (ABH), [K000696167] (K): Spain, Mojacar, towards Carboneras	MW289861*	MW289881*	MH582968	MH582570

L. failachicum **Erben & Mucina**	A. Rawi 10838 [E00068909] (E): Kuwait, Failaka	MH560987	MW289885*	MH582853	MF041934
L. fallax **(Coss. ex Wangerin) Maire**	P. Davis & J.D. Davis 48593 [E00770646] (E): Morocco, Oued Dra between Goulimime and Tan-Tan	MH561004	MH582753	MH582895	MH582530
L. ferganense **Ikonn.-Gal.**	Zaborov s.n. (MHA): Kyrgystan, Alai Range	MF083872	-	MF190398	MF041890
L. flexuosum **(L.) Kuntze**	F. Karo 199 [E00770640] (E): Russian Federation, Nerczynsk	MH561105	MH582798	MH582972	MH582515
L. frederici **(Barbey) Rech.f.**	K. Koutroumpa 818 (Z): Greece, Karpathos, Sokastro (Esokastro) islet	MH561028	MH582785	MH582878	MH582572
L. frutescens **(Lem.) Erben, A.Santos & Reyes-Bet.**	A. Jiménez AJ276-1 (Z): Spain, Canary Islands, Tenerife, Buenavista, Teno	MH561123	MW289901*	MH583007	MH582642
L. furfuraceum **(Lag.) Kuntze**	K. Koutroumpa 1013 (Z): Spain, Alicante, Valencia, Playa de San Juan; Palacios et al. JAR-96219: Spain, Alicante, Cabo Huertas	MW289865*	MW289883*	MH582947	AJ222856
L. gibertii **(Sennen) Sennen**	Royl 944 [B 10 0663742] (B): Spain, Mallorca, Cabo Blanco	MF083897	-	MF190423	-
L. girardianum **(Guss.) Fourr.**	M.D. Lledó & M.B. Crespo 11-XI-94, No 10732 (ABH), [K000696172] (K): Spain, Valencia, Dehesa Saler, Mallada del Garrofer; Palacios et al. JAR-96027: Spain, Valencia, El Saler	MW289869*	MH582720	MH582964	AJ222845
L. globuliferum **(Boiss. & Heldr.) Kuntze**	K. İldeniz & F. Celep 3936 (GAZI): Turkey, Aksaray, between Eskil and Gölyazı	MW289855*	MW289888*	MH582850	MH582549
L. gmelini **(Willd.) Kuntze**	K. İldeniz & F. Celep 3943 (GAZI): Turkey, Aksaray, from Aksaray to Konya; Joharchi & Zangui 10637: Iran, north-west of Bojnurd, Shahrabad	MH560975	MH582740	MH582844	JX983716
L. gougetianum **(Girard) Kuntze**	K. Koutroumpa 1014 (Z)	MH561061	MH582681	MH582923	-
L. grabusae **Erben & Brullo**	K. Koutroumpa 464 (Z): Greece, Crete, Imeri Gramvousa	MH561009	MH582779	MH582869	MH582555
L. graecum **(Poir.) Rech.f.**	E. Cabi & F. Celep 3906 (GAZI): Turkey, İzmir, Çeşme, Altınkum	MH561023	-	MH582870	MH582557
L. greuteri **Erben**	K. Koutroumpa 1015 (Z): Corse, La Revellata	MH561081	MH582682	MH582948	MH582585

	M.F. Gardner & C.N. Page 5110 [E00215437]				
L. guaicuru **(Molina) Kuntze**	(E): Chile, Región Valparaíso, Zapallar	MH560990	MH582737	MH582845	MH582536
L. gueneri **Doğan & H.Duman & Akaydın**	Duran 9408 (Hb. A. Duran): Turkey	MF083853	-	MF190379	MF041873
L. gymnesicum **Erben**	K. Koutroumpa 1016 (Z); Miquel A. Conesa 2009-41:Spain, Mallorca; Palacios et al. JAR-94328: Spain, Mallorca, Sant Pere	MH561062	KJ608045	MH582939	AJ222842
L. haitense **Blake**	T. Zanoni & M. Mejía P.18010 [MEXU 592920] (MEXU): Dominican Republic, Monte Cristi	MF083914	-	MF190440	MF041928
L. hibericum **Erben**	A. Jiménez, S. Blasco AJ297 (Z): Spain, Zaragoza, Bujaraloz, La Playa lagoon	MH561100	MH582708	MH582924	MH582589
L. hierapetrae **Rech.f.**	K.Koutroumpa, M.Megariti, S.Pirintsos 2013.09.08-11 (Hb.UoC): Greece, Crete, Ferma	MH561050	MH582668	MH582937	-
L. hoeltzeri **(Regel) Ikonn.-Gal.**	Dürbye 1688 [B 100099104] (B):Kyrgystan, Tian-Shan, Issyk-Kul area; Phillippe Loy R., Taft John B., Dietrich Christopher H., Warren Emily, Lazkov Georgy A. 30924 [E00714953] (E): Kyrgyzstan, Issyk-kul. Taragay River	MF083886	MH582794	MH582976	MH582513
L. humile **Mill.**	Kristina Bjureke, Tor S. Mjaaland 04.10.2015 (Z): Norway, Oslo, Gressholmen	MH560974	JN893200	JN894792	-
L. hungaricum **Klokov**	HU-0-MSTR-5727/20140257 (Botanischer Garten der WWU-MS-Münster); NMW2915 (NMW) [POWNA2741-12]	MH560973	MH582734	MH582849	MH582541
L. hyblaeum **Brullo**	K. Koutroumpa 1017 (Z)	MH561072	MH582683	MH582925	MH582608
L. iconicum **(Boiss. & Heldr.) Kuntze**	F. Celep 3919 (GAZI): Turkey, Ankara, Şereflikoçhisar	MH561110	MH582791	MH582980	MH582520
L. imbricatum **(Webb ex Girard) Hubbard ex L.H.Bailey**	A. Jiménez AJ283-1 (Z): Spain, Canary Islands, Tenerife, Punta del Hidalgo	MH561132	MH582810	-	MH582639
L. insigne **(Coss.) Kuntze**	K. Lewejohann & J. Müller 97-473 (GOET):Spain, Almeria; A. Jiménez, S. Blasco AJ213-1 (Z): Spain, Murcia, Calabardina, road to Aguilas	MF083880	MH582781	MH582880	MH582622
L. iranicum **(Bornm.) Lincz.**	I.C. Hedge, P. Wendelbo & H. Foroughi 14677 [E00453664] (E): Iran, Tehran, Near Mardabad (South of Karaj); Akhani & Pahlevani 20820	MH560986	MH582730	MH582854	JX983712

	(Hb. Akh): Iran, north-west of Persian Gulf, Dayreh Island				
***L. jovi-barba* (Webb) Kuntze**	C. Fernandes & M.M. Romeiras s.n. (LISC): Cape Verde. S. Vicente, Monte Verde; Duarte et al. 4142 (LISC): Cape Verde, São Nicolau, Ribeira Funda, Fundo Cruz	MH561007	MW289890*	MW289849*	MH582534
***L. kardamylii* R.Artelari & Kamari**	K. Koutroumpa Pel.01 (Z): Greece, Peloponnese, Kardamyli	MH561089	MH582684	MH582926	MH582587
***L. kaschgaricum* (Rupr.) Ikonn.-Gal.**	L.R. Phillippe, J.B. Taft, C.H. Dietrich, E. Warren, G.A Lazkov 30890 [E00714808] (E): Kyrgyzstan, Issyk-Kul, Kadzhi-Saj	MH561107	MH582795	MH582977	MH582514
***L. kraussianum* (Buchinger ex Boiss.) Kuntze**	G. Geraishuizen 4146 [WAG.1169568] (WAG): South Africa, Ratelrivier vlei	MH561070	-	-	MH582591
***L. lanceolatum* (Hoffmanns. & Link) Franco**	M.M. Romeiras 439 (LISC): Portugal, SW Alentejo, Vila Nova Mil Fontes	MH561041	MH582709	MH582957	MH582567
***L. latebracteatum* Erben**	M. Nydegger 37239 [P05090887] (P): Spain, Lleida, Ivars d'Urgell, Castellsera	MH561101	MH582710	MH582927	-
***L. latifolium* (Sm.) Kuntze**	Jürg Röthlisberger 10/8/1999 [Z-000102467] (Z): Romania, Ruinengelände, Histria, Judetul, Constanta; N.M. Reshetnikov, A.K. Mamontov & M.I. Popchenko s.n. (MHA): Russia, Belgorod Oblast, Rovensky area, Aydar	MH560977	MH582741	MF190393	MH582546
***L. laxiusculum* Franco**	M. Nydegger 36898 [P05093581] (P): Portugal, Estremadura, Praia de Magoito sur Ericeira	MH560991	-	-	MH582602
***L. lilacinum* (Boiss. & Bal.) Wagenitz**	F. Celep 3922 (GAZI): Turkey, Ankara, Şereflikoçhisar; H. Akhani & al. 17930 (Hb. Akh.): Turkey, Ankara, around Mogan Gol Lake	MH560968	MH582746	MH582851	JX983693
***L. limbatum* Small**	R. D. Worthington 18488 [L.2643555] (L): America, United States of America, Texas. Reevs Co.: Pecos	MH560980	MH582738	-	MH582537
***L. lobatum* (L.f.) Chaz.**	A. Jiménez AJ272-3 (Z): Spain, Canary Islands, Tenerife, Arico, El Viso; Palacios et al. VAB-96/4628: Spain, Almeria, Terreros	MH561137	MW289903*	MH582998	AJ132333
***L. lobinii* N.Kilian & Leyens**	C. Fernandes & M.M. Romeiras s.n. (LISC): Cape Verde, Santiago Island, Serra Malagueta;	MH561006	MW289892*	MW289850*	MW290503*

	M. Romeiras et al. 947 (LISC): Cape Verde, Santiago, Serra da Malagueta				
***L. longebracteatum* Erben**	A. Jiménez, S. Blasco AJ286-1 (Z): Spain, Cuenca, El Hito, El Hito lagoon	MH561039	MH582721	MH582965	MH582617
***L. lowei* R.Jardim, M.Seq., Capelo, J.C.Costa & Rivas Mart.**	A. Santos-Guerra & F. Fernández AS 3565-11, ORT 42924 (ORT): Madeira, Porto Santo Island, capital	MH561037	MH582717	MH582958	MH582568
***L. macrophyllum* Kuntze**	A. Jiménez AJ274-1 (Z): Spain, Canary Islands, Tenerife, Chamorga, Montaña Tafada	MH561125	MW289897*	MH583003	MH582644
***L. macropterum* (Webb & Berthel.) Kuntze**	A. Santos-Guerra ORT s.n. (ORT): Spain, Canary islands, Riscos Bascos, El Hierro island	MH561127	MH582803	MH583002	MH582634
***L. majus* (Boiss.) Erben**	M. Nydegger 37115 [P05090885] (P): Spain, Granada, Baza, Cullar-Baza ne Venta del Peral	MH561042	-	-	-
***L. meandrinum* Erben & Brullo**	K. Koutroumpa 834 (Z): Greece, Karpathos, Apella	MH561091	MH582685	MH582906	-
***L. meyeri* (Boiss.) Kuntze**	Edinburgh Tbilisi Expedition to Georgia (2009) 18 [E00375757] (E): Georgia, Davit Gareji; Kazempour Osaloo Tarbiat Modares University Herbarium 89213: Iran	MH560976	MH582742	MH582846	AB979593
***L. milleri* Ghaz. & J.R.Edm.**	I.S. Collenette 8418 [E00046382] (E): Oman, Thamrait	MH560996	MH582759	MH582889	MH582521
***L. minoicum* Erben & Brullo**	K. Koutroumpa 313 (Z): Greece, Crete, Tertsa	MH561051	MH582686	MH582928	-
***L. minutiflorum* (Guss.) Kuntze**	K. P. Buttler Nr.18747 (UPA): Italy, Sizilien, Capo di Milazzo	MH561063	MH582696	-	MH582577
***L. minutum* (L.) Chaz.**	K. Koutroumpa 1018 (Z); Miquel A. Conesa 2009-21:Spain, Menorca; Palacios et al. JAR-97006: Spain, Baleares, Formentera	MH561073	KJ608027	MH582929	AJ132332
***L. mouretii* (Pitard) Maire**	P. Brownless 566 [E00706083] (E) Morocco, Meknès, Bekrit; cult. M. Erben, Munich	MH561138	MH582814	AF204854	MH582629
***L. mucronatum* (L.f.) Chaz.**	Salmon, M. & Fillan, M. 22/3 [E00770647] (E): Morocco, Between Tamri and Agadie	MH561005	MH582754	MH582896	MH582531
***L. mucronulatum* (H.Lindb.) Greuter & Burdet**	Christodoulou s.n. (B): Cyprus, Larnaka, Larnaka Salt Lake	MF083893	-	MF190419	MF041909
***L. multiflorum* Erben**	M. M. Romeiras 422 (LISC): Portugal, Cascais, Guincho, Cabo Raso	MH561079	MH582707	MH582936	MH582613

***L. multiforme* (Martelli) Pignatti**	E. Georgiadou & A. Strid 364 (ATH): Italy, Prov. of Toscana, Parco della Maremma	MH561064	MH582722	MH582930	MH582584
***L. narbonense* Mill.**	H. Akhani 20504 (Hb. Akh.): France, Alpes-Cote d'Azur; K. Koutroumpa 941: Greece, Patras, Rio	MF083875	MW289886*	MF190401	MW290501*
***L. nudum* (Boiss. & Buhse) Kuntze**	H. Akhani & al. 18804 (Hb. Akh.): Iran, Semnan	MF083929	-	MF190458	JX983672
***L. nydeggeri* Erben**	K. Koutroumpa 1019 (Z): Portuga, Lisboa, Parque Botânico da Tapada Da Ajuda	MH561043	MH582718	MH582959	-
***L. obtusifolium* (Rouy) Erben**	K. Koutroumpa 1020 (Z): Corse, Bonifacio, ilot du Fazzio	MH561075	MH582688	MH582931	MH582593
***L. ocymifolium* (Poir.) Kuntze**	O. Georgiou 11 (UPA): Greece, Milos, Fyropotamos	MH561102	MH582780	MH582932	-
***L. oligotrichum* Erben & Brullo**	K. Koutroumpa 212 (Z): Greece, Crete, Xerokampos	MH561031	MH582770	MH582858	MH582558
***L. otolepis* (Schrenk) Kuntze**	E. McClintock [E00770643] (E); Malekmohammadi 3897 (Hb. Akh.): Iran	MH561111	MH582792	MH582982	JX983682
***L. ovalifolium* (Poir.) Kuntze**	K. Koutroumpa 1021 (Z): France, Loire-Atlantique, Le Croisic, Rocher de L`Ours	MH561044	MH582719	MH582960	MH582569
***L. palmyrense* (Post) Dinsm.**	Hunting Aero Survey 127D [E00453655] (E): Jordan, El Umari	MH560988	MH582729	-	MH582535
***L. papillatum* (Webb & Berthel.) Kuntze**	A. Jiménez, A. Santos-Guerra AJ262 (Z): Spain, Canary Islands, Fuerteventura, Jandía	MH561000	MH582750	MH582897	MH582528
***L. paulayanum* (Vierh.) Ghaz. & J.R.Edm.**	A.G. Miller, L. Guarino, N. Obadi, M. Hassan & N. Mohammed 8526 [E00453714] (E): Yemen, Socotra, Wadi Irih	MH560992	MH582757	-	MH582522
***L. pectinatum* var. *corculum* (Webb & Berthel.) G.Kunkel & Sunding**	A. Jiménez AJ284-1 (Z): Spain, Canary Islands, Tenerife, Puerto de la Cruz	MH561001	MW289889*	MH582892	MH582532
***L. pectinatum* var. *divaricatum* (Pit.) G.Kunkel & Sunding**	A. Santos-Guerra AS 3342-11 (ORT): Spain, Canary Islands, Tenerife, Las Galletas	MH561002	MH582749	MH582893	MH582533
***L. pectinatum* var. *solandri* (Webb. & Berthel.) Kuntze**	A. Santos-Guerra AS 3315-11 (ORT): Spain, Canary Islands, El Hiero, Arenas Blancas	MH561003	MH582751	MH582894	MH582529
***L. peregrinum* (P.J.Bergius) R.A.Dyer**	M. Weigend 2703 (M): South Africa, Dwarskersbos; Stephen Boatwright, Olivier Maurin, Simeon Bezeng, Kowiyou Yessoufou JWB514: South Africa, Western Cape	MF083838	JQ412383	MF190444	MF041932

L. perezii **(Stapf) Hubbard ex L.H.Bailey**	A. Jiménez AJ303-45 (Z): Spain, Canary islands, Tenerife, Masca, Roque Tarucho	MH561135	MH582804	MH583005	MH582640
L. perfoliatum **(Kar. ex Boiss.) Kuntze**	T.F. Hewer 4044 [E00453647] (E): Iran, Golestan, Gorgan; Akhani & Zangui 10130 (Hb. Akh.): Iran, Chahchaheh towards Kalat-e Naderi	MH561112	MH582793	MH582981	JX983681
L. pigadiense **(Rech.f.) Rech.f.**	K. Koutroumpa 777 (Z) Greece, Karpathos, Damatria	MH561053	MH582689	MH582905	-
L. piptopodum **Nevski**	G.M. Proskoorykova s.n. (MHA): Turkmenistan, Mts. Kugitang, Charshangu	MF083866	-	MF190392	MF041884
L. platyphyllum **Lincz.**	K. Koutroumpa 1022 (Z)	MH560978	MH582743	MH582847	MH582542
L. plurisquamatum **Erben**	M.M. Romeiras 445 (LISC): Portugal, S. Martinho do Porto	MH561087	MH582702	MH582940	-
L. preauxii **(Webb & Berthel.) Kuntze**	A. Jiménez AJ316 (Z): Spain, Canary Islands, Gran Canaria, Fataga ravine	MH561124	MH582805	MW289852*	MH582636
L. proliferum **(d'Urv.) Erben & Brullo**	K. Koutroumpa 118 (Z): Greece, Crete, Moni Faneromenis	MH561017	MH582771	MH582863	MH582562
L. pruinosum **(L.) Chaz.**	P. Davis & J.D. Davis 49143 [E00770644] (E): Morocco, N. of Erfoud	MH561032	MH582772	MH582881	MH582621
L. pseudebusitanum **Erben**	D. Bramwell & Z.I. Bramwell 38 [E00240957] (E): Spain, Balearic Islands, Majorca, Cabo Blanco; Miquel A. Conesa 2009-27: Spain, Menorca	MH561065	KJ608033	MH582902	MH582578
L. puberulum **(Webb) Kuntze**	A. Jiménez AJ222-2 (Z): Spain, Canary Islands, Lanzarote, Haría	MH561134	MH582811	MH582996	MH582631
L. purpuratum **Hubbard ex L.H.Bailey**	Kew DNA bank 2355, Fred Mayer s.n.: South Africa	MW289854*	MW289884*	AY042537	MW290500*
**L. cf.** ***pycnanthum*** **(K. Koch) Kuntze**	K. İldeniz & F. Celep 3931 (GAZI): Turkey, Aksaray, Hamidiye village	MH560969	MH582747	MH582852	MH582548
L. pylium **R.Artelari**	Gioume Ioanna No. 4 (UPA): Greece, Peloponnese, Matzakoura beach	MH561066	MH582690	MH582954	MH582588
L. recticaule **Erben & Brullo**	K. Koutroumpa 26 (Z): Greece, Crete, Malia	MH561018	MH582773	MH582859	MH582561
L. recurvum **C.E.Salmon subsp.** ***humile*** **(Girard) Ingr.**	McHaffie, Heather & Frachon, Natacha, Garden collection number: 3451, RBGE Accession number: 20100965 B, [E00668930] (E): United	MH561080	MH582703	MH582933	-

	Kingdom, Scotland, (VC 74) Wigtownshire, Mull of Galloway				
L. redivivum **(Svent.) G.Kunkel & Sunding**	A. Jiménez AJ268 (Z): Spain, Canary Islands, La Gomera, Benchijigua	MH561129	MH582807	MH583001	MH582635
L. relicticum **R.Mesa & A.Santos**	A. Santos-Guerra AS 2790-09 (ORT): Spain, Canary islands, La Gomera, Teguerguenche	MH561131	MH582806	MH583000	MH582633
L. remotispiculum **(Lacaita) Pignatti**	Mario Coiro s.n. (Z): Italy, Lido Macarro, Marina di Maratea	MH561074	MH582691	MH582953	MH582601
L. reniforme **(Girard) Lincz.**	Malekmohammadi & al. 3970 (Hb. Akh.):Iran, Fars; Khosravi & Tahari s.n. (SHIRAZ): Iran, Fars Province, Darab	MF083931	-	MF190460	JX983675
L. rigualii **M.B.Crespo & Erben**	Lledo & Crespo LL10 (ABH); Palacios et al. JAR-96126: Spain, Alicante, Cala Blanca, Javea	AJ391328	GQ248628	AM889717	AJ222854
L. roridum **(Sibth. & Sm.) Brullo & Guarino**	K. Koutroumpa 582 (Z): Greece, Gavdos island, Sarakiniko	MH561013	MH582774	MH582871	MH582556
L. santapolense **Erben**	M.D. Lledó, M.D. & M.B. Crespo 3-VI-94, No 9224 (ABH), [K000696175] (K): Spain, Dehesa Saler, Mallada del Garrofer	MW289866*	MW289878*	MH582934	-
L. saracinatum **R.Artelari**	K. Kougioumoutzis 18 Oct. 2014 (UPA): Greece, Ithaki island, South Kaminia	MH561067	MH582692	MH582949	MH582600
L. sarcophyllum **Ghaz. & J.R.Edm.**	I.M. McLeish 3392 [E00132418] (E): Oman, Bimma, Sharquyah	MH560997	MH582760	MH582884	MH582523
L. scabrum **(Thunb.) Kuntze**	M.W. Chase 5889 (K): South Africa	MH561068	AM235050	MH582942	MH582590
L. scopulorum **M.B.Crespo & Lledó**	E. Camuñas, L. Serra & M.B. Crespo 3-VIII-94 (K), No 9723 (ABH): Spain, Alicante, Denia, Cova Tallada	MW289863*	MH582695	MH582969	-
L. sieberi **(Boiss.) Kuntze**	K. Koutroumpa 502 (Z): Greece, Crete, Almyrida	MH561099	MH582723	MH582935	MH582611
L. sinense **(Girard) Kuntze**	20141577 (HSNU): China	JQ946308	JQ946306	JQ946307	MF063810
L. sinuatum **(L.) Mill.**	Podlech s.n. [B 10 0546980] (B): Spain, Marroco; K. Koutroumpa 53 (Z): Greece, Crete, Kokkini Chani; Palacios et al. JAR-96850: Spain, Almeria, C. Gata	MF083889	MW289902*	MH582992	AJ222860
L. sitiacum **Rech.f.**	K. Koutroumpa 90 (Z): Greece, Crete, Pacheia Ammos	MH561030	MH582783	MH582877	-

L. sogdianum **Ikonn.-Gal.**	H. Akhani & F. Memariani 19055 (Hb. Akh.): Iran, Khorassan, Between Neyshabur and Kashmar	MF083933	-	MF190462	JX983723
L. sokotranum **(Vierh.) Radcl.-Sm.**	A.G. Miller, L. Guarino, N. Obadi, M. Hassan & N. Mohammed M.8503 [E00676679] (E): Yemen, Socotra, near Ras Qatanhin	MH560993	MH582758	MH582885	MH582524
L. somalorum **(Vierh.) Hutch. & E.A.Bruce**	Kew DNA bank 1921	MH560994	MH582761	MH582886	MH582525
L. sougiae **Erben & Brullo**	K. Koutroumpa 342 (Z): Greece, Crete, Sougia	MH561014	MH582782	-	MH582553
**L. sp.1**	K. İldeniz & F. Celep 3925 (GAZI): Turkey, Seyfe Lake	MH561109	MH582790	MH582979	MH582519
**L. sp.2**	K. Koutroumpa 300 (Z): Greece, Crete	MH561052	MH582687	MH582901	-
L. spectabile **(Svent.) G.Kunkel & Sunding**	A. Santos-Guerra AS 2634-09 (ORT): Spain, Canary islands, Tenerife, Barranco (Ravine) de Natero	MW289875*	MW289899*	-	MH582641
L. spreitzenhoferi **Erben & Brullo**	K. Koutroumpa 862 (Z): Greece, Kythira, Fournoi beach	MH561022	MH582775	MH582873	MH582563
L. stenotatum **(Rech.f.) Erben & Brullo**	K. Koutroumpa 143 (Z): Greece, Crete, Agia Fotia	MH561019	MH582786	MH582860	MH582559
L. stocksii **(Boiss.) Kuntze**	H. Akhani & al. 23616 (Hb. Akh.): Iran, Sistan-o Baluchestan	MF083852	-	MF190378	MF041872
L. subglabrum **Erben**	M. Nydegger 37001 [P05090886] (P): Spain, Granada	MH561045	MH582711	MH582967	MH582596
L. suffruticosum **(L.) Kuntze**	G.M Proskuriakova 94 [E00453658] (E): Azerbaijan, Caucasus, Districtus Schemakha, montes Malyi Harami; H. Akhani 20364 (Hb. Akh.): Uzbekistan, Kyzylkum	MH560995	MH582728	MF190464	JX983671
L. sundigii **Leyens, Lobin, N.Kilian & Erben**	Kilian & Leyen 3184 (B): Cape Verde Islands, S. Nikolau, Alto das Cabacas; Duarte n.s. (LISC): Cape Verde, São Nicolau, Alto das Cabaças	MF083887	MW289891*	MF190413	MF041903
L. supinum **(Girard) Pignatti**	A. Jiménez, S. Blasco AJ300 (Z): Spain, Alicante, Alcoy, above El Molinar	MH561096	MH582697	MH582943	-
L. sventenii **A. Santos & M.L.Fernández**	A. Jiménez, E. Conti, H. Schäfer AJ197-1 (Z): Spain, Canary Islands, Gran Canaria, San Isidro, Montaña Amagro	MW289874*	MW289900*	-	MH582637

L. tabernense **Erben**	A. Jiménez, S. Blasco AJ295-1 (Z): Spain, Almería, Tabernas	MH561046	MH582714	-	MH582566
L. tamaricoides **Bokhari**	Akaydın 9232 [B 100417679] (B): Turkey, Kirshehir, Badili village	MF083862	-	MF190388	MF041880
L. tenellum **(Turcz.) Kuntze**	Kew DNA bank 2360, Fred Mayer s.n.	MW289871*	MW289904*	MH582974	MH582516
L. tetragonum **(Thunb.) Bullock**	Y. Pillon et al. 409 (NOU): New Caledonia, Prov. Du Noumea, Pointe Maa	MW289870*	MH582797	MH582975	MW290498*
L. thiniense **Erben**	Chase 1483 (K): Spain	-	GQ248629	AM889718	-
L. toletanum **Erben**	K. Koutroumpa 1023 (Z)	MH561094	MH582726	MH582952	MH582595
L. tomentellum **(Boiss.) Kuntze**	RBGE Accession number: 19695219/ 19695219A, R31 Rock Garden	MH560979	MH582744	MH582848	MH582543
L. tournefortii **(Boiss.) Erben**	M. Nydegger 36858 [P05090889] (P): Spain, Toledo, La Guardia	MH561095	MH582706	MH582951	-
L. tuberculatum **(Boiss.) Kuntze**	A. Jiménez, A. Santos-Guerra AJ257 (Z): Spain, Canary Islands, Lobos islet, Las Lagunillas	MW289859*	MW289896*	MH582882	MH582619
L. tubiflorum **(Del.) Kuntze**	Boetje-van Ruyven, MRS 91 [L.2644102] (L): Egypt, Abusir (Maryut)	MH561033	-	-	MH582624
L. tunetanum **(Barratte) Maire**	P.H. Davis 48092 [E00770645] (E): Tunisia, El Djerid	MH561048	MH582712	-	MH582573
L. vanandense **Erben & Brullo**	I. Bazos 4582 (UPA): Greece, Karpathos, Tristomo	MH561024	MH582776	MH582874	-
L. vigaroense **Marrero Rodr. & R.S.Almeida**	Oscar Saturno Hernández Banco_ADN_2723, DNA bank of the Canarian Flora, Jardín Botánico Canario “Viera y Clavijo” – Unidad Asociada CSIS: Canary islands, Gran Canaria, Barranquillo de Las Magarzas	MH561126	MH582813	MH582997	MH582638
L. virgatum **(Willd.) Fourr.**	K.Koutroumpa, M.Megariti, S.Pirintsos 2013.09.11-16 (Hb.UoC): Greece, Crete, Kalamaki; Miquel A. Conesa 2009-52: Spain Mallorca	MH561054	KJ608053	MH582941	MH582614
L. vulgare **Mill.**	Westendorp R. 12.09.2013, NL-0-AMD-20130258 (Botanical Garden UZH): Netherlands, Ameland, nabij Veerdam	MW289856*	MW289887*	MW289847*	MW290502*

***L. vulgare* (*eduardi-diasii* Fdez. Prieto & C. Aguiar sp. nova. ined.)**	Mónica Moura LI-MAMA-001, AZB 1570 (AZB): Azores, Santa Maria, Maia	MH560982	MH582735	MW289846*	MH582538
***L. wrightii* (Hance) Kuntze**	H. Izumi & M. Fujimoto 78 [E00770639] (E): Japan, Kyushu, Kagoshima, Kurio, Shimoyaku-mura, Yakushima Island	MH561106	MH582799	MH582973	MH582517
***L. xerocamposicum* Erben & Brullo**	K. Koutroumpa 215 (Z): Greece, Crete, Xerokampos	MH561027	MH582777	MH582861	-
***L. xiliense* Erben & Brullo**	K. Koutroumpa 930 (Z): Greece, Peloponnese, Archangellos	MH561026	MH582778	MH582862	MH582551
**Species of other Plumbaginaceae genera**					
***Acantholimon acerosum* (Willd.) Boiss.**	Chase 709 (K); Ern & Krone 6896 (B)	AJ391314	-	-	LT714475
***Acantholimon bracteatum* (Girard) Boiss.**	ERE 175833	LT714372	-	-	LT714572
***Acantholimon chitralicum* Rech.f. & Schiman-Czeika**	B. Dickore 13496 (B)	LT714384	-	-	LT714584
***Acantholimon cymosum* Bunge**	Isolate PLU384 BGBM	LT714410	-	-	LT714600
***Acantholimon demavendicum* Bornm.**	Rechinger 6068 (B)	LT714295	-	-	LT714500
***Acantholimon diapensioides* Boiss.**	U. Wuendisch 1436 (GOET)	LT714386	-	-	LT714585
***Acantholimon echinus* (L.) Bunge**	A. Fokkinga 1969-06-14 [L.2647181] (L)	MH561146	MH582819	-	MH582646
***Acantholimon glutinosum* Rech.f. & Köie**	Rechinger 35069 (B)	LT714304	-	-	LT714509
***Acantholimon gorganense* Mobayen**	Rechinger 4809 (B)	LT714305	-	-	LT714510
***Acantholimon hohenackeri* (Jaub. & Spach) Boiss.**	Jennifer Lamond 3744 [Z-000102460] (Z); Assadi & Mozaffarian 29835 (TARI)	MH561148	MH582816	-	AB979563
***Acantholimon leucochlorum* Rech.f. & Schiman-Czeika**	Jennifer Lamond 2507 [Z-000102459] (Z); Rechinger 37287 (B)	MH561145	MH582823	-	LT714527
***Acantholimon lycopodioides* (Girard) Boiss.**	Ern & Prelaz 7616 (B)	LT714275	-	FN597642	LT714480
***Acantholimon pterostegium* Bunge**	Rechinger 56276 (B)	LT714337	-	-	LT714541
***Acantholimon restiaceum* Bunge**	Rechinger 55669 (B)	LT714343	-	-	LT714547
***Acantholimon revolutum* Rech.f. & Köie**	Rechinger 37474 (B)	LT714344	-	-	LT714548
***Acantholimon senganense* Bunge**	Jennifer Lamond & F. Terme 4345 [Z-000102457] (Z); Assadi 86957 (TARI)	MH561141	MH582817	-	AB979580
***Acantholimon solidum* Rech.f. & Köie**	Rechinger 36539 (B)	LT714350	-	-	LT714553
***Acantholimon subulatum* Boiss.**	Rechinger 37204 (B)	LT714355	-	-	LT714557

***Acantholimon tragacanthinum* (Jaub. & Spach) Boiss.**	Jennifer Lamond 3792 [Z-000102456] (Z); Assadi 86918 (TARI)	MH561147	MH582821	-	AB979585
***Acantholimon tricolor* Rech.f. & Köie**	Rechinger 32329 (B)	LT714358	-	-	LT714559
***Acantholimon ulicinum* (Schult.) Boiss.**	Akaydin 6699 (B)	LT714224	-	-	LT714429
***Acantholimon venustum* Boiss.**	J. Archibald 6700 [ZT-00077026] (ZT); DT 2143 (B)	MH561142	MH582822	-	LT714470
***Aegialitis annulata* R.Br.**	Chase 1629 (K); Christopher T. Martine 4043 (OC)	AJ312245	AJ312252	KY952312	-
***Armeria alliacea* (Cav.) Hoffmanns. & Link**	Hanspeter Schumacher s.n. (Z); Vogt s/n (MA)	MH561113	MH582825	MH582985	AJ225578
***Armeria arenaria* (Pers.) Schult.**	K. Koutroumpa 1029 (Z); isolate BF323	MH561118	KF997272	MH582987	MH582653
***Armeria canescens* (Host) Boiss.**	L. Giannakos 593 (UPA); Jury 17379 (IT)	MH561115	MH582826	MH582991	AY179770
***Armeria castellana* Boiss. & Reut. ex Leresche**	J.M. Gardiner & R.J.D McBeath R1035 [E00198386] (E)	MH561116	MH582830	MH582989	MH582655
***Armeria maritima* (Mill.) Willd.**	K. Koutroumpa 1028 (Z); GN 2866 (MA)	MH561119	MH582827	HM851064	AJ225574
***Armeria morisii* Boiss.**	K. Koutroumpa 1030 (Z)	MH561117	MH582828	MH582990	MH582654
***Armeria pseudarmeria* (Murray) Mansf.**	K. Koutroumpa 1031 (Z); Jury s/n (MA)	MH561120	MH582829	MH582988	AJ225596
***Armeria pungens* (Link) Hoffmanns. & Link**	M.J.Y. Foley 2206 [E00246339] (E); GN4587 (MA)	MH561114	MH582831	GQ901553	MH582656
***Armeria splendens* (Lag. & Rodr.) Webb**	Chase 1895 (K); Alvarez 1388 (MA)	AJ391316	Y16908	MH582986	AJ225591
***Bakerolimon plumosum* (F.Phil.) Lincz.**	Ehrhart 2002/238 (M); R. Baines, M. Gardner, P. Hechenleitner, C. Morter, & D. Rae 120 [E00230461] (E)	MF083840	MH582824	MH582983	MH582657
***Bukiniczia cabulica* (Boiss.) Lincz.**	K. Koutroumpa 1024 (Z)	MH561144	MH582818	MH583013	MH582645
***Cephalorhizum coelicolor* (Rech.f.) Rech.f.**	Volk 532 (B)	LT714368	-	-	LT714568
***Ceratolimon feei* (Girard) M.B.Crespo & M.D.Lledó**	Chase 1630 (K); S.L. Jury 19168 (RNG)	AJ391318	AJ286357	EU531681	HE602420
***Ceratolimon migiurtinum* (Chiov.) M.B.Crespo & M.D.Lledó**	Chase 1632 (K)	AJ391322	AJ286360	-	-
***Ceratolimon weygandiorum* (Maire & Wilczek) M.B.Crespo & M.D.Lledó**	Schouten 192 (LIV)	AJ391325	AJ286361	-	-
***Ceratostigma minus* Stapf ex Prain**	Chase 707 (K)	AJ391333	-	AY042566	-
***Ceratostigma plumbaginoides* Bunge**	K. Koutroumpa 1032 (Z)	MH561150	MH582838	MH583014	MH582659
***Dictyolimon macrorrhabdos* (Boiss.) Rech.f.**	Chase 710 (K); Nuesser 1050 (B)	AJ391317	Y16909	-	LT714570

***Dyerophytum africanum* (Lam.) Kuntze**	Edmondson (LIV); CA Mannheimer CM 996 [WAG.1169473] (WAG); Herman 27-II-1983 (K)	AJ312246	MW289907*	AY042581	MH582661
***Dyerophytum indicum* (Gibs. ex Wight) Kuntze**	Knees, MacKinnon, MacLaren & Page 215 [E00695995] (E)	MH561151	MH582832	MH583015	MH582662
***Goniolimon besserianum* (Schult.) Kusn.**	K. Koutroumpa 1025 (Z)	MH561140	-	MH583009	MH582650
***Goniolimon incanum* (L.) Hepper**	K. Koutroumpa 1026 (Z)	-	MH582815	MH583010	MH582651
***Goniolimon italicum* Tammaro, Pignatti & G.Frizzi**	K. Koutroumpa 1027 (Z)	-	-	MH583011	MH582648
***Goniolimon kaufmannianus* (Regel) Voss.**	L. Ivanina & L. Sergienko (B); [PE01589044] (PE)	LT714406	KX527533	KX526745	LT714605
***Goniolimon speciosum* (L.) Boiss.**	Karis 501 (S); Barnaul South Siberian Botanic Garden & Edinburgh Expedition to Russian Altai 50 [E00656075] (E)	AJ312247	AJ312254	MH583012	MH582652
***Goniolimon tataricum* (L.) Boiss.**	Gowe 41345 [B100112853] (B); Hanspeter Schumacher s.n. (Z)	LT714399	-	MW289853*	MH582649
***Limoniastrum guyonianum* Durieu ex Boiss.**	ABH 14933 (ABH); P.H. Davis 52372 [E00770652] (E); J. Lambinon 99/Tu/114 (RNG)	AJ391319	AJ286358	MH583008	HE602418
***Limoniastrum monopetalum* (L.) Boiss.**	ABH 10964 (ABH); Curco et al. 15-X-94 (K); Mateos, MA. 4825/95 (RNG)	AJ391321	Z97642	AY042609	HE602419
***Muellerolimon salicorniaceum* (F.Muell.) Lincz**	Craven L.A. 7128 (K), [CANB 379595.1] (CANB); P. Foreman SC560 [PERTH 08057176] (PERTH)	MF083839	-	MH582984	-
***Myriolimon ferulaceum* (L.) Lledó, Erben & M.B.Crespo**	F. Sales & I.C. Hedge 04/23 (B); Miquel A. Conesa 2009-29; M.M. Romeiras 411 (LISC)	MF083841	KJ608035	-	MH582658
Myriolimon diffusum (Pourr.) Lledó, Erben & M. B. Crespo	Sales & Hedge 04/15 (E)	MF083842	-	-	-
***Plumbagella micrantha* (Ledeb.) Spach**	Sino-American-British Yushu Expedition (1996) 2947 [E00061458] (E); CPG23229	MH561155	MH582836	KX526746	MH582660
***Plumbago auriculata* Lam.**	T. Schuster 1; M. J. Moore 306 (FLAS); K. Koutroumpa 1033 (Z)	JF831319	EU002283	MH583017	MH582665
***Plumbago caerulea* Kunth**	Marko Lewis 35396 [U.1487667] (U)	MH561152	MH582833	-	MH582663

***Plumbago europaea* L.**	ABH 16134 (ABH); A.M.W. Mennega 917 [U.1495156] (U); Crespo and Lledo, 7/10/95 (K); OPTIMA ITER V. 1026 (RNG)	AJ391334	MH582837	AY042634	HE602417
***Plumbago indica* L.**	K. Koutroumpa 1000 (Z)	MH561154	MH582835	-	MH582666
***Plumbago zeylanica* L.**	R. M. Harley 17219 [U.1487661] (U)	MH561153	MH582834	MH583016	MH582664
***Popoviolimon turcomanicum* (Popov ex Lincz.) Lincz.**	Faghihnia & Zanguei 28978 (FUMH)	MF083843	-	MF190371	JX983658
***Psylliostachys leptostachya* (Boiss.) Roshk.**	H. Akhani 22298 (Hb. Akh.)	MF083844	-	MF190448	MF041936
***Psylliostachys suvorovii* (Regel) Roshk.**	Chase 711 (K); K. Koutroumpa 1034 (Z); IS Bot.Gart.Innsbruck 1997#623	AJ391335	MW289906*	AY042639	AJ132446
***Psylliostachys spicata* (Willd.) Nevski**	Akhani 14878 (Hb. Akh.)	-	-	-	JX983656
***Saharanthus ifniensis* (Caball.) M.B.Crespo & M.D.Lledó**	Davis 53637 [E00770654] (E)	MW289873*	MW289905*	-	MW290505*
***Vassilczenkoa sogdiana* (Lincz.) Lincz.**	T.F. Hewer 1130 [E00013192] (E)	MH561143	MH582820	-	MH582647
**Outgroup Polygonaceae species**					
***Coccoloba diversifolia* Jacq.**	N. Swensen-202; Sanchez 102 (WFU)	-	HM446781	HM446674	HM137431
***Coccoloba swartzii* Meisn.**	Chase (NCU); R.A. Muscarella-143 (US); Chase 349 (NCU)	-	AF297150	KJ012533	FJ154469
***Coccoloba uvifera* (L.) L.**	R.A. Muscarella-466 (US); Kron s.n (WFU)	AJ312249	AF206753	KJ012536	GQ206246
***Eriogonum alatum* Torr.**	Reveal 8515 (WFU)	-	EF437977	-	FJ154472
***Fallopia convolvulus* (L.) Á.Löve**	Y.T. Hou036 (SDNU); OAC:JAG086; Won 391 (SNU)	EU024782	KM360782	EU749341	AF040064
***Fallopia dentatoalata* (F.Schmidt) Holub**	F.Z. Li4069 (SDNU); L.Q. Zhao 0891 (CPU); Won 162 (SNU)	EU024775	HM357888	EU024769	AF040066
***Fallopia dumetorum* (L.) Holub**	F.Z. Li03065 (SDNU); NMW2922 (NMW) [POWNA1055-12]; Park, C. & H.-W. Lee s.n. (SNU)	EU024785	JN890735	AM503813	AF040068
***Muehlenbeckia australis* (Forst.f.) Meisn.**	W.R. Barker 8995 & R.M. Barker (AD)	JF831303	FM883618	JF831267	JF831208
***Muehlenbeckia axillaris* (Hook.f.) Walp.**	K.L. Wilson 10562 (NSW); Chase 883 (K)	JF831304	-	AY042617	JF831209
***Muehlenbeckia complexa* (A.Cunn.) Meisn.**	K.L. Wilson 10677 (NSW); H. Schaefer 2008/357 (BM); Christensen s.n. (SNU)	JF831305	HM850184	HM851072	AF040076
***Muehlenbeckia costata* K.L.Wilson & Makinson**	J.J. Bruhl 2680 (NE)	JF831306	-	JF831269	JF831210
***Muehlenbeckia gracillima* Meisn.**	R. Johnstone 2022 & E.A. Orme (NSW)	JF831309	-	JF831237	JF831213

***Muehlenbeckia platyclada* (F.Muell.) Meisn.**	K.L. Wilson 10678 (NSW); FanDM-042; Costa, A. 688 (SNU)	JF831311	JN234979	JF831239	AF189738
***Muehlenbeckia rhyticarya* F.Muell.**	S.P. Phillips 1978 (BRI); Coverty & Jobson 17345 (SNU)	JF831312	-	-	AF189739
***Muehlenbeckia tiliifolia* Wedd.**	B. Øllgaard 87EC63051 (AAU)	JF831314	-	JF831270	JF831215
***Muehlenbeckia tuggeranong* Mallinson**	K.L. Wilson 10540 & D.J. Mallinson (ANBG)	JF831315	-	JF831271	JF831216
***Muehlenbeckia volcanica* (Benth.) Endl.**	M. Silman s.n. (WFU)	JF831317	-	JF831241	JF831218
***Neomillspaughia emarginata* (Gross) S.F.Blake**	Burke 66 (BH)	-	-	-	GQ206257
***Podopterus cordifolius* Rose & Standl.**	Burke 30 (BH)	-	FJ154455	FJ154494	FJ154479
***Triplaris americana* L.**	Chase 1337 (K); Silman s.n. (WFU)	AJ312251	Y16910	AY042668	FJ154486

Table S2 Divergence time estimates for major clades in the 'Supermatrix-ITS-like', 'Supermatrix-cpDNA-like', 'Supermatrix', ITS and cpDNA MCC trees, and for analyses with uniform or normal prior assigned to Plumbaginaceae stem-node (secondary calibration). Median ages and 95% HPD intervals in parenthesis for the crown-nodes of clades are in million years (Ma). Analyses with uniform prior for the secondary calibration are presented in the main text and used in all subsequent analyses.

**Uniform prior on secondary calibration for Plumbaginaceae stem-node**					
**Crown-nodes**	**Median ages (95% HPD)**				
	**'Supermatrix-ITS-like'**	**'Supermatrix-cpDNA-like'**	**'Supermatrix'**	**ITS**	**cpDNA**
Plumbaginaceae-Polygonaceae split (Root)	75.69 (69.03-78.21)	75.78 (69.18-78.21)	75.51 (68.78-78.21)	75.28 (67.33-78.21)	74.87 (66.52-78.21)
Plumbaginaceae	65.68 (52.01-77.03)	65.68 (52.07-77.2)	65.44 (52.44-77.05)	67.12 (54.4-77.53)	56.45 (41.37-74.58)
Plumbaginoideae	29.42 (18.71-42.76)	29.6 (18.36-43.23)	29.27 (17.87-42.39)	30.28 (17.98-43.98)	27.68 (15.02-43.74)
Limonioideae	56.92 (42.83-70.6)	57.2 (42.56-70.65)	56.52 (42.13-70.01)	-	49.25 (34.23-65.77)
Limonieae	40.35 (29.88-51.58)	40.1 (29.65-51.22)	40.51 (30.44-51.24)	46.28 (34.14-58.6)	34.31 (23.46-46.93)
Limonium	33.07 (23.68-44.23)	33.18 (23.39-43.76)	34.25 (24.97-44.63)	37.59 (25.68-50.13)	28.38 (18.57-39.39)
Limonium subg. *Pteroclados s.l.*	11.89 (6.25-19.97)	11.82 (6.2-19.45)	12.02 (5.94-20.49)	13.78 (6.44-24.04)	9.2 (4.03-16.72)
Limonium sect. *Pteroclados*	3.74 (2.11-5.86)	3.71 (2.11-5.82)	3.8 (2.11-6.09)	3.85 (2.08-6.36)	3.85 (1.83-6.75)
Limonium sect. *Pteroclados* subsect. *Nobiles*	2.32 (1.31-362)	2.29 (1.29-3.54)	2.35 (1.3-3.72)	2.95 (1.6-4.94)	2.07 (1.01-3.58)
Limonium subg. *Limonium*	21.46 (14.79-29.74)	21.12 (14.69-28.96)	21.79 (15.04-29.55)	25.37 (16.95-35.08)	18.35 (11.59-26.36)
Limonium clades B2-B3 split*	15.87 (11.11-21.52)	15.56 (11.12-21.12)	16.03 (11.29-21.52)	18.18 (12.2-24.9)	14.66 (9.5-20.61)
Limonium clade B2*	14.41 (10.03-19.57)	14.12 (9.89-19.05)	14.15 (10.05-18.99)	15.42 (10.16-21.22)	13.1 (8.25-18.65)
Limonium clade B3: 'Mediterranean lineage'*	12.39 (8.09-17.33)	12.23 (8.05-16.93)	9.15 (5.81-13.28)	12.68 (7.85-18.23)	12.82 (7.43-18.73)
Larger subclade of 'Mediterranean lineage'**	6 (3.72-8.99)	5.68 (3.41-8.46)	5.08 (3.09-7.52)	7.59 (4.43-11.69)	4.57 (2.43-7.57)
**Normal prior on secondary calibration for Plumbaginaceae stem-node**					
**Crown-nodes**	**Median ages (95% HPD)**				
	**'Supermatrix-ITS-like'**	**'Supermatrix-cpDNA-like'**	**'Supermatrix'**	**ITS**	**cpDNA**
Plumbaginaceae-Polygonaceae split (Root)	75.08 (65.9-84.93)	75.11 (65.61-84.82)	74.35 (65.06-83.58)	73.9 (63.99-83.86)	73.03 (61.08-86.59)
Plumbaginaceae	66.37 (50.78-80.11)	65.84 (50.49-80.37)	65.26 (50-78.86)	66.32 (51.58-80.07)	54.94 (38.98-74.48)
Plumbaginoideae	29.9 (18.05-44.49)	29.88 (18.14-43.21)	29.42 (17.97-42.74)	30.01 (18.19-44.42)	27.46 (14.59-44.49)
Limonioideae	57.76 (42.27-72.65)	57.22 (42.11-72.62)	56.39 (40.68-71.8)	-	48.05 (32.94-66.66)
Limonieae	40.52 (29.67-52.81)	40.41 (29.14-51.5)	40.6 (29.21-53.07)	45.98 (33.64-59.5)	33.73 (22.53-47.02)
Limonium	33.26 (23.52-44.7)	33.45 (23.68-43.93)	34.2 (23.58-45.78)	37.69 (24.8-50.87)	27.88 (17.73-39.88)
Limonium subg. *Pteroclados s.l.*	12.08 (5.83-19.63)	11.76 (5.77-19.25)	12.03 (5.7-20.72)	13.42 (6.14-23.31)	9.16 (3.63-17.33)
Limonium sect. *Pteroclados*	3.76 (2.09-5.94)	3.76 (2.11-5.95)	3.75 (2.06-6.12)	3.81 (1.99-6.28)	3.83 (1.82-6.83)
Limonium sect. *Pteroclados* subsect. *Nobiles*	2.32 (1.28-3.66)	2.32 (1.28-5.95)	2.35 (1.28-3.74)	2.92 (1.47-4.22)	2.04 (0.98-3.59)
Limonium subg. *Limonium*	21.58 (14.67-30.11)	21.32 (14.5-29.33)	21.74 (14.17-30.41)	25.18 (16.23-35.49)	18.05 (11.37-26.56)
Limonium clades B2-B3 split	15.94 (10.86-21.72)	15.85 (11.03-21.52)	16.02 (10.52-22.02)	17.78 (11.8-25.32)	14.34 (9.19-20.77)
Limonium clade B2*	14.51 (9.76-19.81)	14.37 (9.87-19.52)	14.17 (9.28-19.55)	15.11 (9.62-21.6)	13.26 (8.5-19.21)
Limonium clade B3: 'Mediterranean lineage'*	12.44 (7.98-17.59)	12.42 (8.13-17.61)	9.15 (5.63-13.59)	12.43 (7.54-18.51)	12.66 (7.36-18.92)
Larger subclade of 'Mediterranean lineage'**	6.07 (3.75-9.19)	6.03 (3.65-9.12)	5.06 (2.99-7.67)	7.47 (4.25-11.7)	4.52 (2.34-7.56)

* Coding of clades within *Limonium* follow Koutroumpa et al., (2018).

** The subclade has slightly different species composition between ITS and cpDNA datasets, and their respective Supermatrices, due to the incongruent position of some Aegean species (for details see Koutroumpa et al., 2018).

Table S3 Species distributions and assignments of species into the nine major areas for biogeographic analyses. The composition of countries for each of the nine major areas and a map are given below the table. CoL = Catalog of Life (Roskov et al., 2018).

Species	Distribution	Areas	Codes BioGeoBEARS	Distribution literature
L. aegaeum	Greece, Aegaean Isl., East Aegaean Isl. (Chios), W-Turkey (Anatolia)	EM	B	Brullo & Erben, (2016); CoL
L. albomarginatum	Greece (S-Peloponnisos: Gerolimenas)	EM	B	Brullo & Erben, (2016); CoL
L. algarvense	Baleares (Mallorca), Spain, Portugal, Morocco	EM, NA	B, C	African Plant Database; CoL
L. ammophilon	Greece (Aegaean Isl.: Amorgos, Donousa, Gyali, Milos, Syros), Crete, East Aegaean Isl. (Rhodos, Chios)	EM	B	Brullo & Erben, (2016); CoL
L. amopicum	Greece (Karpathos)	EM	B	Brullo & Erben, (2016); CoL
L. anatolicum	Turkey (Inner Anatolia)	IT	D	CoL
L. anthericoides	South Africa (W-Cape Prov.)	SA	A	African Plant Database; CoL
L. aphroditae	Greece (Kythira Isl.)	EM	B	Brullo & Erben, (2016); CoL
L. aragonense	Spain (Teruel)	EM	B	CoL
L. arboretum	Canary Isl. (Tenerife, La Palma Isl.)	MA	E	CoL
L. archaeothirae	Greece (Kyklades: Santorini)	EM	B	Brullo & Erben, (2016); CoL
L. articulatum	Corsica, Sardinia, Italy	EM	B	CoL
L. asparagoides	Algeria, Morocco	NA	C	CoL
L. aucheri	Greece (incl. Aegaean Isl.), Rhodos	EM	B	Brullo & Erben, (2016); CoL
L. aureum	Siberia (E Siberia), China (C Gansu, Nei Mongol, Ningxia, N Shaanxi, Shanxi), Mongolia	EA, IT	D, F	CoL; Flora of China Online,;GBIF; Virtual Guide to the Flora of Mongolia
L. auriculae-ursifolium	France, Spain, Portugal, Morocco, Algeria	EM, NA, CB	B, C, G	African Plant Database; CoL; GBIF
L. australe	Australia (Queensland, New South Wales, Victoria, Tasmania)	EA	F	CoL
L. avei	France, Sardinia, Sicily, Italy, Libya, Tunisia, Algeria, ?Egypt, Cyprus	EM, NA	B, C	CoL; EuroMed Checklist; www.tela-botanica.org
L. axillare	Bahrain, Egypt (SE-Egypt), Kuwait, Oman (Dhofar, Mascat & Oman), Saudi Arabia (C-Saudi Arabia, NE-Saudi Arabia, N-Saudi Arabia, Hejaz, Midyan, Rub al Khali, Asir),	AR	I	CoL; GBIF; Akhani et al., (2013)

	Sinai peninsula (S-Sinai), United Arab Emirates, Qatar, Yemen (Aden Desert, coastal Hadhramaut, Inner Hadhramaut, Tihama, W-Yemen), Pakistan (Karachi, Sind, Baluchistan), Somalia, Sudan, Eritrea			
L. beaumierianum	Algeria, Morocco, W-Sahara	NA	C	CoL; African Plant Database
L. bellidifolium	Turkey (Inner Anatolia, W-Anatolia, WN-Anatolia), Cyprus (E-Cyprus), East Aegaean Isl. (Lesvos), European Turkey, Iran (Iranian Aserbaijan), Spain, France, Corsica, Sardinia, Italy, Croatia, Greece (N-Aegaean region), Montenegro, Tunisia, E-England, Romania, Moldavia, European Russia, Crimea, Siberia (W-Siberia), Kazakhstan, Northern Caucasus	EM, NA, CB, IT	B, C, D, G	CoL
L. benmageci	Canary Isl. (Gran Canaria)	MA	E	CoL
L. biflorum	Baleares (Mallorca, Formentera, Menorca)	EM	B	CoL
L. binervosum	England, France, Ireland, Spain, Morocco, Portugal	EM, NA, CB	B, C, G	Euro Med Checklist; CoL
L. bocconei	NW-Sicily, Isole Egadi	EM	B	CoL
L. bollei	Canary Isl. (Islote de Lobos, Fuerteventura)	MA	E	CoL
L. bonduellei	Algeria, Morocco, Tunisia, Libya, Mauritania, Chad, Egypt	NA	C	CoL
L. bonifacience	Corsica	EM	B	CoL
L. bourgeaui	Canary Isl. (Lanzarote, Fuerteventura)	MA	E	CoL
L. brasiliense	Argentina (Buenos Aires, Chubut, Entre Rios, La Pampa, Mendoza, Neuquen, Rio Negro, Santa Cruz, Santa Fe), Brazil (Parana, Rio Grande do Sul, Santa Catarina), Uruguay (Rocha), SE-Brazil (Rio de Janeiro)	AM	H	CoL
L. brassicifolium	Canary Isl. (La Gomera)	MA	E	CoL

L. braunii	Cape Verde Isl. (Santo Antao Isl., Ilha de Sao Nicolau, Fogo Isl., Ilha Brava)	MA	E	CoL
L. brevipetiolatum	Greece (Ionian Isl.: Kerkyra, Lefkada, Kefalonia, Zakynthos, W-Peloponnisos)	EM	B	CoL
L. brunneri	Cape Verde Isl. (Sao Vicente Isl., Santa Luzia Isl., Sal Isl.)	MA	E	CoL
L. caesium	SE-Spain	EM	B	CoL
L. californicum	USA (California), Mexico (Baja California Norte, Baja California Sur)	AM	H	CoL
L. calliopsium	Greece (N-Crete (Rethimno))	EM	B	CoL
L. camposanum	Baleares (Mallorca)	EM	B	CoL
L. cancellatum	Albania, Croatia, Italy, Montenegro	EM	B	CoL
L. capense	South Africa	SA	A	African Plant Database; GBIF; CoL
L. carnosum	Azerbaijan (Nachichevan), Iran (NW-Iran, W-Iran)	IT	D	CoL
L. carolinianum	Canada (New Brunswick, Newfoundland, Nova Scotia, Prince Edward Isl., Quebec), USA (Alabama, Connecticut, Delaware, Florida, Georgia, Louisiana, Maine, Maryland, Mass, Mississippi, New Hampshire, New Jersey, New York, North Carolina, Rhode Island, South Carolina, Texas, Virginia), Bermuda	AM	H	CoL
L. carpathum	Greece (Karpathos)	EM	B	CoL
L. carpetanicum	C-Spain (Toledo)	EM	B	CoL
L. carthaginense	SE-Spain (Sierra de Minera)	EM	B	CoL
L. cephalonicum	Greece (Ionian Isl.: Kefalonia)	EM	B	CoL
L. cf pycnanthum	Turkey (Inner Anatolia)	IT	D	CoL
L. chersonesum	Greece (NE-Crete (Chersonissos Isl.))	EM	B	CoL; Brullo & Erben, (2016)
L. circaei	Italy	EM	B	CoL
L. compactum	Greece	EM	B	CoL; Brullo & Erben, (2016)

L. confusum	Baleares (Mallorca), Corsica, France, Italy, Sardinia, Portugal	EM	B	CoL; www.tela-botanica.org
L. connivens	Baleares (Mallorca)	EM	B	CoL
L. contortirameum	Corsica	EM	B	CoL
L. cordatum	SE-France, W-Italy	EM	B	CoL
L. cornarianum	Greece (SE-Crete (Moni Kapsa gorge))	EM	B	CoL; Brullo & Erben, (2016)
L. coronense	Greece (SW-Peloponnisos: Koroni)	EM	B	CoL; Brullo & Erben, (2016)
L. corsicum	Corsica	EM	B	CoL
L. cossonianum	Baleares (Mallorca), Spain, Morocco, Algeria	EM, NA	B, C	CoL
L. costae	C- & NE-Spain (Teruel to Urgel)	EM	B	CoL
L. cosyrense	Malta, Pantelleria, Sicily?	EM	B	CoL; EuroMed Checklist
L. crateriforme	Greece (NE-Crete, Karpathos, Kasos)	EM	B	CoL; Brullo & Erben, (2016)
L. creticum	Greece (SC-Crete)	EM	B	CoL; Brullo & Erben, (2016)
L. cumanum	?Corsica, Italy	EM	B	CoL
L. cylindrifolium	Saudi Arabia (Asir, Rub al Khali), Yemen (Aden Desert, coastal Hadhramaut, SW-Yemen, Tihama), NE-trop. Africa	AR	I	CoL
L. cymuliferum	Algeria, Morocco	NA	C	CoL
L. cythereum	Greece (Antikythira Isl., Kythira Isl.)	EM	B	CoL; Brullo & Erben, (2016)
L. daveaui	Portugal (Sa. Martinho do Porto - Seixal)	EM	B	CoL
L. delicatulum	SE-Spain, Tunisia, Algeria, Morocco, Libya	EM, NA	B, C	CoL
L. dendroides	Canary Isl. (La Gomera)	MA	E	CoL
L. densissimum	NE-Spain, France, Sicily, Italy, ?Sardinia	EM	B	CoL
L. dichotomum	C-Spain	EM	B	CoL
L. dichroanthum	China (W-Xinjiang), Kyrgyzstan, Kazakhstan	IT	D	CoL
L. dodartii	W-Portugal, W-Spain	EM	B	CoL
L. dufourii	E-Spain (Valencia, Castellon)	EM	B	CoL
L. ebusitanum	Baleares (Ibiza)	EM	B	CoL

L. echioides	Portugal, Spain, Baleares, France, Corsica, Sardinia, Sicily, Italy, Greece (widespread), Crete, Libya, Tunisia, Algeria, Morocco, Turkey (SSW-Anatolia, W-Anatolia), Cyprus (E-Cyprus, N-Cyprus, S-Cyprus), East Aegaean Isl. (Karpathos), Egypt (NW-coastal Egypt)	EM, NA	B, C	CoL
L. effusum	Turkey (SW-Anatolia, W-Anatolia)	EM	B	CoL
L. elaphonisicum	SW-Crete (Elafonisi Isl. and neighbouring coast)	EM	B	CoL; Brullo & Erben, (2016)
L. erectum	Spain (Guadalajara)	EM	B	CoL
L. estevei	Spain (between Mojacar and Carboneras)	EM	B	CoL
L. failachicum	Kuwait, S Iran (Kusestan)	AR	I	CoL
L. fallax	SW-Morocco (W-Sah)	NA	C	CoL
L. ferganense	Kyrgyzstan, Afghanistan (Parwan), Tajikistan, Uzbekistan	IT	D	CoL
L. flexuosum	Siberia (E-Siberia), China (NE-Nei Mongol), Mongolia	EA, IT	D, F	Flora of China Online; GBIF; CoL; Virtual Guide to the Flora of Mongolia
L. frederici	Greece (Dodecanese Isl.), N-Crete	EM	B	CoL; Brullo & Erben, (2016)
L. frutescens	Canary Isl. (Tenerife)	MA	E	CoL
L. furfuraceum	Spain (Alicante)	EM	B	CoL
L. gibertii	NE-Spain (Tarragona), Baleares (Ibiza, Mallorca)	EM	B	CoL
L. girardianum	S-France, NE-Spain, Baleares (Formentera)	EM	B	CoL
L. globuliferum	Turkey (Inner Anatolia), Syria (C-Syrian Desert, W-Syrian Mountains)	IT	D	CoL
L. gmelini	Slovakia, Hungary, Romania, Macedonia, Montenegro, Serbia & Kosovo, Bulgaria, Crimea, C- & E-European Russia, Ukraine, Moldavia, Siberia (W-Siberia, C-Siberia), Talysh, China (N-Xinjiang), Kazakhstan, Kyrgyzstan, Mongolia, Turkey (E-Anatolia, Inner Anatolia, N-Anatolia, SSW-	CB, EM, IT, AR	B, D, G, I	CoL; Akhani et al., (2013); Virtual Guide to the Flora of Mongolia

	Anatolia, SW-Anatolia, W-Anatolia, WN-Anatolia), European Turkey, Iran (E-Iran, N-Iran: Mts., NW-Iran: Iranian Aserbaijan, S-Iran, W-Iran: Mts.)			
L. gougetianum	Tunisia, Algeria, Baleares	EM, NA	B, C	CoL
L. grabusae	Greece (NW-Crete (Gramvousa Isl.))	EM	B	CoL; Brullo & Erben, (2016)
L. graecum	Greece (Kyklades)	EM	B	CoL; Brullo & Erben, (2016)
L. greuteri	Corsica	EM	B	CoL
L. guaicuru	Chile (Atacama, Coquimbo, Valparaiso)	AM	H	CoL
L. gueneri	Turkey (Antalya)	EM	B	CoL
L. gymnesicum	Baleares (Mallorca)	EM	B	CoL
L. haitiense	Haiti, Dominican Republic	AM	H	CoL
L. hibericum	NE-Spain	EM	B	CoL
L. hierapetrae	Greece (SE-Crete)	EM	B	CoL; Brullo & Erben, (2016)
L. hoeltzeri	Kyrgyzstan	IT	D	CoL
L. humile	England, Denmark, France, Germany, Ireland, Norway, Sweden, Spain, Portugal	CB, EM	B, G	CoL
L. hungaricum	Hungary, Romania, Slovakia	CB	G	CoL
L. hyblaeum	Sicily	EM	B	CoL
L. iconicum	Turkey (Inner Anatolia)	IT	D	CoL
L. imbricatum	Canary Isl. (Tenerife, La Palma Isl.)	MA	E	CoL
L. insigne	SE-Spain	EM	B	CoL
L. iranicum	Iran (EC-Iran, NE-Iran: Mts., S-Iran, W-Iran), Iraq (SE-Iraq: Mesopotamia)	IT, AR	D, I	CoL; Akhani et al., (2013)
L. jovibarba	Cape Verde Isl. (Sao Vicente Isl., Ilha de Sao Nicolau)	MA	E	CoL
L. kardamylii	Greece (S-Peloponnisos: between Kardamili and Ajios Nikolaos)	EM	B	CoL; Brullo & Erben, (2016)
L. kaschgaricum	China (SW-Xinjiang), Kyrgyzstan	IT	D	Flora of China (online); CoL
L. kraussianum	South Africa (W-Cape Prov.)	SA	A	CoL
L. lanceolatum	Morocco (Ma), Portugal (Lu)	EM, NA	B, C	Euro Med Checklist; CoL

L. latebracteatum	E-Spain	EM	B	CoL
L. latifolium	Bulgaria, Romania, C- & E-European Russia, Ukraine, Crimea, Caucasus, Moldavia	CB	G	CoL
L. laxiusculum	Portugal (Sintra)	EM	B	CoL
L. lilacinum	Turkey (Inner Anatolia)	IT	D	CoL
L. limbatum	USA (Arizona, New Mexico, Oklahoma, Texas), Mexico (Coahuila)	AM	H	CoL
L. lobatum	?Portugal, Spain, Greece (Aegina Isl.), Libya, Tunisia, Algeria, Morocco, NW-Sahara, Israel (coastal W-Israel, E-Israel: Rift Valley, N-Israel, N-Negev Desert, SC-Israel: Judean Desert), Egypt (NE-Egypt, NW-coastal Egypt), Iran (S-Iran, W-Iran: Mts.), Iraq (NE-Iraq, SE-Iraq: Mesopotamia, S-Iraq: Desert), Jordania (S-Jordania, W-Jordania), Kuwait, Saudi Arabia (C-Saudi Arabia, NE-Saudi Arabia, SW-Saudi Arabia: Asir), Sinai peninsula (Central Sinai), Syria (C-Syrian Desert, W-Syrian Mountains), Canary Isl. (Fuerteventura, Tenerife)	EM, NA, IT, AR, MA	B, C, D, E, I	Euro Med Checklist; CoL
L. lobinii	Cape Verde Isl.	MA	E	CoL
L. longebracteatum	C-Spain (Cuenca)	EM	B	CoL
L. lowei	Madeira (Porto Santo Isl.)	MA	E	CoL
L. macrophyllum	Canary Isl. (Tenerife)	MA	E	CoL
L. macropterum	Canary Isl. (Hierro)	MA	E	CoL
L. majus	Spain (Baza)	EM	B	CoL
L. meandrinum	Greece (SE-Karpathos)	EM	B	CoL
L. meyeri	Bulgaria, Crimea, E-European Russia, Armenia, Talysh, Turkmenistan, Ukraine, Turkey (E-Anatolia), Cyprus (E-Cyprus, N-Cyprus, S-Cyprus), Iran (EC-Iran,	CB, IT, EM	B, D, G	Euro Med Checklist; GBIF; CoL

	NE-Iran: Mts., N-Iran: Mts., NW-Iran: Iranian Aserbaijan, W-Iran: Mts.), Israel (coastal W-Israel, E-Israel: Rift Valley)			
L. milleri	Oman	AR	I	CoL
L. minoicum	Greece (SE-Crete (between Tsoutsouros and Tertsa))	EM	B	CoL
L. minutiflorum	Sicily	EM	B	CoL
L. minutum	Baleares (Mallorca)	EM	B	CoL
L. mouretii	Morocco	NA	C	CoL
L. mucronatum	SW-Morocco	NA	C	CoL
L. mucronulatum	Cyprus	EM	B	CoL
L. multiflorum	W-Portugal	EM	B	CoL
L. multiforme	Italy (Arcipelago Toscano, WC-Italy)	EM	B	CoL
L. narbonense	Turkey (S-Anatolia: Aleppo etc., SSW-Anatolia), Cyprus (E-Cyprus), Egypt (NE-Egypt, NW-coastal Egypt), Rhodos, European Turkey, Lebanon (coastal W-Lebanon), Sinai peninsula (Central Sinai, Northern Sinai), Syria (coastal W-Syria), Portugal, Spain, France, Corsica, Sardinia, Sicily, Italy, former Yugoslavia, Albania, Bulgaria, Greece, Aegaean Isl. (Chios, Donousa, Euboea, Kerkyra, Kos, Limnos, Milos, Naxos, Paros, Samos, Skiathos, Thirasia), Tunisia, Algeria, Morocco	EM, NA, AR, CB	B, C, G, I	Euro Med Checklist; CoL; Monica Moura Pers. Com.; Azorean vascular plants list
L. nudum	Iran (EC-Iran, N-Iran)	IT	D	CoL
L. nydeggeri	Portugal	EM	B	CoL
L. obtusifolium	Corsica	EM	B	CoL
L. ocymifolium	Greece (Kyklades)	EM	B	CoL
L. oligotrichum	Greece (E-Crete, Karpathos)	EM	B	CoL
L. otolepis	China (Gansu, N-Xinjiang), Afghanistan, Kazakhstan,	IT	D	CoL; Flora of China (online); Jstor

	Kyrgyzstan, Tajikistan, Uzbekistan, Turkmenistan			
L. ovalifolium	France, Spain, Portugal, Morocco	EM, NA, CB	B, C, G	CoL
L. palmyrense	Jordania (W-Jordania), Syria (C-Syrian Desert)	IT	D	CoL
L. papillatum	Islas Selvagens, Canary Isl. (Lanzarote, Fuerteventura)	MA	E	CoL
L. paulayanum	Socotra	AR	I	CoL
L. pectinatum* var. *corculum	Canary Isl. (Gran Canaria, Tenerife)	MA	E	CoL
L. pectinatum* var. *divaricatum	Canary Isl. (Tenerife)	MA	E	CoL
L. pectinatum* var. *solandri	Canary Isl. (Tenerife, La Gomera, Hierro, La Palma Isl.)	MA	E	CoL
L. peregrinum	South Africa (N-Cape Prov., W-Cape Prov.)	SA	A	African Plant Database; CoL
L. perezii	Canary Isl. (Tenerife)	MA	E	CoL
L. perfoliatum	Iran (E-Iran, NE-Iran: Mts., N-Iran, W-Iran), Afghanistan (Badakshan, Badghys, Baghlan, Herat, Jawzjan / Sar-e-Pol, Kabul, Qunduz, Samangan, Takhar), Turkmenistan, Tajikistan, Uzbekistan	IT	D	CoL
L. pigadiense	Greece (Karpathos)	EM	B	CoL
L. piptopodum	Tajikistan	IT	D	CoL
L. platyphyllum	Sweden, Netherlands, Finland, Great Britain, Moldova, The Russian Federation, Ukraine	CB	G	GBIF; Euro Med Checklist
L. plurisquamatum	Portugal (S. Martinho do Porto, Cabo Carvoeiro)	EM	B	CoL
L. preauxii	Canary Isl. (Gran Canaria)	MA	E	CoL
L. proliferum	Greece (C- and S-Aegaean region), East Aegaean Isl. (Chios, Rhodos)	EM	B	CoL
L. pruinosum	Israel (Rift Valley, N-Negev Desert, Judean Desert), Egypt (Eastern Desert, NE-Egypt, NW-coastal Egypt, SE-Egypt), Jordania (S-Jordania, W-Jordania), Kuwait, Saudi Arabia (Asir), Sinai Peninsula (C-Sinai, N-Sinai), Libya, Tunisia	IT, AR, NA	C, D, I	CoL; African Plant Database

L. pseudebusitanum	Baleares (Mallorca, Ibiza, Cabrera)	EM	B	CoL
L. puberulum	Canary Isl. (Lanzarote, Fuerteventura)	MA	E	CoL
L. purpuratum	South Africa (W-Cape Prov.)	SA	A	African Plant Database; CoL
L. pylium	Greece (W-Peloponnisos)	EM	B	CoL
L. recticaule	Greece (NE-Crete)	EM	B	CoL
L. recurvum* subsp. *humile	England, Ireland	EM	B	CoL; EuroMed Checklist
L. redivivum	Canary Isl. (La Gomera)	MA	E	CoL
L. relicticum	Canary Isl. (La Gomera)	MA	E	CoL
L. remotispiculum	SW-Italy	EM	B	CoL
L. reniforme	Iran (E-Iran, NE-Iran: Mts., N-Iran, W-Iran), Afghanistan (Badakshan, Badghys, Baghlan, Herat, Jawzjan / Sar-e-Pol, Kabul, Qunduz, Samangan, Takhar), Turkmenistan, Tajikistan, Uzbekistan	IT	D	CoL
L. rigualii	Spain (Denia - Calpe)	EM	B	CoL
L. roridum	Crete, Greece (C-Aegaean, S-Aegaean), Kyklades, Dodecanese, East Aegaean Isl. (Chios)	EM	B	CoL
L. santapolense	Spain (Santa Pola)	EM	B	CoL
L. saracinatum	Greece (Ithaki Isl., Kefalonia Isl., Lefkada Isl.)	EM	B	CoL
L. sarcophyllum	Oman	AR	I	CoL
L. scabrum	South Africa (W-Cape Prov., E-Cape Prov., N-Cape Prov.)	SA	A	African Plant Database; CoL
L. scopulorum	SE Spain	EM	B	CoL
L. sieberi	Greece (S-Peloponnisos, Aegaean Isl.: Kythira, Euboea, Alonisos), N-Crete, Turkey (SSW-Anatolia, W-Anatolia), Lebanon (coastal W-Lebanon), Syria (coastal W-Syria)	EM	B	CoL
L. sinense	China (Wet sandy and salty shales adjacent to the ocean: Fujian, Guangdong, Guangxi, Hebei, Jiangsu, Liaoning, Shandong,	EA	F	CoL; Flora of China (online)

	Zhejiang), Taiwan, Ryukyu Isl., Vietnam			
L. sinuatum	Estonia, Latvia, Lithuania, Moldavia, European Russia (widespread), Crimea, Portugal, Spain, France, Corsica, Sardinia, Sicily, Italy, former Yugoslavia, Albania, Greece, Aegaean Isl., Turkey (NW-Anatolia: Bithynia, S-Anatolia: Aleppo etc., SSW-Anatolia, SW-Anatolia, W-Anatolia), Cyprus (C-Mountains, E-Cyprus, N-Cyprus, W-Cyprus), E-Aegaean Isl. (Samos, Psara), Rhodos, Egypt (NE-Egypt, NW-coastal Egypt), Israel (coastal W-Israel), Lebanon (coastal W-Lebanon), Sinai peninsula (Central Sinai), Syria (coastal W-Syria), Canary Isl. (Fuerteventura, Gran Canaria, Tenerife, Lanzarote), Madeira	CB, EM, NA, AR, MA	B, C, E, G, I	Euro Med Checklist; CoL; http://www4.uma.pt/gbm/checklist/lista flora.php
L. sitiacum	Greece (NE-Crete (incl. Islands), Plati Isl. (near Kasos))	EM	B	CoL
L. sogdianum	Kazakhstan, Turkmenistan, Tajikistan, Uzbekistan	IT	D	CoL
L. sokotranum	Socotra, Samha Isl., Abd-al-Kuri Isl.	AR	I	CoL
L. somalorum	Somalia	AR	I	Flora of Somalia
L. sougiae	Greece (SW-Crete)	EM	B	CoL
L. sp1	Turkey (Inner Anatolia)	IT	D	CoL
L. sp2	Greece (Crete)	EM	B	CoL
L. spectabile	Canary Isl. (Tenerife)	MA	E	CoL
L. spreitzenhoferi	Greece (Kythira Isl.)	EM	B	CoL
L. stenotatum	Greece (Crete, Karpathos, Kasos)	EM	B	CoL
L. stocksii	Iran (S-Iran), NW-India, Pakistan (Karachi, Sind, Baluchistan)	AR	I	CoL
L. subglabrum	Spain (Mala)	EM	B	CoL

L. suffruticosum	Crimea, Ukraine, E-European Russia, Northern Caucasus, Azerbaijan, Siberia (W-Siberia), China (N-Xinjiang), Kazakhstan, Kyrgyzstan, Mongolia, Uzbekistan, Turkmenistan, Tajikistan, Iran (NE-Iran: Mts.), ?Jammu & Kashmir	CB, IT	D, G	CoL; Virtual Guide to the Flora of Mongolia
L. sundingii	Cape Verde Isl.	MA	E	CoL
L. supinum	SE-Spain (Almeria to Alicante)	EM	B	CoL
L. sventenii	Canary Isl. (Gran Canaria)	MA	E	CoL
L. tabernense	Spain (Sa. de Alhamilla)	EM	B	CoL
L. tamaricoides	Turkey (Inner Anatolia)	IT	D	CoL
L. tenellum	China (Nei Mongol, Ningxia), Mongolia	EA, IT	D, F	CoL; Virtual Guide to the Flora of Mongolia
L. tetragonum	New Caledonia (incl. Loyalty Isl.), South Korea, North Korea, Japan (Honshu, Shikoku, Kyushu), Ryukyu Isl.	EA	F	CoL
L. thiniense	Spain (Cartagena, Murcia, Petrola, Santa Pola)	EM	B	CoL
L. toletanum	C-Spain	EM	B	CoL
L. tomentellum	Crimea, Romania, Ukraine, E-European Russia, Northern Caucasus	CB	G	CoL
L. tournefortii	C- & NE-Spain	EM	B	CoL
L. tuberculatum	Canary Isl. (Gran Canaria, Islote de Lobos), Morocco, W-Sahara, Mauritania	MA, NA	C, E	CoL
L. tubiflorum	Libya, Egypt (Great Southwestern Desert, NW-coastal Egypt)	NA	C	CoL
L. tunetanum	Libya, Tunisia, Algeria	NA	C	CoL
L. vanandense	Greece (N-Karpathos)	EM	B	CoL
L. vigaroense	Canary Isl. (La Palma)	MA	E	CoL
L. virgatum	Portugal, Spain, Baleares, France, Corsica, Sardinia, Malta, Sicily, Italy, former Yugoslavia, Albania, Greece, Kyklades, Aegaean Isl., Libya, Tunisia, Algeria, Turkey	EM, NA, CB	B, C, G	CoL

	(NW-Anatolia: Bithynia, S-Anatolia: Aleppo etc., SSW-Anatolia, SW-Anatolia, W-Anatolia), Cyprus (E-Cyprus, N-Cyprus, S-Cyprus), E-Aegaean Isl. (Chios, Limnos, Lesbos, Samos, Rhodos), European Turkey, Israel (coastal W-Israel), Lebanon (coastal W-Lebanon), Syria (coastal W-Syria)			
L. vulgare	Belgium, England, Denmark, Germany, Netherlands, Romania, Sweden, Portugal, Spain, France	CB, EM	B, G	CoL; www.tela-botanica.org
L. vulgare eduardi diasii	Azores	MA	E	Schaefer et al., (2005)
L. wrightii	Taiwan, Japan (Izu Isl., Yakushima Isl.), Ryukyu Isl., Bonin Isl. (Keetaajima, Nakohdojima, Chichijima, Minamijima), Malesia (Batan Islands)	EA	F	CoL
L. xerocamposicum	Greece (E-Crete)	EM	B	CoL
L. xiliense	Greece (S-Peloponnisos: between Elea and Archangelos)	EM	B	CoL

(A) **SA: South Africa**= South Africa

(B) **EM: Euro-Mediterranean** = Greece, Spain, Portugal, Baleares, Corse, Italy, Sardegna, Cyprus, Sicily, Albania, Croatia, Montenegro, Malta, Pantelleria, France (South), Turkey (West part/European), Lebanon (W-coastal), Syria (W-coastal), Israel (coastal)

(C) **NA: North Africa** = Morocco, Algeria, W-Sahara, Tunisia, Libya, Egypt (excl. SE), Mauritania, Chad

(D) **IT: Irano-Turanian** (sensu Manafzadeh et al., 2017) = Inner Anatolia(Turkey), Armenia, Iran (excl. South; Kusestan & Baluchestan, The Persian Gulf and Gulf of Oman), Iraq (NE), Azerbaijan, Afghanistan, W- & N Jordan, Syria (Central & West Syrian Mountains), Israel (excl. coastal), Pakistan (north Baluchistan, Federally Administered tribal Area and south Khyber Pakhtunkhwa, and Gilgit Baltistan), India (only eastern Kashmir), Tajikistan, Uzbekistan, Turkmenistan, Kyrgyzstan, Kazakhstan, China (the western provinces of China: Xizang (Tibet), Xinjiang (Uyghur autonomous region), Qinghai (Kokonor, Bayan Har Shan), NW Gansu, northern Inner Mongolia, NW Sichuan), Mongolia (southern two thirds of the country)

(E) **MA: Macaronesia** (sensu Takhtajan, 1986) = Canary Islands, Cape Verde, Islas Selvagens, Azores, Madeira

(F) **EA: East Asia-Australia** = China (excl. the western provinces of China: see above in IT), E-Siberia, Mongolia (excl. southern two thirds of the country), New Caledonia, South Korea, North Korea, Japan, Ryukyu Isl., Taiwan, Vietnam, Bonin Isl., Malesia, Australia

(G) **CB: Circumboreal** = Hungary, Slovakia, Romania, Great Britain, France (excl. South), Ireland, Denmark, Germany, Ireland, Norway, Sweden, C- & E-European Russia, Ukraine, Serbia & Kosovo, Crimea, Caucasus, Moldavia, Sweden, Netherlands, Finland, The Russian Federation (C &W), W- & C-Siberia, Former Yugoslavia/North Macedonia, Bulgaria

(H) **AM: America** = Brazil, Argentina, Chile, Uruguay, USA, Mexico, Canada, Bermuda, Haiti, Dominican Republic.

(I) **AR: Arabia-NE Africa** = Somalia, Yemen, NE Trop. Africa, Saudi Aradia, Oman, Socotra, Samha Isl., Abd-al-Kuri Isl., Kuwait, Sinai, Bahrain, Qatar, United Arab Emirates, Erithrea, Sudan, Egypt (SE only), Pakistan (South), Iran (South part: Kusestan & Baluchestan, The Persian Gulf and Gulf of Oman), Iraq (SE), NW-India, S-Jordan

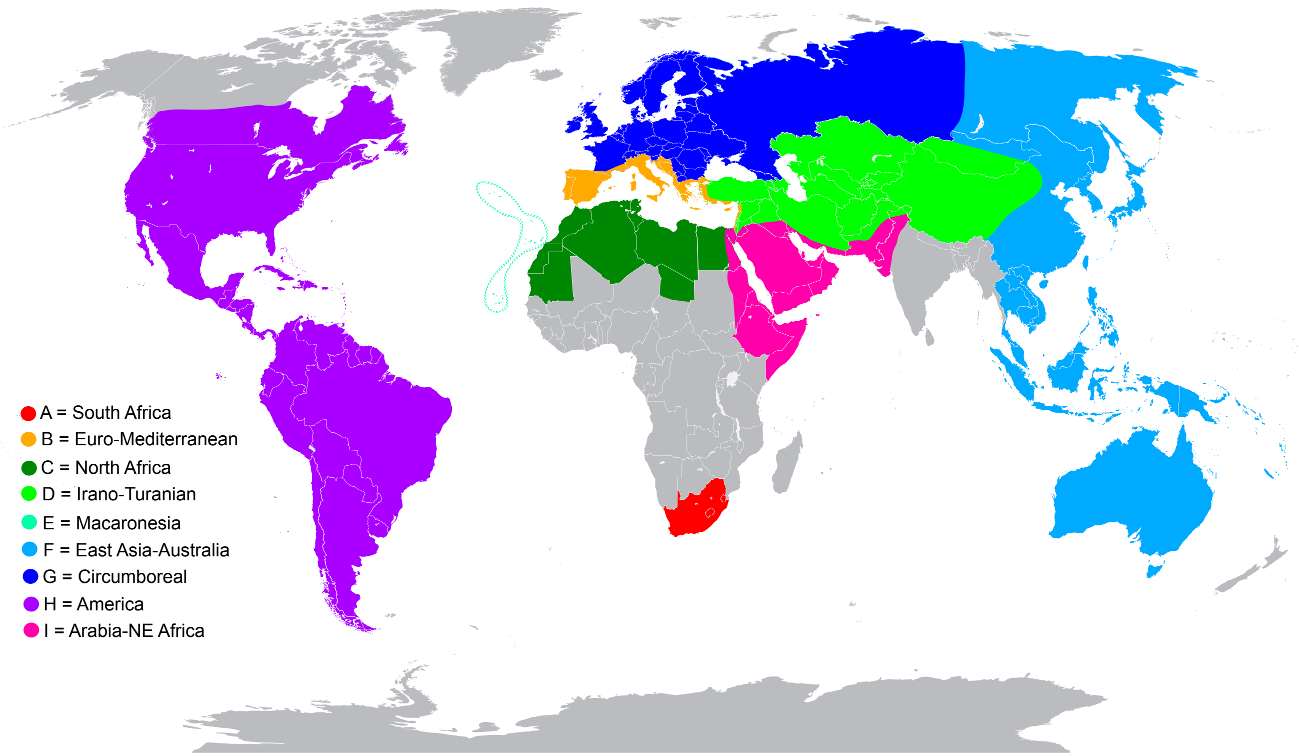
A = South Africa
B = Euro-Mediterranean
C = North Africa
D = Irano-Turanian
E = Macaronesia
F = East Asia-Australia
G = Circumboreal
H = America
I = Arabia-NE Africa

Table S4 Time-stratified dispersal multiplier matrices used in biogeographic analyses. Codes of the nine major areas are the same as in Table S3. We followed also paleomaps of: https://portal.gplates.org/map/

5.3 to 0 Mya: Present configuration and opening of the Bering Strait 3.5 Mya (Sanmartín et al., 2001), loss of connection between EA and AM

		SA	EM	NA	IT	MA	EA	CB	AM	AR
		**A**	**B**	**C**	**D**	**E**	**F**	**G**	**H**	**I**
**SA**	**A**	1	0.1	1	0.1	0.1	0.1	0.1	0.1	1
**EM**	**B**	0.1	1	0.5	1	0.1	0.1	1	0.1	1
**NA**	**C**	1	0.5	1	0.5	0.5	0.1	0.1	0.1	1
**IT**	**D**	0.1	1	0.5	1	0.1	1	1	0.1	1
**MA**	**E**	0.1	0.1	0.5	0.1	1	0.1	0.1	0.1	0.1
**EA**	**F**	0.1	0.1	0.1	1	0.1	1	1	0.5	0.5
**CB**	**G**	0.1	1	0.1	1	0.1	1	1	0.1	0.5
**AM**	**H**	0.1	0.1	0.1	0.1	0.1	0.5	0.1	1	0.1
**AR**	**I**	1	1	1	1	0.1	0.5	0.5	0.1	1

6 to 5.3 Mya: Messinian Salinity Crisis (Krijsman et al., 1999), connection of NA with EM

		SA	EM	NA	IT	MA	EA	CB	AM	AR
		**A**	**B**	**C**	**D**	**E**	**F**	**G**	**H**	**I**
**SA**	**A**	1	0.1	1	0.1	0.1	0.1	0.1	0.1	1
**EM**	**B**	0.1	1	1	1	0.1	0.1	1	0.1	1
**NA**	**C**	1	1	1	0.5	0.5	0.1	0.5	0.1	1
**IT**	**D**	0.1	1	0.5	1	0.1	1	1	0.1	1
**MA**	**E**	0.1	0.1	0.5	0.1	1	0.1	0.1	0.1	0.1
**EA**	**F**	0.1	0.1	0.1	1	0.1	1	1	1	0.5
**CB**	**G**	0.1	1	0.5	1	0.1	1	1	0.1	0.5
**AM**	**H**	0.1	0.1	0.1	0.1	0.1	1	0.1	1	0.1
**AR**	**I**	1	1	1	1	0.1	0.5	0.5	0.1	1

27 to 6 Mya: After the emergence of Macaronesia (Geldmacher et al., 2005; Fernandez-Palacios et al., 2011) and during the merge of the Arabian Peninsula with Europe (AR with IT and EM) (see also Ree & Sanmartín, 2009)

		SA	EM	NA	IT	MA	EA	CB	AM	AR
		**A**	**B**	**C**	**D**	**E**	**F**	**G**	**H**	**I**
**SA**	**A**	1	0.1	1	0.1	0.1	0.1	0.1	0.1	1
**EM**	**B**	0.1	1	0.5	1	0.1	0.1	1	0.1	1
**NA**	**C**	1	0.5	1	0.5	0.5	0.1	0.1	0.1	1
**IT**	**D**	0.1	1	0.5	1	0.1	1	1	0.1	1
**MA**	**E**	0.1	0.1	0.5	0.1	1	0.1	0.1	0.1	0.1
**EA**	**F**	0.1	0.1	0.1	1	0.1	1	1	1	0.5
**CB**	**G**	0.1	1	0.1	1	0.1	1	1	0.1	0.5
**AM**	**H**	0.1	0.1	0.1	0.1	0.1	1	0.1	1	0.1
**AR**	**I**	1	1	1	1	0.1	0.5	0.5	0.1	1

35 to 27 Mya: Represents the time before the emergence of Macaronesia when Arabia was still connected to Africa and not to Eurasia and AM-CB were closer (see also Meseguer et al.'s 2015 Supplementary material)

		SA	EM	NA	IT	MA	EA	CB	AM	AR
		**A**	**B**	**C**	**D**	**E**	**F**	**G**	**H**	**I**
**SA**	**A**	1	0.1	1	0.1	0	0.1	0.1	0.1	1
**EM**	**B**	0.1	1	0.5	1	0	0.1	1	0.1	0.5
**NA**	**C**	1	0.5	1	0.5	0	0.1	0.1	0.1	1
**IT**	**D**	0.1	1	0.5	1	0	1	1	0.1	0.5
**MA**	**E**	0	0	0	0	0	0	0	0	0
**EA**	**F**	0.1	0.1	0.1	1	0	1	1	1	0.1
**CB**	**G**	0.1	1	0.1	1	0	1	1	0.5	0.1
**AM**	**H**	0.1	0.1	0.1	0.1	0	1	0.5	1	0.1
**AR**	**I**	1	0.5	1	0.5	0	0.1	0.1	0.1	1

1: Immediately abutting areas or regions in the same continent separated only by 'grey' (see areas map; Table S3) and no other connecting area inbetween
0.5: Short-distance dispersal: short distances of water or relatively short distances of areas, and areas separated by another, only one, area.
0.1: Long-distance dispersal: substantial distance of water or areas, or both short distance of water and intermediate area(s).
0: Dispersal inhibited

Table S5 Sampling fractions per section/clade of *Limonium* following classifications of Koutroumpa et al. (2018; see Table S3 of this reference) for the two MCC trees used in BAMM analyses.

Sections/Clades	'Supermatrix-ITS-like' tree	'Supermatrix-cpDNA-like' tree
Circinaria	0.43	0.43
Iranolimon	0.75	0.75
Jovibarba-Ctenostachys	0.77	0.77
Limoniodendron	1	1
Limonium	0.67	0.67
**'Mediterranean lineage' (excl. *Polyarthrion, Pruinosum & Siphonantha*)**	0.25	0.27
Nephrophyllum s.l.	0.35	0.35
Plathymenium	0.41	0.41
Polyarthrion	0.67	0.67
Pruinosum	0.5	0.5
Pteroclados	1	1
Sarcophyllum	0.8	0.8
Siphonantha	0.25	0.25
Siphonocalyx s.l.	0.23	0.23
Sphaerostachys	1	1
Tenuiramosum	1	1

Table S6 *Limonium* species with their ploidy level, chromosome number in parenthesis, and their reproductive strategy.

Species	Ploidy (chromosome number)	Reproductive strategy	Literature
L. aegaeum	4x (34)	Apomictic	Brullo & Erben, (2016)
L. albomarginatum	5x (42)	Apomictic	Artelari & Georgiou, (2002a)
L. algarvense	3x (25)	Apomictic	Ingrouille, (1984); Llorens et al., (2018)
L. ammophilon	5x (42)	Apomictic	Brullo & Erben, (2016)
L. amopicum	5x (43)	Apomictic	Brullo & Erben, (2016)
L. anatolicum	2x (18)	Sexual	Erben, (1986); Özmen et al., (2012)
L. aphroditae	3x (27)	Apomictic	Artelari & Georgiou, (1999)
L. aragonense	2x (18)	Sexual	Erben, (1978); Ingrouille, (1984)
L. arboreum	2x (14)	Sexual	Febles & Pérez-Rodriguez, (2004); Baker, (1953)
L. archaeothirae	5x (43)	Apomictic	Brullo & Erben, (2016)
L. articulatum	2x (18)	Sexual	Arrigoni & Diana, (1993); Baker, (1953)
L. asparagoides	2x (18)	Sexual	Erben, (2001); Baker, (1953)
L. aucheri	5x (43)	Apomictic	Brullo & Erben, (2016)
L. aureum	2x (16)	Sexual	Zhang & Zheng, (2016); Baker, (1953)
L. auriculae-ursifolium	3x (25)	Apomictic	Ingrouille, (1984; 1985); Baker, (1966)
L. australe	?	Sexual	Baker, (1953; 1966)
L. avei	3x (27)	Apomictic	Kouzali et al., (2012)
L. axillare	?	Sexual	Baker, (1953)
L. bellidifolium	2x (18)	Sexual	several sources; Baker, (1953)
L. benmageci	?	Sexual	Perez de Paz et al., (2017)
L. biflorum	3x (25)	Apomictic	Llorens et al., (2018)
L. binervosum	4x (35)	Apomictic	Ingrouille (1984); Baker (1966)
L. bocconei	2x (18)	Sexual	Brullo & Pavone, (1981); Bogdanović & Brullo, (2015)
L. bollei	3x (24)	Apomictic	Febles & Pérez-Rodriguez, (2004); Perez de Paz et al., (2017)
L. bonduellei	2x (16)	Sexual	Darlington, (1955); Baker, (1953)
L. bonifacience	2x, 3x, 4x	Sexual	Arrigoni & Diana, (1993); Bogdanović & Brullo, (2015)

	(18, 27, 36)		
L. bourgeaui	2x (14)	Sexual	Febles & Pérez-Rodriguez, (2004); Baker, (1953)
L. brasiliense	?	Sexual	Baker, (1953)
L. brassicifolium	2x (14)	Sexual	Febles & Pérez-Rodriguez, (2004); Baker, (1953)
L. braunii	2x (12)	Sexual	Erben (1986); Baker, (1953)
L. brevipetiolatum	6x (54)	Sexual	Brullo & Erben (2016)
L. brunneri	2x (12)	Sexual	Erben, (1979; 1986); Baker, (1953)
L. caesium	2x (18)	Sexual	Erben, (1978); Baker, (1953)
L. californicum	2x (18)	Sexual	Raven, (1963); Baker, (1953; 1966)
L. camposanum	3x (26,27)	Apomictic	Llorens et al., (2018)
L. cancellatum	2x (18)	Sexual	Bogdanović et al., (2011); Bogdanović & Brullo, (2015)
L. carnosum	2x (18)	Sexual	Erben, (1986); Costa et al., (2019)
L. carolinianum	4x (36)	Sexual	www.efloras.org; Baker, (1953; 1966)
L. carpathum	4x (34)	Apomictic	Brullo & Erben, (2016)
L. carpetanicum	3x (25)	Apomictic	Erben, (1988; 1993); Crespo, (2009)
L. carthaginense	2x (18)	Sexual	Erben, (1978); Ingrouille, (1984)
L. cephalonicum	2x (18)	Sexual	Artelari, (1984)
L. circaei	2x (18)	Sexual	Guarino et al., (2017); Iberite et al., (2014)
L. confusum	3x (25)	Apomictic	Erben, (1978); Ingrouille, (1984)
L. connivens	3x (27)	Apomictic	Erben, (1989); Sáez & Rosello, (1999)
L. contortirameum	3x (27)	Apomictic	Arrigoni & Diana, (1993; 1999)
L. cordatum	2x (18)	Sexual	Rizzotto, (2003); Baker, (1953)
L. coronense	2x (18)	Sexual	Artelari, (1984)
L. corsicum	3x (27)	Apomictic	Erben, (1991; 2006); Paradis, (2009)
L. cossonianum	2x (16)	Sexual	Erben, (1978); Ingrouille, (1984)
L. costae	3x (26)	Apomictic	Erben, (1978); Ingrouille, (1984)
L. cosyrense	3x (27)	Apomictic	Brullo & Pavone, (1981); Baker, (1953)
L. crateriforme	4x (34)	Apomictic	Brullo & Erben, (2016)
L. creticum	6x (51)	Apomictic	Artelari (1989)
L. cumanum	2x (18)	Sexual	Brullo et al., (1990); Baker, (1953)
L. cylindrifolium	?	Sexual	Baker, (1953)
L. cymuliferum	2x (16)	Sexual	Erben, (1979)
L. cythereum	6x (52)	Apomictic	Artelari & Georgiou, (2002b)
L. delicatulum	3x (25)	Apomictic	Erben, (1978); Ingrouille, (1984)
L. dendroides	2x (18)	Sexual	Perez de Paz et al., (2017)
L. densissimum	3x (27)	Apomictic	Erben, (1978); Ingrouille, (1984)
L. dichotomum	2x (18)	Sexual	Erben, (1978); Ingrouille, (1984)
L. dodartii	4x (35)	Apomictic	Erben, (1978); Ingrouille, (1984)
L. dufourii	3x (26,27)	Apomictic	Castro & Rossello, (2007); Erben, (1978); Ingrouille, (1984)
L. ebusitanum	2x (18)	Sexual	Erben, (1988); Castro & Rossello, (2007)
L. echioides	2x (18)	Sexual	Erben, (1978); Baker, (1953)
L. effusum	?	Sexual	Baker, (1953)
L. erectum	2x (18)	Sexual	Erben, (1978); Ingrouille, (1984)
L. estevei	2x (16)	Sexual	Erben, (1978); Ingrouille, (1984)
L. failachicum	2x (18)	Sexual	Erben & Mucina, (2006); inference followed Erben, (1979)
L. flexuosum	?	Sexual	Baker, (1953)
L. frutescens	2x (14)	Sexual	Febles & Pérez-Rodriguez, (2004); Perez de Paz et al., (2017)
L. furfuraceum	2x (18)	Sexual	Erben, (1978); Ingrouille, (1984)
L. gibertii	3x (26,27)	Apomictic	Llorens et al., (2018)
L. girardianum	3x (26)	Apomictic	Erben, (1978); Ingrouille, (1984)
L. globuliferum	2x (18)	Sexual	Erben, (1986); Baker, (1953)
L. gmelini	2x (18)	Sexual	Borhidi (1968); Baker, (1953)
L. gougetianum	?	Apomictic	Baker, (1966)
L. graecum	6x (52)	Apomictic	Georgakopoulou et al., (2006)

L. greuteri	3x (27)	Apomictic	Erben, (2006); Paradis, (2009)
L. guaicuru	2x (18)	Sexual	Erben, (1986); Baker, (1953)
L. gymnesicum	3x (27)	Apomictic	Llorens *et al.,* (2018)
L. haitiense	?	Sexual	Baker, (1953; 1966)
L. hibericum	3x (27)	Apomictic	Erben (1988); Cowan et al., (1998)
L. hierapetrae	5x (43)	Apomictic	Artelari, (1989)
L. humile	6x (54)	Sexual	Erben, (1979); Ingrouille, (1984); Baker, (1966)
L. hyblaeum	4x (36)	Apomictic	Brullo & Pavone, (1981); Cowan et al., (1998)
L. iconicum	2x (18)	Sexual	Erben, (1986); Costa et al., (2019)
L. imbricatum	2x (14)	Sexual	Febles & Pérez-Rodriguez, (2004); Baker, (1953)
L. insigne	2x (18)	Sexual	Erben, (1978); Lledó et al., (2005)
L. iranicum	2x (18)	Sexual	Erben, (1986); Costa et al., (2019)
L. jovibarba	2x (12)	Sexual	Erben, (1986); Baker, (1953)
L. kardamylii	2x (18)	Sexual	Artelari & Kamari (1995)
L. kraussianum	?	Sexual	Baker, (1953)
L. lanceolatum	2x (16)	Sexual	Erben, (1999); Róis et al., (2016)
L. latebracteatum	3x (25)	Apomictic	Erben, (1978); Crespo, (2009)
L. latifolium	2x (18)	Sexual	Zhang & Zheng (2016); Baker, (1953)
L. lilacinum	4x (36)	Sexual	Evliyaoğlu et al., (2008); Costa et al., (2019)
L. limbatum	?	Sexual	Baker (1953; 1966)
L. lobatum	2x (12)	Sexual	Erben, (1978); Baker, (1953)
L. lobinii	2x (12)	Sexual	Kilian & Leyens, (1994); Malekmohammadi et al., (2017)
L. longebracteatum	3x (27)	Apomictic	Erben, (1988); inference followed Erben, (1979)
L. macrophyllum	2x (14)	Sexual	Borgen, (1970); Baker, (1953)
L. macropterum	2x (14)	Sexual	Larsen, (1963); Baker, (1953)
L. majus	3x (25)	Apomictic	Erben, (1978); Ingrouille, (1984)
L. meyeri	2x (18)	Sexual	Kouzali et al., (2012)
L. minoicum	5x (43)	Apomictic	Brullo & Erben, (2016)
L. minutiflorum	3x (26)	Apomictic	Brullo & Pavone, (1981); Costa et al., (2019)
L. minutum	2x (18)	Sexual	Llorens et al., (2018)
L. mouretii	2x (16)	Sexual	Erben, (1981)
L. mucronatum	2x (12)	Sexual	Erben, (1986); Baker, (1953)
L. mucronulatum	?	Apomictic	Kouzali et al., (2012)
L. multiflorum	4x (35)	Apomictic	Erben, (1978); Ingrouille, (1984)
L. multiforme	2x (18)	Sexual	Rizzotto, (1984); Brullo & Erben, (2016)
L. narbonense	4x (36)	Sexual	Georgakopoulou et al., (2006); Erben, (1978); Palop-Esteban *et al.*, (2011)
L. nudum	?	Sexual	Baker, (1953)
L. nydeggeri	2x (16)	Sexual	Erben, (1999); Róis et al., (2013)
L. obtusifolium	2x (18)	Sexual	Arrigoni & Diana, (1993); Paradis, (2009)
L. ocymifolium	5x (43)	Apomictic	Artelari, (1989); Artelari & Georgiou, (2003); Brullo & Erben (2016); Baker (1966)
L. otolepis	2x (18)	Sexual	Erben, (1986); Costa et al., (2019)
L. ovalifolium	2x (16)	Sexual	Erben, (1978); Ingrouille, (1984)
L. papillatum	2x (12)	Sexual	Borgen, (1970); Baker, (1953)
L. pectinatum var. *corculum*	2x (12)	Sexual	Erben, (1986); Baker, (1953)
L. pectinatum var. *divaricatum*	2x (12)	Sexual	Erben, (1986); Baker, (1953)
L. pectinatum var. *solandri*	2x (12)	Sexual	Erben, (1986); Baker, (1953)
L. perezii	2x (14)	Sexual	Borgen, (1970); Baker, (1953)
L. perfoliatum	?	Sexual	Baker, (1953)
L. pigadiense	5x (43)	Apomictic	Brullo & Erben, (2016)
L. plurisquamatum	3x (25)	Apomictic	Erben, (1978); Ingrouille, (1984)

L. preauxii	2x (14)	Sexual	Borgen, (1969); Baker, (1953)
L. proliferum	4x (34)	Apomictic	Brullo & Erben, (2016)
L. pruinosum	2x, 4x (16, 18, 32)	Sexual	Humphries, (1978); Brullo & Erben, (1989); Erben, (1979); Baker, (1953)
L. pseudebusitanum	2x (18)	Sexual	Erben, (1989); Castro & Rossello, (2007)
L. puberulum	2x (14)	Sexual	Erben, (1979); Baker, (1953)
L. cf. *pycnanthum*	?	Sexual	Özmen et al., (2012)
L. pylium	2x (18)	Sexual	Artelari, (1984)
L. recurvum subsp. *humile*	3x (27)	Apomictic	Ingrouille, (1984)
L. redivivum	2x (14)	Sexual	Febles & Pérez-Rodriguez, (2004); Perez de Paz et al., (2017)
L. remotispiculum	2x (18)	Sexual	Brullo et al., (1990); inference followed Erben, (1979)
L. reniforme	2x (18)	Sexual	Erben, (1986); Costa et al., (2019)
L. rigualii	3x (27)	Apomictic	Palacios et al., (2000); Sáez & Rosello, (1999)
L. roridum	5x (43)	Apomictic	Artelari & Georgiou, (2003); Brullo & Erben, (2016)
L. santapolense	3x (26)	Apomictic	Amorim et al., (2012); Erben, (1993); Crespo, (2009)
L. saracinatum	2x (18)	Sexual	Artelari, (1984)
L. scabrum	2x (18)	Sexual	Erben, (1986); Baker, (1953)
L. scopulorum	3x (25,26)	Apomictic	Castro & Rossello, (2007); Crespo (2009)
L. sieberi	5x (43)	Apomictic	Artelari & Georgiou, (2003); Brullo & Erben, (2016)
L. sinense	?	Sexual	Baker, (1953)
L. sinuatum	2x (16,18)	Sexual	Artelari, (1984); Erben, (1978); Loon & Snelders, (1979); Baker, (1953)
L. sitiacum	5x (43)	Apomictic	Brullo & Erben, (2016)
L. somalorum	?	Sexual	Baker, (1953)
L. spectabile	2x (14)	Sexual	Febles & Pérez-Rodriguez, (2004)
L. spreitzenhoferi	5x (43)	Apomictic	Brullo & Erben, (2016)
L. stenotatum	5x (42)	Apomictic	Brullo & Erben, (2016)
L. stocksii	?	Sexual	Baker, (1953)
L. subglabrum	3x (25)	Apomictic	Erben, (1978); Ingrouille, (1984)
L. suffruticosum	2x (18)	Sexual	Flora of China 15, www.efloras.org; Baker, (1953)
L. sundingii	2x (12)	Sexual	Lobin et al., (1995); Malekmohammadi et al., (2017)
L. supinum	3x (26)	Apomictic	Erben, (1978); Ingrouille, (1984)
L. sventenii	2x (14)	Sexual	Febles (1989); Perez de Paz et al., (2017)
L. tabernense	2x (16)	Sexual	Erben, (1978); Ingrouille, (1984)
L. tamaricoides	?	Sexual	Özmen et al., (2012)
L. tenellum	?	Sexual	Baker, (1953)
L. tetragonum	2x (16)	Sexual	Jino, (1953); Baker, (1966)
L. thiniense	3x (26)	Apomictic	Erben, (1981); Sáez & Rosello, (1999)
L. toletanum	2x (18)	Sexual	Erben, (1989); inference followed Erben, (1979)
L. tomentellum	4x (36)	Sexual	Aleskowsky, (1930); Baker, (1953)
L. tournefortii	3x (25)	Apomictic	Erben, (1978); Ingrouille, (1984)
L. tuberculatum	4x (32)	Sexual	Borgen, (1970); Baker, (1953)
L. tubiflorum	2x (18)	Sexual	Brullo et al., (1990); Baker, (1953)
L. tunetanum	2x (16)	Sexual	Brullo & Erben, (1989); inference followed Erben, (1979)
L. vanandense	7x (61)	Apomictic	Brullo & Erben, (2016)
L. virgatum	3x (27)	Apomictic	Erben, (1978); Ingrouille, (1984)
L. vulgare	4x (36)	Sexual	Erben, (1979); Baker, (1953)
L. wrightii	?	Sexual	Baker, (1953)

Table S7 Biogeographic models tested in this study, along with parameter estimates, log-likelihoods, AIC and AICc comparisons. M0 models include two time slices (35-27 and 27-0 Ma) to disallow Macaronesia as a possible ancestral range before 27 Ma, while M1 models additionally included time-stratified dispersal multiplier matrices with four time strata (35-27, 27-6, 6-5.3 and 5.3-0 Ma) to account for changes in distances and connectivity between areas over time. In bold is the best-fitting model for each dataset and its estimated parameters that were used for the Biogeographical Stochastic Mapping analyses.

'Supermatrix-ITS-like'	Log-likelihood	*d*	*e*	AIC	ΔAIC	AIC weight	AICc	ΔAICc	AICc weight
**M0: DEC**	**-329**	**0.036**	**0.016**	**662**	**0**	**1.000**	**662**	**0**	**1.000**
M0: DIVA-like	-362.6	0.044	0.020	729.1	67.1	0.000	729.2	67.2	0.000
M1: DEC	-337.9	0.053	0.016	679.7	17.7	0.000	679.8	17.8	0.000
M1: DIVA-like	-377.1	0.064	0.021	758.2	96.2	0.000	758.3	96.3	0.000
'Supermatrix-cpDNA-like'	Log-likelihood	*d*	*e*	AIC	ΔAIC	AIC weight	AICc	ΔAICc	AICc weight
**M0: DEC**	**-326.7**	**0.038**	**0.005**	**657.4**	**0**	**1.000**	**657.5**	**0**	**1.000**
M0: DIVA-like	-362.3	0.045	0.014	728.7	71.3	0.000	728.7	71.2	0.000
M1: DEC	-341.5	0.054	0.005	687.1	29.7	0.000	687.1	29.6	0.000
M1: DIVA-like	-378.2	0.064	0.016	760.5	103.1	0.000	760.5	103	0.000

d= rate of range expansion
e= rate of range contraction

Table S8 Number of dispersal events averaged across 200 Biogeographical Stochastic Maps (BSMs) with standard deviations in parentheses for analyses of **(A)** the 'Supermatrix-ITS-like' MCC tree and **(B)** the 'Supermatrix-cpDNA-like' MCC tree. Events with frequencies equal or above 0.9 are marker by bold numbers and are included in Fig. 2. Areas in rows represent source areas and areas in columns represent sink areas for dispersals. The sum and correspondent percentages of events for the areas acted as sources (rows) and sinks (columns) for dispersals are also provided in the last row and column, respectively. AM = America; AR = Arabia-NE Africa; CB = Circumboreal; EA = East Asia-Australia; EM = Euro-Mediterranean; IT = Irano-Turanian; MA = Macaronesia; NA = North Africa; SA = South Africa.

**(A)**	**SA**	**EM**	**NA**	**IT**	**MA**	**EA**	**CB**	**AM**	**AR**	**Total**
**SA**	-	0.24 (0.48)	0.30 (0.55)	0.19 (0.45)	0.25 (0.48)	0.02 (0.14)	0.14 (0.37)	0.02 (0.12)	0.32 (0.57)	1.48 2.19%
**EM**	0.39 (0.6)	-	**14,04** (1.63)	**1.75** (1.16)	**2.17** (1.23)	0.09 (0.3)	**5.58** (1.65)	0.22 (0.42)	**2.12** (1.22)	26.36 39.23%
**NA**	0.85 (0.73)	**1.73** (1.31)	-	**1.24** (1.02)	**2.47** (1.08)	0.05 (0.21)	**1.55** (1.14)	0.05 (0.21)	**1.78** (0.93)	9.71 14.45%
**IT**	0.24 (0.48)	**1.43** (1.04)	0.49 (0.65)	-	0.55 (0.76)	**1.40** (0.71)	**2.32** (0.96)	0.16 (0.39)	**3.37** (1.08)	9.96 14.82%
**MA**	0.07 (0.27)	0.43 (0.62)	**1.26** (0.96)	0.28 (0.51)	-	0.01 (0.07)	0.45 (0.69)	**1.35** (0.56)	0.34 (0.55)	4.19 6.23%
**EA**	0.01 (0.1)	0.05 (0.21)	0.03 (0.19)	**1.92** (0.6)	0.02 (0.14)	-	0.02 (0.12)	0.02 (0.12)	0.02 (0.12)	2.07 3.07%
**CB**	0.10 (0.31)	**2.18** (1.33)	**1.40** (0.96)	**0.94** (0.83)	0.54 (0.69)	0.07 (0.26)	-	0.46 (0.54)	0.74 (0.73)	6.43 9.56%
**AM**	0.01 (0.07)	0.16 (0.38)	0.02 (0.14)	0.04 (0.18)	0.56 (0.53)	0.01 (0.1)	0.15 (0.36)	-	0.02 (0.14)	0.96 1.43%
**AR**	**1.27** (0.83)	**0.92** (1.05)	0.88 (0.95)	**1.66** (1.2)	0.73 (0.77)	0.04 (0.18)	0.48 (0.69)	0.09 (0.3)	-	6.06 9.02%
**Total**	2.93 4.36%	7.14 10.62%	18.42 27.4%	8.02 11.93%	7.29 10.85%	1.68 2.49%	10.69 15.9%	2.35 3.5%	8.71 12.95%	67.2 100%

(B)	SA	EM	NA	IT	MA	EA	CB	AM	AR	Total
**SA**	-	0.26 (0.51)	0.30 (0.55)	0.34 (0.58)	0.31 (0.55)	0.00 (0.00)	0.18 (0.41)	0.00 (0.00)	0.30 (0.56)	1.69 2.67%
**EM**	0.53 (0.67)	-	**13.69** (1.81)	**1.48** (1.05)	**2.77** (1.03)	0.03 (0.16)	**4.97** (1.49)	0.30 (0.48)	**1.62** (1.05)	25.39 40.1%
**NA**	**0.90** (0.82)	**1.65** (1.18)	-	**0.98** (0.84)	**2.25** (1)	0.01 (0.07)	**1.66** (1.15)	0.29 (0.49)	**1.61** (0.84)	9.35 14.76%
**IT**	0.17 (0.38)	**1.48** (0.91)	0.60 (0.69)	-	0.28 (0.49)	**1.25** (0.5)	**2.21** (0.96)	0.04 (0.2)	**2.45** (0.63)	8.48 13.39%
**MA**	0.06 (0.24)	0.38 (0.56)	**1.38** (0.69)	0.29 (0.49)	-	0.00 (0.00)	0.20 (0.44)	**1.14** (0.46)	0.28 (0.49)	3.73 5.89%
**EA**	0.00 (0.00)	0.00 (0.00)	0.01 (0.07)	**1.93** (0.07)	0.01 (0.07)	-	0.01 (0.07)	0.00 (0.00)	0.01 (0.1)	1.96 3.09%
**CB**	0.09 (0.28)	**2.50** (1.09)	**1.28** (0.93)	**1.05** (0.07)	0.27 (0.5)	0.01 (0.07)	-	0.68 (0.7)	0.68 (0.74)	6.55 10.35%
**AM**	0.00 (0.00)	0.07 (0.26)	0.02 (0.14)	0.00 (0.5)	0.85 (0.39)	0.00 (0.00)	0.39 (0.49)	-	0.03 (0.17)	1.36 2.15%
**AR**	0.73 (0.73)	0.84 (0.82)	0.66 (0.77)	**1.23** (0.39)	0.76 (0.78)	0.01 (0.07)	0.41 (0.61)	0.18 (0.39)	-	4.82 7.61%
**Total**	2.48 3.91%	7.18 11.34%	17.94 28.33%	7.30 11.53%	7.50 11.84%	1.29 2.04%	10.03 15.83%	2.63 4.15%	6.98 11.03%	63.31 100%

Table S9 Biogeographical models tested for analyses of *Jovibarba-Ctenostachys* clade, along with parameter estimates, log-likelihoods, AIC and AICc comparisons. In bold is the best-fitting model for each dataset.

ITS	Log-likelihood	*d*	*e*	AIC	ΔAIC	AIC weight	AICc	ΔAICc	AICc weight
DEC	-20,4	0,046	0,000	44,7	5,3	0,066	46,0	5,3	0,066
**DIVA-like**	**-17,7**	**0,047**	0,000	**39,4**	**0,0**	**0,934**	**40,7**	**0,0**	**0,934**
BayArea-like	-29,7	0,092	0,3	63,3	23,9	0,000	64,6	23,9	0,000
cpDNA	Log-likelihood	*d*	*e*	AIC	ΔAIC	AIC weight	AICc	ΔAICc	AICc weight
DEC	-18,7	0,110	0,000	41,4	5,6	0,058	42,7	5,6	0,059
**DIVA-like**	**-15,9**	**0,140**	**0,000**	**35,8**	**0,0**	**0,941**	**37,1**	**0,0**	**0,941**
BayArea-like	-24,6	0,230	0,620	53,2	17,4	0,000	54,5	17,3	0,000
'Supermatrix'	Log-likelihood	*d*	*e*	AIC	ΔAIC	AIC weight	AICc	ΔAICc	AICc weight
DEC	-23	0,070	0,016	50,0	5,2	0,070	51,3	5,2	0,070
**DIVA-like**	**-20,4**	**0,080**	**0,000**	**44,8**	**0,0**	**0,930**	**46,2**	**0,0**	**0,930**
BayArea-like	-31,5	0,150	0,390	66,9	22,1	0,000	68,3	22,1	0,000

d= rate of range expansion

e= rate of range contraction

Table S10 Time and paleoenvironment-dependent diversification models fitted to the entire or parts of *Limonium* tree (RPANDA analyses) for (A) ‘Supermatrix-ITS-like’ and (B) ‘Supermatrix-cpDNA-like’ MCC trees. The best model based on AICc and Akaike weights (AICc w) is denoted in bold and black, while alternative models receiving some support (i.e. ΔAICc<2) are denoted in bold and grey, for each analysis.

(A)

**‘Supermatrix-ITS-like’**								
**Med_clade**	**LH**	**AICc**	**λ_0**	**α**	**μ_0**	**β**	**ΔAICc**	**AICc w**
PB const λ	-102.861	207.760	2.255		0.000		64.565	0.000
PB exp λ	-70.895	145.903	4.322	-0.867	0.000		2.708	0.044
BD const λ,μ	-71.464	147.041	8.579		7.924		3.846	0.025
**BD exp λ const μ**	**-68.488**	**143.204**	**7.027**	**-0.087**	**5.397**		**0.009**	**0.168**
**BD const λ exp μ**	**-68.483**	**143.195**	**7.145**		**5.707**	**0.077**	**0.000**	**0.169**
BD exp λ & μ	-68.482	145.349	7.109	-0.025	5.614	0.055	2.154	0.058
PB exp λ temp	-77.373	158.859	10.088	-0.883	0.000		15.664	0.000
**BD exp λ temp μ const**	**-68.991**	**144.211**	**8.040**	**-0.078**	**5.982**		**1.015**	**0.102**
**BD const λ exp μ temp**	**-68.729**	**143.687**	**7.311**		**5.483**	**0.079**	**0.492**	**0.132**
**BD exp λ temp exp μ temp**	**-68.176**	**144.736**	**4.973**	**0.412**	**4.382**	**0.447**	**1.541**	**0.078**
PB exp λ seaM	-86.490	177.092	0.554	-0.026	0.000		33.897	0.000
**BD exp λ seaM μ const**	**-69.459**	**145.147**	**6.167**	**-0.003**	**6.336**		**1.952**	**0.064**
**BD const λ exp μ seaM**	**-69.015**	**144.259**	**7.324**		**7.768**	**0.004**	**1.064**	**0.099**
BD exp λ seaM exp μ seaM	-68.409	145.203	15.300	0.011	16.288	0.015	2.008	0.062
**NonMed_tree**	**LH**	**AICc**	**λ_0**	**α**	**μ_0**	**β**	**ΔAICc**	**AICc w**
PB const λ	-241.859	485.758	0.468				136.275	0.000
PB exp λ	-185.719	375.562	1.228	-0.264			26.079	0.000
**BD const λ,μ**	**-172.680**	**349.483**	**3.116**		**3.095**		**0.000**	**0.319**
**BD exp λ const μ**	**-172.477**	**351.205**	**3.016**	**-0.002**	**2.945**		**1.721**	**0.135**
**BD const λ exp μ**	**-172.472**	**351.193**	**3.014**		**2.944**	**0.002**	**1.710**	**0.136**
BD exp λ & μ	-172.265	352.951	3.023	0.089	3.035	0.089	3.467	0.056
PB exp λ temp	-178.121	360.366	1.788	-0.449			10.883	0.001
**BD exp λ temp μ const**	**-172.572**	**351.394**	**3.023**	**-0.004**	**2.925**		**1.910**	**0.123**
**BD const λ exp μ temp**	**-172.545**	**351.340**	**2.993**		**2.892**	**0.005**	**1.857**	**0.126**
BD exp λ temp exp μ temp	-171.646	351.713	2.221	0.249	2.288	0.246	2.230	0.105
**Entire tree**	**LH**	**AICc**	**λ_0**	**α**	**μ_0**	**β**	**ΔAICc**	**AICc w**
PB const λ	-411.817	825.654	0.771				301.990	0.000
PB exp λ	-286.826	577.709	1.933	-0.364			54.046	0.000

BD const λ,μ	-260.235	524.529	4.989		4.945		0.865	0.160
BD exp λ const μ	-259.448	525.014	4.800	-0.002	4.665		1.350	0.125
BD const λ exp μ	-259.445	525.006	4.801		4.669	0.002	1.343	0.126
BD exp λ & μ	-259.478	527.151	4.743	-0.083	4.492	-0.083	3.488	0.043
PB exp λ temp	-278.592	561.243	4.217	-0.620			37.579	0.000
BD exp λ temp μ const	-259.289	524.696	4.724	-0.008	4.478		1.032	0.147
BD const λ exp μ temp	-259.247	524.611	4.683		4.448	0.007	0.948	0.153
**BD exp λ temp exp μ temp**	**-257.734**	**523.664**	**3.267**	**0.325**	**3.314**	**0.323**	**0.000**	**0.246**
**Reduced_Med_clade**	**LH**	**AICc**	**λ_0**	**α**	**μ_0**	**β**	**ΔAICc**	**AICc w**
PB const λ	-85.611	173.261	2.460				40.354	0.000
PB exp λ	-65.354	134.825	4.314	-0.847			1.918	0.054
BD const λ.μ	-64.835	133.787	7.701		6.715		0.880	0.091
BD exp λ const μ	-63.591	133.418	6.623	-0.101	4.811		0.511	0.109
BD const λ exp μ	-63.597	133.429	6.714		5.093	0.096	0.521	0.109
BD exp λ & μ	-63.582	135.560	6.433	-0.489	4.036	-0.505	2.653	0.037
PB exp λ temp	-71.214	146.545	9.369	-0.82483			13.638	0.000
BD exp λ temp μ const	-64.020	134.275	7.668	-0.078	5.521		1.368	0.071
BD const λ exp μ temp	-63.707	133.650	6.838		4.733	0.104	0.743	0.097
BD exp λ temp exp μ temp	-62.606	133.607	3.480	0.682	3.078	0.721	0.700	0.099
PB exp λ seaR	-70.000	144.117	0.802	-0.020			11.210	0.001
BD exp λ seaR μ const	-63.367	132.970	5.211	-0.004	4.381		0.063	0.137
**BD const λ exp μ seaR**	**-63.336**	**132.907**	**6.419**		**5.663**	**0.004**	**0.000**	**0.141**
BD exp λ seaR exp μ seaR	-63.230	134.856	5.563	-0.002	4.787	0.003	1.949	0.053

Notes: Macroevolutionary scenarios were compared in which speciation and extinction vary through time, or by paleo-temperature changes (temp) and past sea levels (sea) using Miller *et al.*'s (2005; seaM) or Rohling *et al.*'s (2014; seaR) datasets. Abbreviations: Med_clade: the Mediterranean subclade for which a shift is detected by BAMM analysis, and its slight reduced version to fit Rohling *et al.*'s dataset of past sea levels (Reduced_Med_clade); Non_Med_tree: the *Limonium* MCC tree excluding the clade with shift (Med_clade); PB: Pure birth; BD: Birth-death; const: constant rates; exp: exponentially varying rates; LH: log-likelihood; AICc: corrected Akaike Information Criterion; ΔAICc: the difference in AICc between the model with the lowest AICc and the others; AIC w: Akaike weight. Parameter estimates: λ_0 and μ_0: speciation and extinction, respectively, at present, or for a given environmental variable for which the values is 0; α and β: parameter controlling variation of speciation and extinction, respectively, with positive values meaning a positive effect of the environment on speciation or extinction (and conversely).

(B)

‘Supermatrix-cpDNA-like’								
**Med1_clade**	**LH**	**AICc**	**λ_0**	**α**	**μ_0**	**β**	**ΔAICc**	**AICc w**
PB const λ	-85,287	172,620	2,169				48,547	0,000
**PB exp λ**	**-59,968**	**124,073**	**4,054**	**-0,825**			**0,000**	**0,166**
BD const λ,μ	-62,003	128,143	7,610		6,966		4,070	0,022
**BD exp λ const μ**	**-58,983**	**124,242**	**5,957**	**-0,123**	**4,149**		**0,170**	**0,152**
**BD const λ exp μ**	**-59,085**	**124,445**	**6,200**		**4,709**	**0,097**	**0,372**	**0,138**
BD exp λ & μ	-58,912	126,289	5,656	-0,604	3,005	-0,631	2,217	0,055
PB exp λ temp	-64,951	134,038	8,907	-0,827			9,966	0,001
**BD exp λ temp μ const**	**-59,595**	**125,466**	**7,125**	**-0,099**	**4,907**		**1,394**	**0,083**
**BD const λ exp μ temp**	**-59,416**	**125,107**	**6,379**		**4,521**	**0,095**	**1,035**	**0,099**
BD exp λ temp exp μ temp	-59,293	127,051	5,386	0,189	4,232	0,256	2,979	0,037
PB exp λ seaM	-72,324	148,785	0,567	-0,025			24,712	0,000
**BD exp λ seaM μ const**	**-59,871**	**126,018**	**5,106**	**-0,004**	**5,328**		**1,946**	**0,063**
**BD const λ exp μ seaM**	**-59,283**	**124,842**	**6,295**		**6,896**	**0,005**	**0,770**	**0,113**
**BD exp λ seaM exp μ seaM**	**-58,648**	**125,761**	**14,041**	**0,012**	**15,249**	**0,016**	**1,689**	**0,071**
**Med2_clade**	**LH**	**AICc**	**λ_0**	**α**	**μ_0**	**β**	**ΔAICc**	**AICc w**
PB const λ	-2,555	7,310	4,888				6,190	0,015
**PB exp λ**	**1,756**	**1,120**	**9,185**	**-2,306**			**0,000**	**0,327**
BD const λ,μ	0,201	4,230	12,208		10,188		3,110	0,069
BD exp λ const μ	1,756	3,822	9,185	-2,306	0,000		2,702	0,085
BD const λ exp μ	2,018	3,297	8,066		2,131	2,283	2,177	0,110
BD exp λ & μ	2,126	6,100	8,160	-1,501	0,044	6,024	4,981	0,027
PB exp λ temp	-0,461	5,554	40,094	-1,582			4,434	0,036
BD exp λ temp μ const	0,413	6,507	18,868	-0,509	6,769		5,387	0,022
BD const λ exp μ temp	1,434	4,465	8,247		0,053	3,255	3,345	0,061
BD exp λ temp exp μ temp	1,584	7,185	3,169	0,930	0,196	2,742	6,065	0,016
PB exp λ seaM	-2,547	9,726	4,264	-0,002			8,606	0,004
BD exp λ seaM μ const	0,367	6,600	25,286	0,011	11,452		5,480	0,021
BD const λ exp μ seaM	0,254	6,826	12,663		5,232	-0,012	5,706	0,019
BD exp λ seaM exp μ seaM	-0,170	10,692	3,393	-0,018	0,311	-0,052	9,572	0,003

PB exp λ seaR	-1,647	7,926	3,100	-0,011			6,806	0,011
BD exp λ seaR μ const	0,366	6,601	9,389	-0,004	8,590		5,482	0,021
BD const λ exp μ seaR	2,127	3,080	9,430		19,224	0,038	1,960	0,123
BD exp λ seaMR exp μ seaR	2,249	5,855	12,873	0,005	23,046	0,036	4,735	0,031
**NonMeds_tree**	**LH**	**AICc**	**λ_0**	**α**	**μ_0**	**β**	**ΔAICc**	**AICc w**
PB const λ	-243,542	489,123	0,491				146,174	0,000
PB exp λ	-183,051	370,222	1,311	-0,279			27,273	0,000
**BD const λ,μ**	**-169,415**	**342,949**	**3,322**		**3,305**		**0,000**	**0,306**
**BD exp λ const μ**	**-169,195**	**344,633**	**3,215**	**-0,002**	**3,145**		**1,684**	**0,132**
**BD const λ exp μ**	**-169,190**	**344,622**	**3,212**		**3,143**	**0,002**	**1,673**	**0,133**
BD exp λ & μ	-168,982	346,372	3,204	0,079	3,213	0,079	3,423	0,055
PB exp λ temp	-175,700	355,519	2,015	-0,474			12,570	0,001
**BD exp λ temp μ const**	**-169,268**	**344,779**	**3,212**	**-0,005**	**3,105**		**1,830**	**0,123**
**BD const λ exp μ temp**	**-169,237**	**344,716**	**3,180**		**3,070**	**0,005**	**1,767**	**0,127**
**BD exp λ temp exp μ temp**	**-168,172**	**344,753**	**2,307**	**0,267**	**2,372**	**0,264**	**1,804**	**0,124**
**Entire tree**	**LH**	**AICc**	**λ_0**	**α**	**μ_0**	**β**	**ΔAICc**	**AICc w**
PB const λ	-411,723	825,465	0,813				321,972	0,000
PB exp λ	-277,807	559,670	2,074	-0,386			56,176	0,000
**BD const λ,μ**	**-250,564**	**505,185**	**5,290**		**5,250**		**1,692**	**0,134**
BD exp λ const μ	-249,725	505,563	5,097	-0,002	4,960		2,070	0,111
BD const λ exp μ	-249,722	505,558	5,099		4,965	0,002	2,065	0,112
BD exp λ & μ	-249,568	507,325	5,158	0,106	5,164	0,106	3,832	0,046
PB exp λ temp	-270,205	544,467	4,596	-0,644			40,974	0,000
**BD exp λ temp μ const**	**-249,505**	**505,123**	**5,013**	**-0,008**	**4,754**		**1,629**	**0,139**
**BD const λ exp μ temp**	**-249,458**	**505,030**	**4,969**		**4,721**	**0,008**	**1,536**	**0,145**
**BD exp λ temp exp μ temp**	**-247,652**	**503,493**	**3,374**	**0,347**	**3,420**	**0,346**	**0,000**	**0,313**
**Reduced_Med1_clade**	**LH**	**AICc**	**λ_0**	**α**	**μ_0**	**β**	**ΔAICc**	**AICc w**
PB const λ	-69,969	141,985	2,335				28,864	0,000
**PB exp λ**	**-54,488**	**113,122**	**3,970**	**-0,790**			**0,000**	**0,199**
BD const λ,μ	-56,345	116,834	6,545		5,555		3,713	0,031
**BD exp λ const μ**	**-53,965**	**114,224**	**5,138**	**-0,218**	**2,807**		**1,102**	**0,114**

**BD const λ exp μ**	**-53,902**	**114,097**	**5,283**		**3,391**	**0,184**	**0,976**	**0,122**
BD exp λ & μ	-53,896	116,286	5,378	0,084	3,671	0,247	3,165	0,041
PB exp λ temp	-58,700	121,545	8,042	-0,758			8,424	0,003
BD exp λ temp μ const	-54,680	115,652	6,710	-0,145	3,931		2,530	0,056
**BD const λ exp μ temp**	**-54,134**	**114,561**	**5,467**		**3,025**	**0,188**	**1,440**	**0,097**
BD exp λ temp exp μ temp	-53,447	115,388	3,374	0,512	2,596	0,597	2,266	0,064
PB exp λ seaR	-61,043	126,231	-0,014	1,597			13,109	0,000
BD exp λ seaR μ const	-54,937	116,166	4,693	-0,004	3,854		3,044	0,043
**BD const λ exp μ seaR**	**-53,584**	**113,462**	**5,046**		**4,327**	**0,008**	**0,340**	**0,168**
BD exp λ seaR exp μ seaR	-53,473	115,440	5,905	0,003	5,198	0,010	2,318	0,062

Notes: Macroevolutionary scenarios were compared in which speciation and extinction vary through time, or by paleo-temperature changes (temp) and past sea levels (sea) using Miller *et al.*'s (2005; seaM) or Rohling *et al.*'s (2014; seaR) datasets. Abbreviations: Med1_clade: the Mediterranean larger subclade for which a shift is detected by BAMM analysis, and its slight reduced version to fit Rohling *et al.*'s dataset of past sea levels (Reduced_Med1_clade); Med2_clade: the Mediterranean small subclade for which a shift is detected by BAMM analysis; Non_Med_tree: the *Limonium* MCC tree excluding the clade with shift (Med1&2_clade); PB: Pure birth; BD: Birth-death; const: constant rates; exp: exponentially varying rates; LH: log-likelihood; AICc: corrected Akaike Information Criterion; ΔAICc: the difference in AICc between the model with the lowest AICc and the others; AIC w: Akaike weight. Parameter estimates: λ_0 and μ_0: speciation and extinction, respectively, at present, or for a given environmental variable for which the values is 0; α and β: parameter controlling variation of speciation and extinction, respectively, with positive values meaning a positive effect of the environment on speciation or extinction (and conversely).

Table S11 The fit of alternative BiSSE models used to test the effect of breeding systems in diversification rates for *Limonium* under three sampling scenarios for analyses of (A) the 'Supermatrix-ITS-like' MCC tree and (B) the 'Supermatrix-cpDNA-like' MCC tree. The best model based on AIC and Akaike weights (w_i) is denoted in bold and black and alternative models receiving some support (i.e. ΔAIC<2) are denoted in bold and grey, for each analysis.

(A)

**Global Sampling Fraction (f=0.285)**					
**Model**	**np**	**lnLiK**	**AIC**	**ΔAIC**	w_i
**Full BiSSE: all free**	**6**	**-306 .668**	**625 .336**	**1 .974**	**0 .182**
BiSSE (pure-birth) μ=0	4	-375 .619	759 .237	135 .876	0 .000
BiSSE (pure-birth) μ=0, λ=equal	3	-452 .276	910 .552	287 .191	0 .000
BiSSE λ=equal, μ= equal, q=equal	3	-331 .080	668 .160	44 .799	0 .000
**BiSSE λ=equal**	**5**	**-307 .074**	**624 .148**	**0 .787**	**0 .330**
BiSSE λ=equal, μ=equal	4	-320 .709	649 .418	26 .056	0 .000
BiSSE λ=equal, q=equal	4	-328 .282	664 .563	41 .202	0 .000
**BiSSE μ=equal**	**5**	**-306 .681**	**623 .361**	**0 .000**	**0 .488**
BiSSE μ=equal, q=equal	4	-329 .344	666 .687	43 .326	0 .000
BiSSE q=equal	5	-321 .392	652 .785	29 .423	0 .000
**Sampling Fractions assuming all apomicts being in the 'Mediterranean lineage' with 50-50% sexuals vs. apomicts (make.bisse.uneven)**					
**Model**	**np**	**lnLiK**	**AIC**	**ΔAIC**	w_i
**Full BiSSE: all free**	**6**	**-307 .97**	**627 .94**	**1 .882**	**0 .264**
BiSSE (pure-birth) μ=0	4	-360 .092	728 .183	102 .124	0 .000
BiSSE (pure-birth) μ=0, λ=equal	3	-456 .835	919 .669	293 .61	0 .000
BiSSE λ=equal, μ= equal, q=equal	3	-347 .343	700 .686	74 .627	0 .000
BiSSE λ=equal	5	-310 .491	630 .982	4 .923	0 .058
BiSSE λ=equal, μ=equal	4	-332 .549	673 .097	47 .039	0 .000
BiSSE λ=equal, q=equal	4	-344 .991	697 .982	71 .923	0 .000
**BiSSE μ=equal**	**5**	**-308 .029**	**626 .059**	**0 .000**	**0 .678**
BiSSE μ=equal, q=equal	4	-346 .117	700 .235	74 .176	0 .000
BiSSE q=equal	5	-325 .371	660 .743	34 .684	0 .000
**Sampling Fractions assuming all apomicts being in the 'Mediterranean lineage' with 40-60% sexuals vs. apomicts (make.bisse.uneven)**					
**Model**	**np**	**lnLiK**	**AIC**	**ΔAIC**	w_i
**Full BiSSE: all free**	**6**	**-307 .167**	**626 .3337**	**0 .394**	**0 .445**
BiSSE (pure-birth) μ=0	4	-361 .376	730 .7528	104 .813	0 .000
BiSSE (pure-birth) μ=0, λ=equal	3	-459 .595	925 .1892	299 .25	0 .000
BiSSE λ=equal, μ= equal, q=equal	3	-347 .705	701 .4097	75 .47	0 .000
BiSSE λ=equal	5	-311 .687	633 .3748	7 .435	0 .013
BiSSE λ=equal, μ=equal	4	-332 .213	672 .4257	46 .486	0 .000
BiSSE λ=equal, q=equal	4	-346 .414	700 .8282	74 .889	0 .000
**BiSSE μ=equal**	**5**	**-307 .97**	**625 .9395**	**0 .000**	**0 .542**
BiSSE μ=equal, q=equal	4	-347 .228	702 .4561	76 .517	0 .000
BiSSE q=equal	5	-320 .507	651 .0133	25 .074	0 .000

(B)

Global Sampling Fraction (f=0.292)					
**Model**	**np**	**lnLiK**	**AIC**	**ΔAIC**	***w*i**
Full BiSSE: all free	6	-291.790	595.581	1.856	0.192
BiSSE (pure-birth) μ=0	4	-373.115	754.230	160.504	0.000
BiSSE (pure-birth) μ=0, λ=equal	3	-447.121	900.242	306.517	0.000
BiSSE λ=equal, μ= equal, q=equal	3	-309.891	625.782	32.057	0.000
BiSSE λ=equal	5	-292.280	594.559	0.834	0.321
BiSSE λ=equal, μ=equal	4	-301.420	610.840	17.114	0.000
BiSSE λ=equal, q=equal	4	-308.187	624.374	30.649	0.000
**BiSSE μ=equal**	**5**	**-291.863**	**593.725**	**0.000**	**0.487**
BiSSE μ=equal, q=equal	4	-308.850	625.700	31.975	0.000
BiSSE q=equal	5	-301.681	613.362	19.637	0.000
**Sampling Fractions assuming all apomicts being in the 'Mediterranean lineage' with 50-50% sexuals vs. apomicts (make.bisse.uneven)**					
**Model**	**np**	**lnLiK**	**AIC**	**ΔAIC**	***w*i**
Full BiSSE: all free	6	-295.000	602.000	0.955	0.338
BiSSE (pure-birth) μ=0	4	-358.040	724.079	123.035	0.000
BiSSE (pure-birth) μ=0, λ=equal	3	-451.716	909.432	308.387	0.000
BiSSE λ=equal, μ= equal, q=equal	3	-324.620	655.241	54.196	0.000
BiSSE λ=equal	5	-297.060	604.119	3.074	0.117
BiSSE λ=equal, μ=equal	4	-312.334	632.668	31.623	0.000
BiSSE λ=equal, q=equal	4	-322.681	653.361	52.317	0.000
**BiSSE μ=equal**	**5**	**-295.522**	**601.045**	**0.000**	**0.545**
BiSSE μ=equal, q=equal	4	-323.583	655.166	54.121	0.000
BiSSE q=equal	5	-309.276	628.552	27.507	0.000
**Sampling Fractions assuming all apomicts being in the 'Mediterranean lineage' with 40-60% sexuals vs. apomicts (make.bisse.uneven)**					
**Model**	**np**	**lnLiK**	**AIC**	**ΔAIC**	***w*i**
**Full BiSSE: all free**	**6**	**-293.910**	**599.821**	**0.000**	**0.966**
BiSSE (pure-birth) μ=0	4	-358.786	725.573	125.752	0.000
BiSSE (pure-birth) μ=0, λ=equal	3	-454.159	914.319	314.498	0.000
BiSSE λ=equal, μ= equal, q=equal	3	-324.284	654.568	54.747	0.000
BiSSE λ=equal	5	-298.324	606.648	6.828	0.032
BiSSE λ=equal, μ=equal	4	-312.903	633.806	33.985	0.000
BiSSE λ=equal, q=equal	4	-323.298	654.596	54.776	0.000
BiSSE μ=equal	5	-300.819	611.639	11.818	0.003
BiSSE μ=equal, q=equal	4	-323.952	655.905	56.084	0.000
BiSSE q=equal	5	-305.216	620.432	20.612	0.000

np= number of free parameters
λ= speciation rates
μ= extinction rates
q= transition rates

Table S12 The fit of alternative HiSSE models used to test the effect of breeding systems in diversification rates for *Limonium* under three sampling scenarios for analyses of (A) the 'Supermatrix-ITS-like' MCC tree and (B) the 'Supermatrix-cpDNA-like' MCC tree. The best model based on AIC and Akaike weights (w_i) is denoted in bold. For details on the tested HiSSE models see Beaulieu & O'Meara (2016).

(A)

**Global Sampling Fraction (f=0.285)**					
**Model**	**np**	**lnLiK**	**AIC**	**ΔAIC**	w_i
**BiSSE: all free**	**6**	**-306.0905**	**624.181**	**0**	**0.847**
CID-2: q's equal	5	-325.0304	660.0608	35.8798	0.000
CID-2: three q rates (A<->B, 0->1, 1->0)	7	-315.51	645.02	20.839	0.000
CID-4: q's equal	9	-323.1761	664.3522	40.1712	0.000
CID-4: three q rates (A<->B, 0->1, 1->0)	11	-313.7011	649.4021	25.2211	0.000
HiSSE: q's equal	9	-316.6829	651.3658	27.1848	0.000
HiSSE: three q rates (A<->B, 0->1, 1->0)	11	-302.7992	627.5984	3.4174	0.153
**Sampling Fractions assuming all apomicts being in the 'Mediterranean lineage' with 50-50% sexuals vs. apomicts (f_{sex}=0.295, f_{ap}=0.269)**					
**Model**	**np**	**lnLiK**	**AIC**	**ΔAIC**	w_i
BiSSE: all free	**6**	-305.8426	623.6852	5.6263	0.057
CID-2: q's equal	4	-325.1532	660.3064	42.2475	0.000
CID-2: three q rates (A<->B, 0->1, 1->0)	3	-314.7588	643.5175	25.4586	0.000
CID-4: q's equal	3	-345.5722	709.1444	91.0855	0.000
CID-4: three q rates (A<->B, 0->1, 1->0)	5	-312.578	647.1559	29.097	0.000
HiSSE: q's equal	4	-343.2212	704.4423	86.3834	0.000
**HiSSE: three q rates (A<->B, 0->1, 1->0)**	**4**	**-298.0295**	**618.0589**	**0**	**0.943**
**Sampling Fractions assuming all apomicts being in the 'Mediterranean lineage' with 40-60% sexuals vs. apomicts (f_{sex}=0.335, f_{ap}=0.224)**					
**Model**	**np**	**lnLiK**	**AIC**	**ΔAIC**	w_i
BiSSE: all free	**6**	-305.8741	623.7483	7.4049	0.024
CID-2: q's equal	4	-323.4999	656.9998	40.6564	0.000
CID-2: three q rates (A<->B, 0->1, 1->0)	3	-314.1677	642.3354	25.992	0.000
CID-4: q's equal	3	-326.3625	670.725	54.3816	0.000
CID-4: three q rates (A<->B, 0->1, 1->0)	5	-313.2896	648.5792	32.2358	0.000
HiSSE: q's equal	4	-338.9052	695.8105	79.4671	0.000
**HiSSE: three q rates (A<->B, 0->1, 1->0)**	**4**	**-297.1717**	**616.3434**	**0**	**0.976**

(B)

**Global Sampling Fraction (f=0.292)**					
**Model**	**np**	**lnLiK**	**AIC**	**ΔAIC**	w_i
BiSSE: all free	6	-296.4395	604.8791	11.7616	0.003
CID-2: q's equal	5	-307.2312	624.4624	31.3449	0.000
CID-2: three q rates (A<->B, 0->1, 1->0)	7	-298.9529	611.9058	18.7883	0.000
CID-4: q's equal	9	-306.6533	631.3066	38.1891	0.000
CID-4: three q rates (A<->B, 0->1, 1->0)	11	-293.124	608.2479	15.1304	0.001
HiSSE: q's equal	9	-292.4508	602.9017	9.7842	0.007
**HiSSE: three q rates (A<->B, 0->1, 1->0)**	**11**	**-285.5588**	**593.1175**	**0**	**0.989**
**Sampling Fractions assuming all apomicts being in the 'Mediterranean lineage' with 50-50% sexuals vs. apomicts (f_{sex}=0.295, f_{ap}=0.287)**					
**Model**	**np**	**lnLiK**	**AIC**	**ΔAIC**	w_i
**BiSSE: all free**	**6**	**-295.138**	**602.276**	**0**	**0.829**
CID-2: q's equal	4	-306.9386	623.8771	21.6011	0.000
CID-2: three q rates (A<->B, 0->1, 1->0)	3	-298.9545	611.909	9.633	0.007
CID-4: q's equal	3	-308.1733	634.3466	32.0706	0.000
CID-4: three q rates (A<->B, 0->1, 1->0)	5	-293.1345	608.2689	5.9929	0.041
HiSSE: q's equal	4	-294.0539	606.1079	3.8319	0.122
HiSSE: three q rates (A<->B, 0->1, 1->0)	4	-296.7257	615.4514	13.1754	0.001
**Sampling Fractions assuming all apomicts being in the 'Mediterranean lineage' with 40-60% sexuals vs. apomicts (f_{sex}=0.335, f_{ap}=0.239)**					
**Model**	**np**	**lnLiK**	**AIC**	**ΔAIC**	w_i
BiSSE: all free	**6**	-295.5589	603.1177	19.8557	0.000
CID-2: q's equal	4	-306.2843	622.5685	39.3065	0.000
CID-2: three q rates (A<->B, 0->1, 1->0)	3	-299.8791	613.7581	30.4961	0.000
CID-4: q's equal	3	-307.8211	633.6421	50.3801	0.000
CID-4: three q rates (A<->B, 0->1, 1->0)	5	-299.2257	620.4514	37.1894	0.000
HiSSE: q's equal	4	-294.6381	607.2761	24.0141	0.000
**HiSSE: three q rates (A<->B, 0->1, 1->0)**	**4**	**-280.631**	**583.262**	**0**	**1.000**

Table S13 The fit of alternative GeoHiSSE models used to test the effect of Euro-Mediterranean range in diversification rates for *Limonium* for analyses of the 'Supermatrix-ITS-like' and the 'Supermatrix-cpDNA-like' MCC trees. The best model based on AIC and Akaike weights (w_i) is denoted in bold. Non EuroMed = species occurring in areas other than the Euro-Mediterranean; EuroMed = species occurring only in the Euro-Mediterranean area; Widespread = species occurring in the Euro-Mediterranean and other areas.

**'Supermatrix-ITS-like' tree (Sampling Fractions: Non EuroMed=0.43, EuroMed=0.27, Widespread=0.73)**					
**Model**	**np**	**lnLiK**	**AIC**	**ΔAIC**	$\boldsymbol{w_i}$
Dispersal parameters vary only, no range-dependent diversification	4	-380.3436	768.6872	52.5748	0.000
Canonical GeoSSE model, range effect on diversification	7	-369.9559	753.9118	37.7994	0.000
**GeoHiSSE model with 1 hidden area, no range-dependent diversification**	**9**	**-349.0562**	**716.1124**	**0**	**0.999**
GeoHiSSE model with 1 hidden area, range effect on diversification	15	-349.7447	729.4895	13.3771	0.001
**'Supermatrix-cpDNA-like' tree (Sampling Fractions: Non EuroMed=0.43, EuroMed=0.29, Widespread=0.73)**					
**Model**	**np**	**lnLiK**	**AIC**	**ΔAIC**	$\boldsymbol{w_i}$
Dispersal parameters vary only, no range-dependent diversification	4	-376.7726	761.5452	61.5833	0.000
Canonical GeoSSE model, range effect on diversification	7	-365.7881	745.5762	45.6143	0.000
**GeoHiSSE model with 1 hidden area, no range-dependent diversification**	**9**	**-340.981**	**699.9619**	**0**	**0.956**
GeoHiSSE model with 1 hidden area, range effect on diversification	15	-338.0692	706.1383	6.1764	0.044

Table S14 The fit of alternative BiSSE models used to test the effect of woodiness in diversification rates for *L.* subg. *Pteroclados s.l.* clade in the 'Supermatrix-ITS-like' and the 'Supermatrix-cpDNA-like' MCC trees. The best model based on AIC and Akaike weights (w_i) is denoted in bold and black and alternative models receiving some support (i.e. ΔAIC<2) are denoted in bold and grey, for each analysis.

**'Supermatrix-ITS-like' tree**					
**Model**	**np**	**lnLiK**	**AIC**	**ΔAIC**	w_i
Full BiSSE: all free	6	-26.89856	65.79711	3.02334	0.067
BiSSE (pure-birth) μ=0	4	-30.57016	69.14032	6.36655	0.013
BiSSE (pure-birth) μ=0, λ=equal	3	-38.5233	83.04659	20.27282	0.000
**BiSSE λ=equal, μ= equal, q=equal**	**3**	**-28.38688**	**62.77377**	**0**	**0.302**
BiSSE λ=equal	5	-28.27111	66.54221	3.76844	0.046
**BiSSE λ=equal, μ=equal**	**4**	**-28.20378**	**64.40756**	**1.63379**	**0.133**
**BiSSE λ=equal, q=equal**	**4**	**-28.3783**	**64.75661**	**1.98284**	**0.112**
BiSSE μ=equal	5	-28.04241	66.08483	3.31106	0.058
BiSSE μ=equal, q=equal	4	-28.38689	64.77377	2	0.111
**BiSSE q=equal**	**5**	**-27.03318**	**64.06635**	**1.29258**	**0.158**
**'Supermatrix-cpDNA-like' tree**					
**Model**	**np**	**lnLiK**	**AIC**	**ΔAIC**	w_i
Full BiSSE: all free	6	-26.68836	65.37673	3.05535	0.066
BiSSE (pure-birth) μ=0	4	-30.28363	68.56726	6.24588	0.013
BiSSE (pure-birth) μ=0, λ=equal	3	-38.32209	82.64417	20.32279	0.000
**BiSSE λ=equal, μ= equal, q=equal**	**3**	**-28.16069**	**62.32138**	**0**	**0.303**
BiSSE λ=equal	5	-28.04072	66.08145	3.76007	0.046
**BiSSE λ=equal, μ=equal**	**4**	**-27.9803**	**63.9606**	**1.63922**	**0.133**
**BiSSE λ=equal, q=equal**	**4**	**-28.15181**	**64.30362**	**1.98224**	**0.112**
BiSSE μ=equal	5	-27.81082	65.62164	3.30026	0.058
**BiSSE μ=equal, q=equal**	**4**	**-28.16069**	**64.32137**	**1.99999**	**0.111**
**BiSSE q=equal**	**5**	**-26.82294**	**63.64588**	**1.3245**	**0.156**

Table S15 The fit of alternative HiSSE models used to test the effect of woodiness in diversification rates for *L.* subg. *Pteroclados s.l.* clade in the 'Supermatrix-ITS-like' and the 'Supermatrix-cpDNA-like' MCC trees. The best model based on AIC and Akaike weights (w_i) is denoted in bold and black and alternative models receiving some support (i.e., ΔAIC<2) are denoted in bold and grey, for each analysis.

**'Supermatrix-ITS-like' tree**					
**Model**	**np**	**lnLiK**	**AIC**	**ΔAIC**	w_i
**BiSSE: all free**	6	**-27.06391**	**66.12782**	**0.3065**	**0.398**
**CID-2: q's equal**	**5**	**-27.91066**	**65.82132**	**0**	**0.464**
CID-2: three q rates (A<->B, 0->1, 1->0)	7	-27.3067	68.6134	2.79208	0.115
CID-4: q's equal	9	-27.90879	73.81759	7.99627	0.009
CID-4: three q rates (A<->B, 0->1, 1->0)	11	-27.48363	76.96726	11.14594	0.002
HiSSE: q's equal	9	-27.72045	73.4409	7.61958	0.010
HiSSE: three q rates (A<->B, 0->1, 1->0)	11	-27.08375	76.16749	10.34617	0.003
**'Supermatrix-cpDNA-like' tree**					
**Model**	**np**	**lnLiK**	**AIC**	**ΔAIC**	w_i
**BiSSE: all free**	6	**-27.29883**	**66.59767**	**1.16648**	**0.299**
**CID-2: q's equal**	**5**	**-27.7156**	**65.43119**	**0**	**0.536**
CID-2: three q rates (A<->B, 0->1, 1->0)	7	-27.08602	68.17204	2.74085	0.136
CID-4: q's equal	9	-28.23532	73.3317	7.90051	0.010
CID-4: three q rates (A<->B, 0->1, 1->0)	11	-27.27517	76.55035	11.11916	0.002
HiSSE: q's equal	9	-27.47841	72.95681	7.52562	0.012
HiSSE: three q rates (A<->B, 0->1, 1->0)	11	-26.78321	75.56643	10.13524	0.003

Figure S1 Time calibrated phylogeny based on 'Supermatrix-ITS-like' dataset (MCC tree), with median ages on nodes and 95% HPD interval bars. Support values are denoted by black circles for posterior probabilities ≥ 0.95, grey circles for posterior probabilities ≥ 0.75 and < 0.90, and white circles for posterior probabilities < 0.75. Red squares on nodes denote the placement of fossil calibrations and green square denotes the placement of the secondary calibration. Colored tips denote three 'rogue clades' identified by RAxML and MrBayes analyses as incongruent between ITS and cpDNA trees. For the six 'rogue taxa' see Koutroumpa et al. (2018: Figure 3).

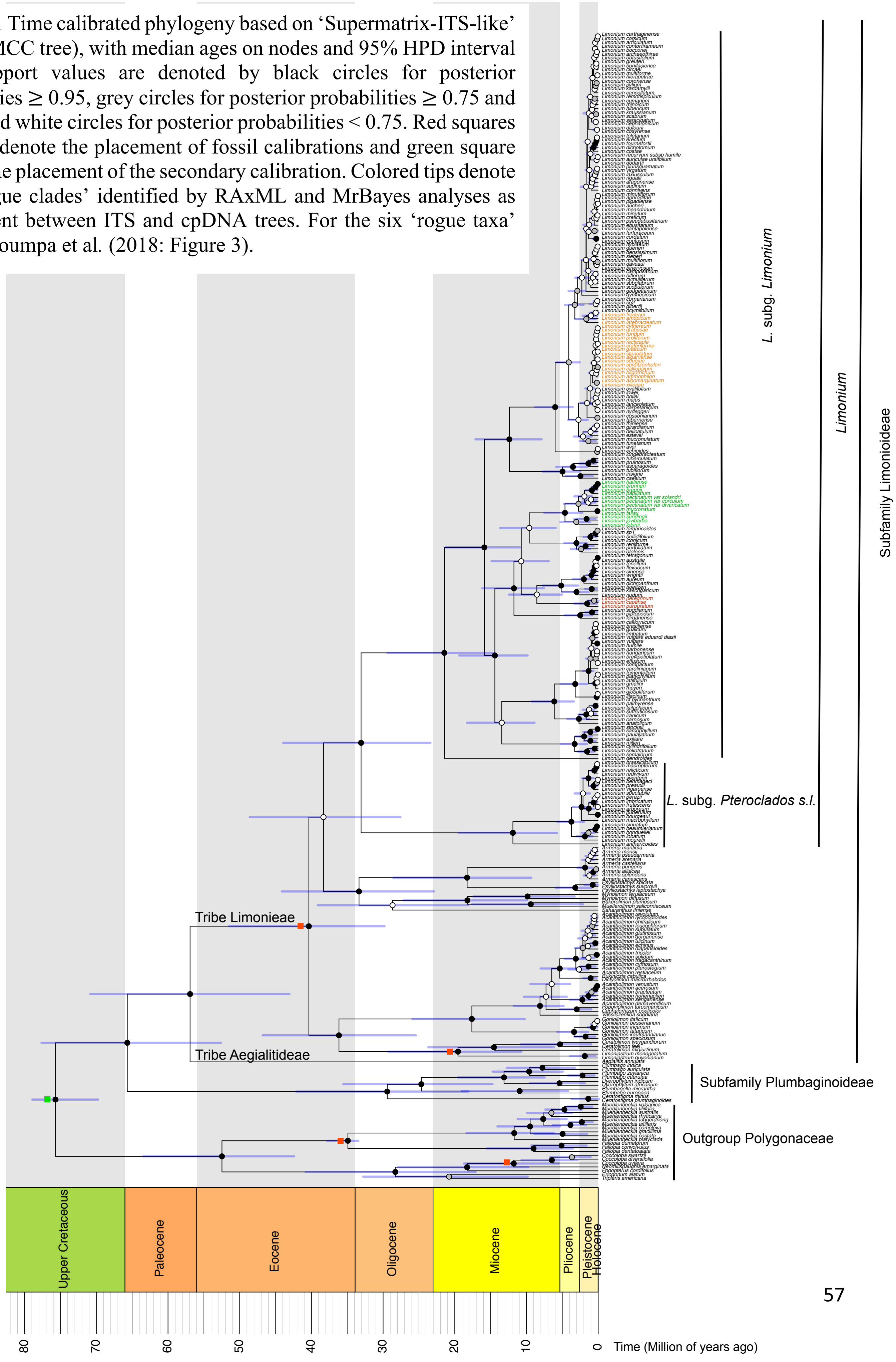

Figure S2 Time calibrated phylogeny based on 'Supermatrix-cpDNA-like' dataset (MCC tree), with median ages on nodes and 95% HPD interval bars. Support values are denoted by black circles for posterior probabilities ≥ 0.95, grey circles for posterior probabilities ≥ 0.75 and < 0.90, and white circles for posterior probabilities < 0.75. Red squares on nodes denote the placement of fossil calibrations and green square denotes the placement of the secondary calibration. Colored tips denote three 'rogue clades' identified by RAxML and MrBayes analyses as incongruent between ITS and cpDNA trees. For the six 'rogue taxa' see Koutroumpa et al. (2018: Figure 3).

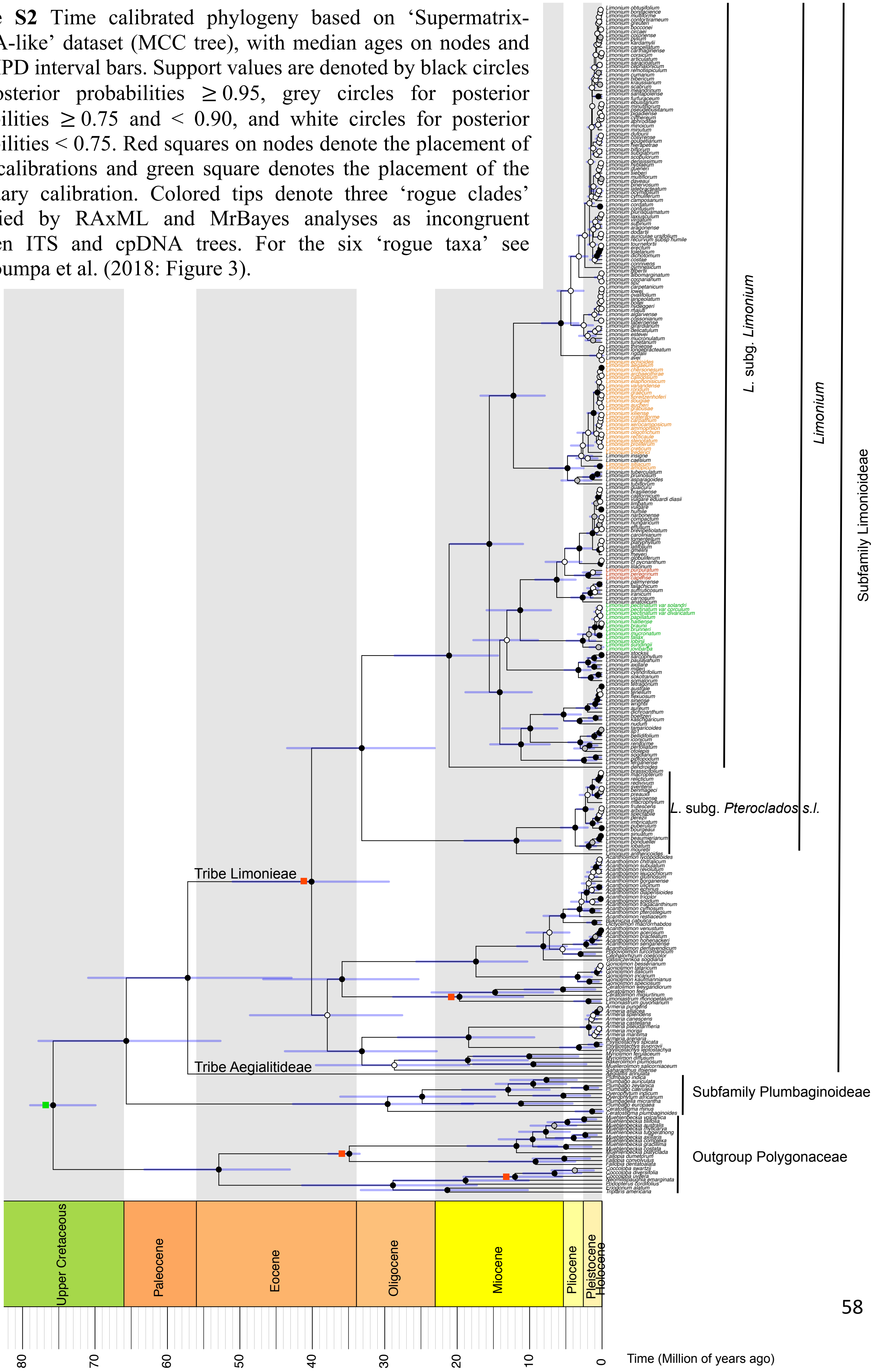

Figure S3 Environmental variables through time and their curves used in RPANDA paleo-environment dependent diversification analyses.

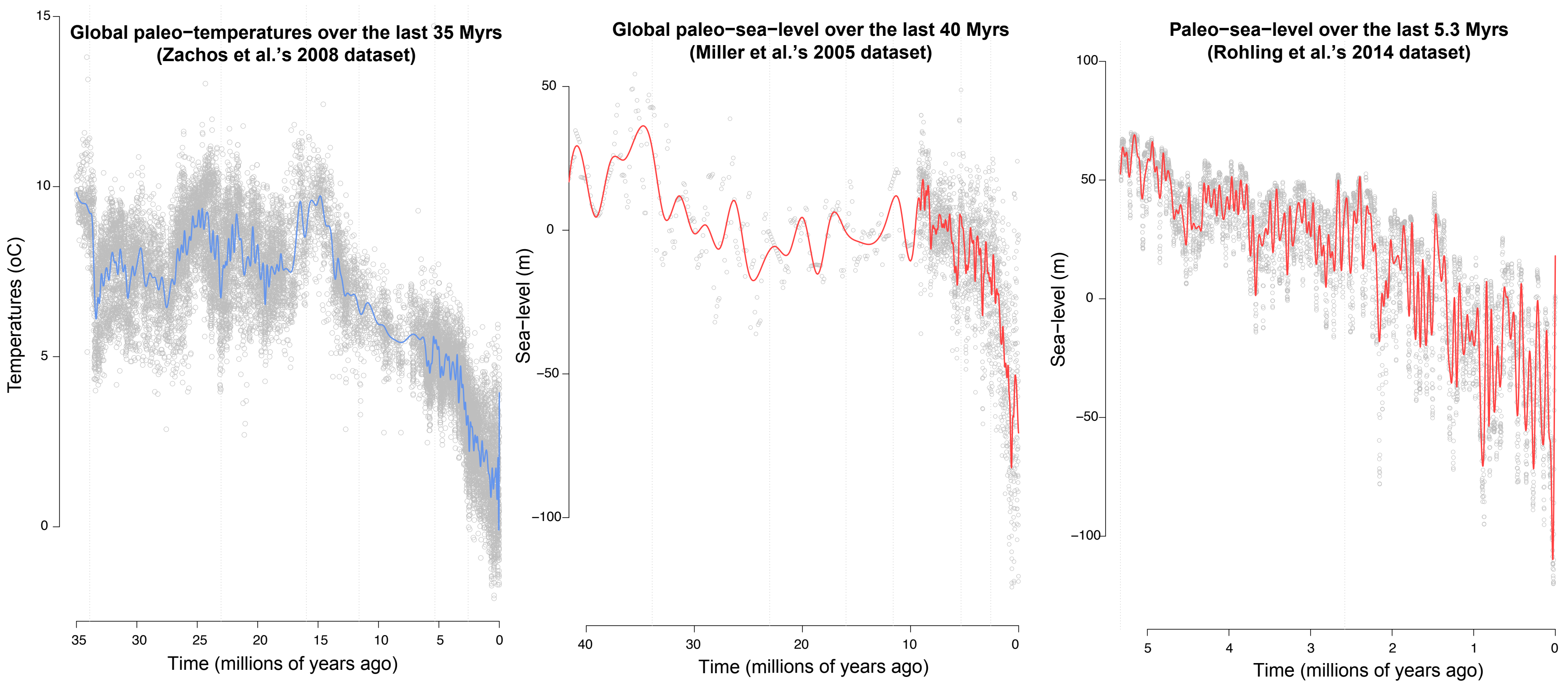

Figure S4 Ancestral range estimates for 'Supermatrix-ITS-like' MCC tree. **(A)** Ancestral ranges that received the highest probability, and **(B)** uncertainty in ancestral range estimates (pies showing all the inferred ranges and their relative probabilities). Colored tip labels denote the Macaronesian endemics. For details on the nine major biogeographic areas see Table S3.

(A)

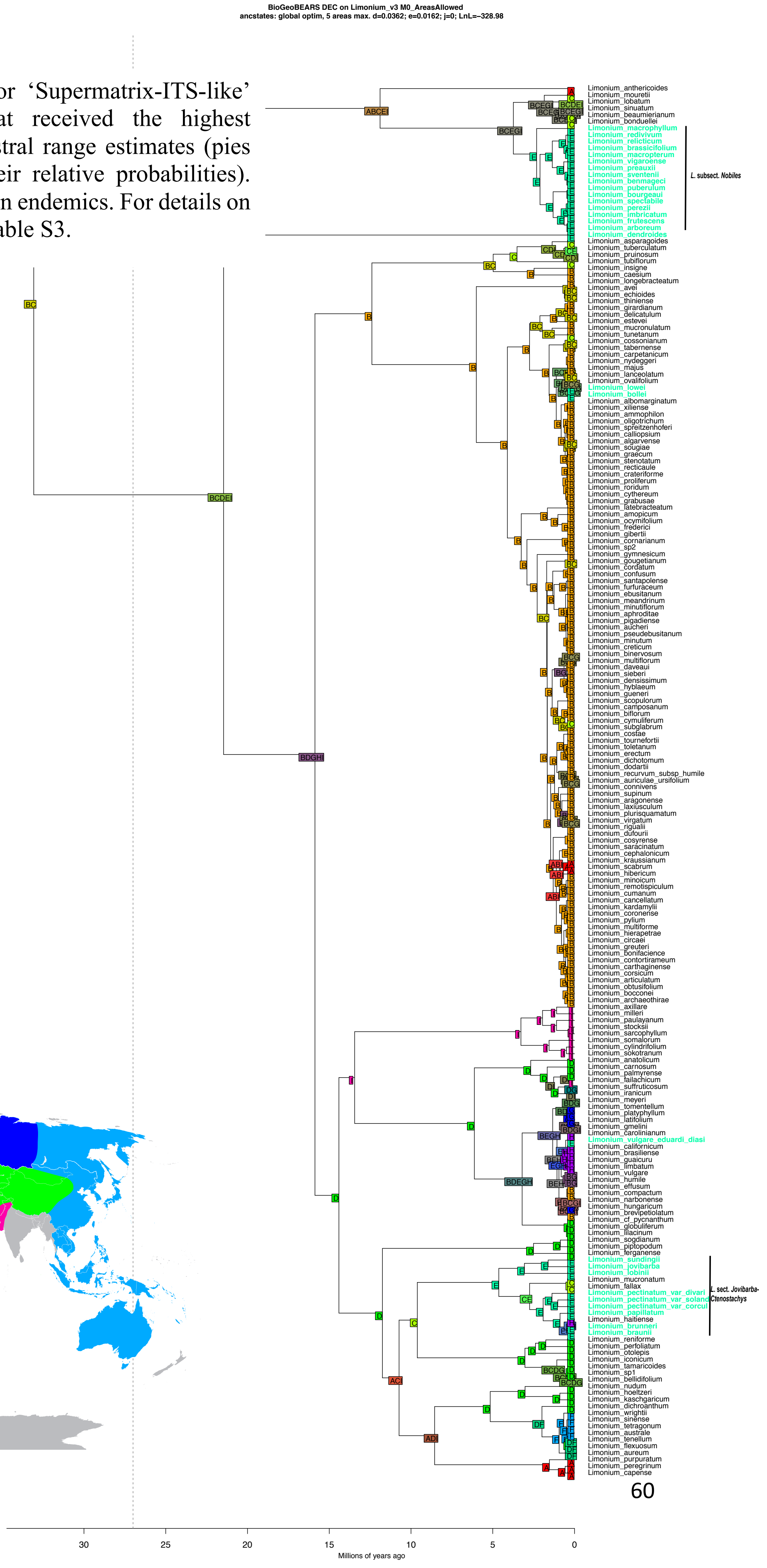

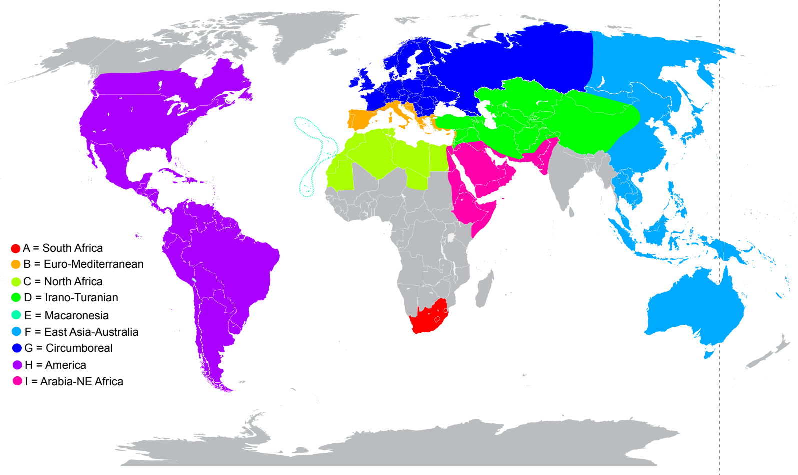

(B)

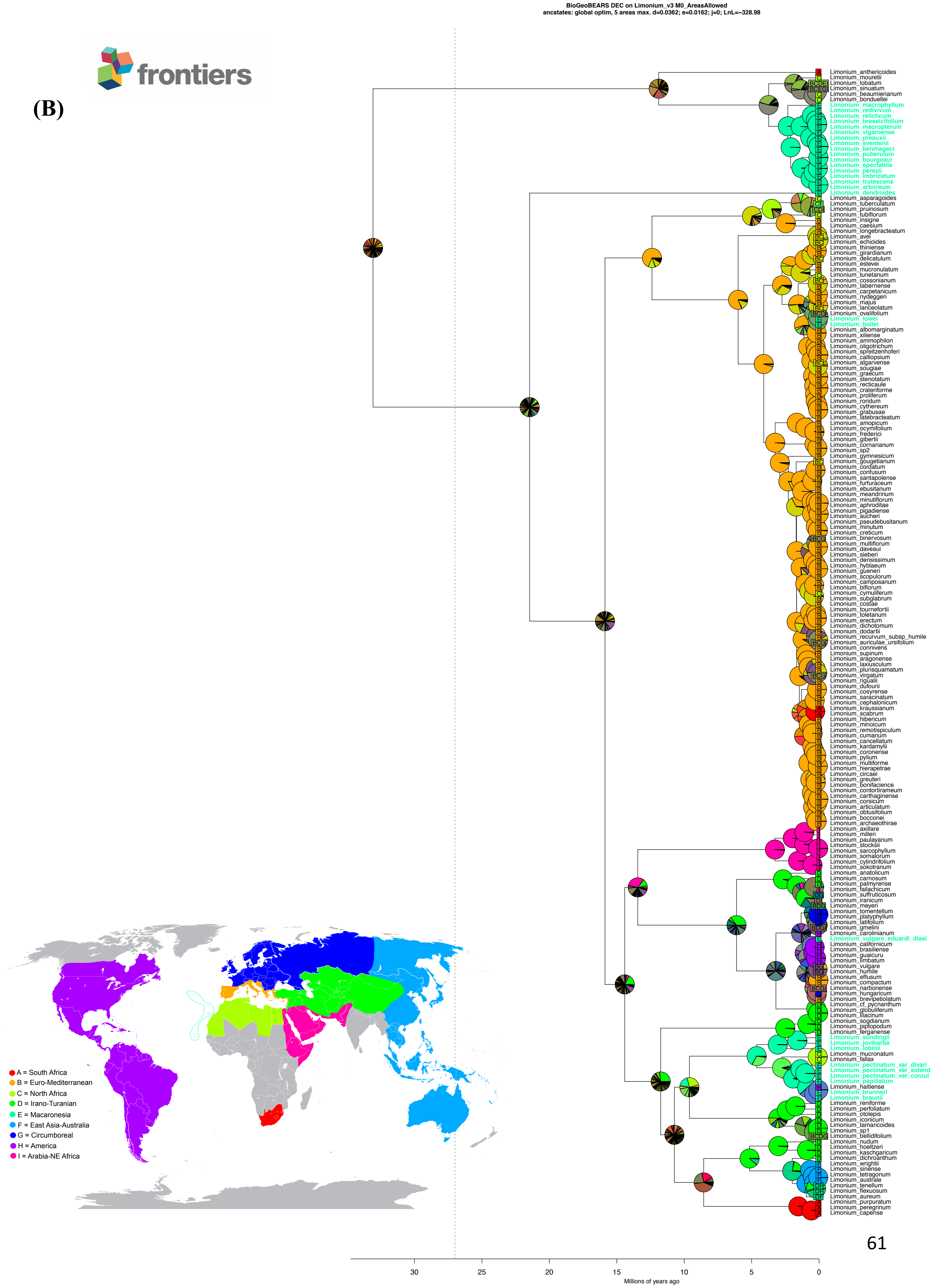
BioGeoBEARS DEC on Limonium_v3 M0_AreasAllowed
ancstates: global optim, 5 areas max. d=0.0362; e=0.0162; j=0; LnL=-328.98
A = South Africa
B = Euro-Mediterranean
C = North Africa
D = Irano-Turanian
E = Macaronesia
F = East Asia-Australia
G = Circumboreal
H = America
I = Arabia-NE Africa
30
25
20
15
10
5
0
Millions of years ago

Figure S5 Ancestral range estimates for 'Supermatrix-cpDNA-like' MCC tree. **(A)** Ancestral ranges that received the highest probability, and **(B)** uncertainty in ancestral range estimates (pies showing all the inferred ranges and their relative probabilities). Colored tip labels denote the Macaronesian endemics. For details on the nine major biogeographic areas see Table S3.

(A)

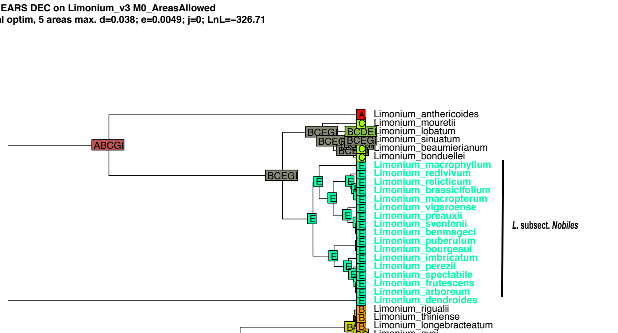
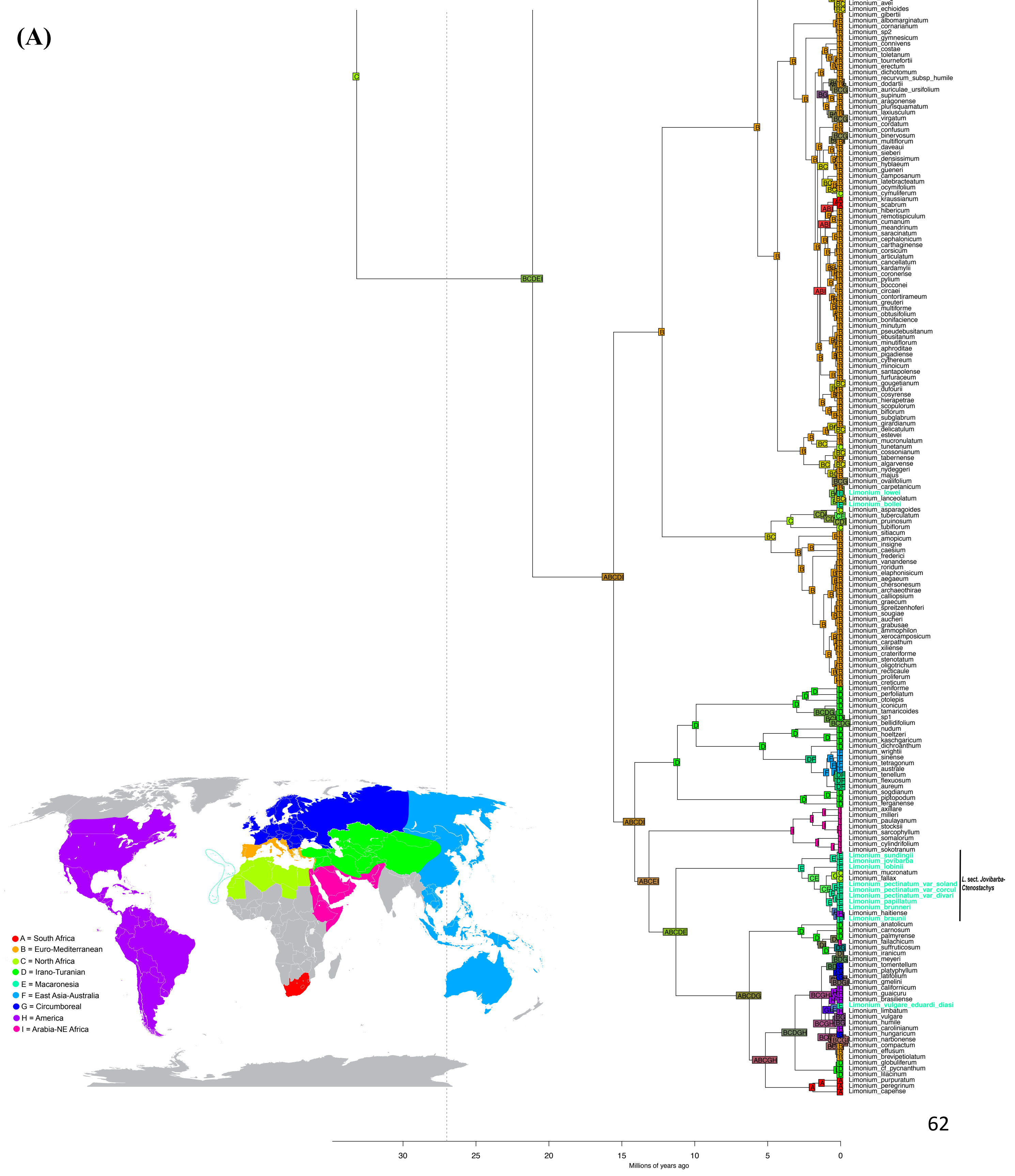

(B)

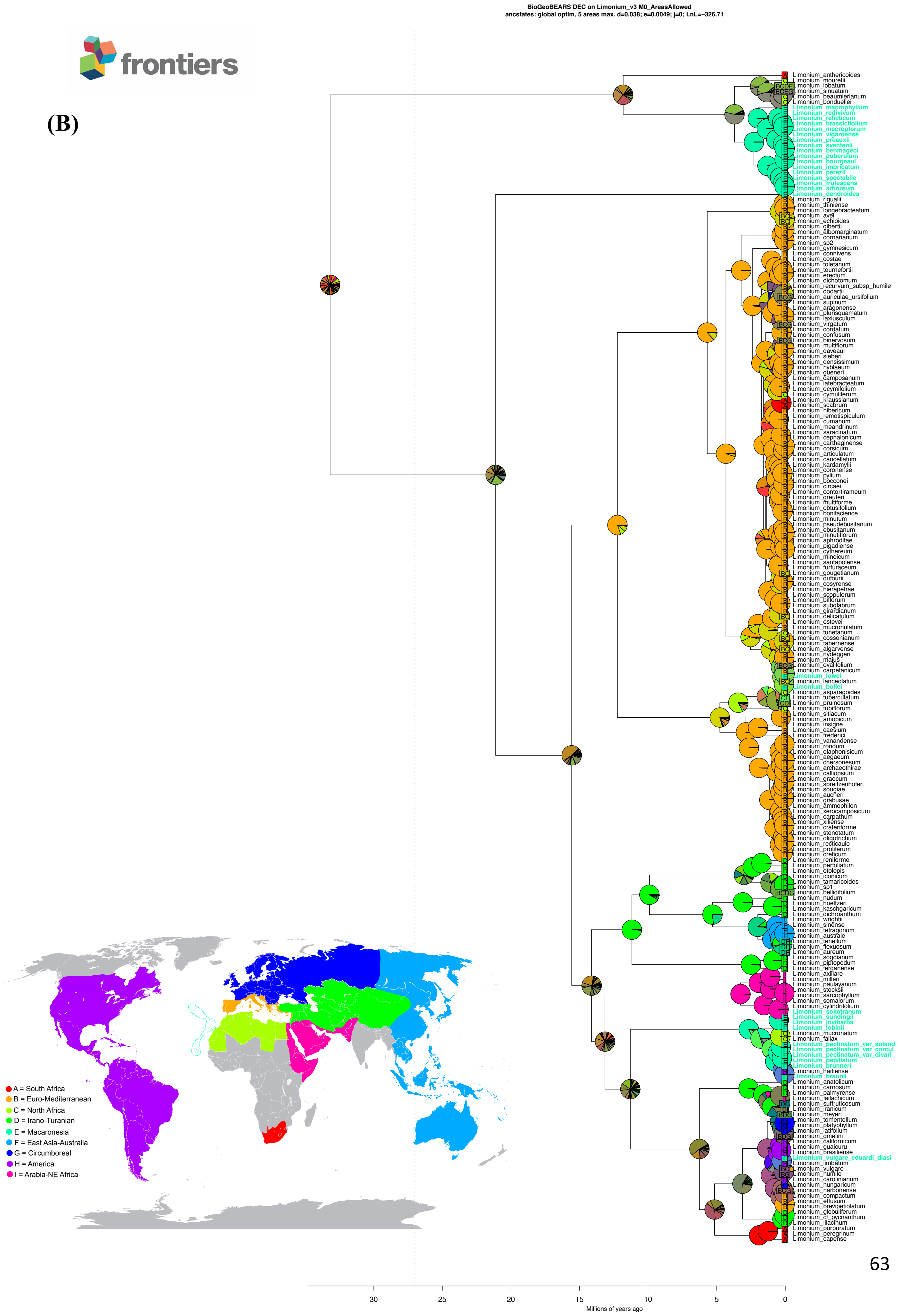

BioGeoBEARS DEC on Limonium_v3 M0_AreasAllowed
ancstates: global optim, 5 areas max. d=0.038; e=0.0049; j=0; LnL=-326.71
Limonium_anthericoides
Limonium_mouretii
Limonium_lobatum
Limonium_sinuatum
Limonium_beaumierianum
Limonium_bonduellei
Limonium_macrophyllum
Limonium_redivivum
Limonium_relicticum
Limonium_brassicifolium
Limonium_macropterum
Limonium_vigaroense
Limonium_preauxii
Limonium_sventenii
Limonium_benmageci
Limonium_puberulum
Limonium_bourgeaui
Limonium_imbricatum
Limonium_perezii
Limonium_spectabile
Limonium_frutescens
Limonium_arboreum
Limonium_dendroides
Limonium_rigualii
Limonium_thiniense
Limonium_longebracteatum
Limonium_avei
Limonium_echioides
Limonium_gibertii
Limonium_albomarginatum
Limonium_cornarianum
Limonium_sp2
Limonium_gymnesicum
Limonium_connivens
Limonium_costae
Limonium_toletanum
Limonium_tournefortii
Limonium_erectum
Limonium_dichotomum
Limonium_recurvum_subsp_humile
Limonium_dodartii
Limonium_auriculae_ursifolium
Limonium_supinum
Limonium_aragonense
Limonium_plurisquamatum
Limonium_laxiusculum
Limonium_virgatum
Limonium_cordatum
Limonium_confusum
Limonium_binervosum
Limonium_multiflorum
Limonium_daveaui
Limonium_sieberi
Limonium_densissimum
Limonium_hyblaeum
Limonium_gueneri
Limonium_camposanum
Limonium_latebracteatum
Limonium_ocymifolium
Limonium_cymuliferum
Limonium_kraussianum
Limonium_scabrum
Limonium_hibericum
Limonium_remotispiculum
Limonium_cumanum
Limonium_meandrinum
Limonium_saracinatum
Limonium_cephalonicum
Limonium_carthaginense
Limonium_corsicum
Limonium_articulatum
Limonium_cancellatum
Limonium_kardamylii
Limonium_coronense
Limonium_pylium
Limonium_bocconei
Limonium_circaei
Limonium_contortirameum
Limonium_greuteri
Limonium_multiforme
Limonium_obtusifolium
Limonium_bonifacience
Limonium_minutum
Limonium_pseudebusitanum
Limonium_ebusitanum
Limonium_minutiflorum
Limonium_aphroditae
Limonium_pigadiense
Limonium_cythereum
Limonium_minoicum
Limonium_santapolense
Limonium_furfuraceum
Limonium_gougetianum
Limonium_dufourii
Limonium_cosyrense
Limonium_hierapetrae
Limonium_scopulorum
Limonium_biflorum
Limonium_subglabrum
Limonium_girardianum
Limonium_delicatulum
Limonium_estevei
Limonium_mucronulatum
Limonium_tunetanum
Limonium_cossonianum
Limonium_tabernense
Limonium_algarvense
Limonium_nydeggeri
Limonium_majus
Limonium_ovalifolium
Limonium_carpetanicum
Limonium_lowei
Limonium_lanceolatum
Limonium_bollei
Limonium_asparagoides
Limonium_tuberculatum
Limonium_pruinosum
Limonium_tubiflorum
Limonium_sitiacum
Limonium_amopicum
Limonium_insigne
Limonium_caesium
Limonium_frederici
Limonium_vanandense
Limonium_roridum
Limonium_elaphonisicum
Limonium_aegaeum
Limonium_chersonesum
Limonium_archaeothirae
Limonium_calliopsium
Limonium_graecum
Limonium_spreitzenhoferi
Limonium_sougiae
Limonium_aucheri
Limonium_grabusae
Limonium_ammophilon
Limonium_xerocamposicum
Limonium_carpathum
Limonium_xiliense
Limonium_crateriforme
Limonium_stenotatum
Limonium_oligotrichum
Limonium_recticaule
Limonium_proliferum
Limonium_creticum
Limonium_reniforme
Limonium_perfoliatum
Limonium_otolepis
Limonium_iconicum
Limonium_tamaricoides
Limonium_sp1
Limonium_bellidifolium
Limonium_nudum
Limonium_hoeltzeri
Limonium_kaschgaricum
Limonium_dichroanthum
Limonium_wrightii
Limonium_sinense
Limonium_tetragonum
Limonium_australe
Limonium_tenellum
Limonium_flexuosum
Limonium_aureum
Limonium_sogdianum
Limonium_piptopodum
Limonium_ferganense
Limonium_axillare
Limonium_milleri
Limonium_paulayanum
Limonium_stocksii
Limonium_sarcophyllum
Limonium_somalorum
Limonium_cylindrifolium
Limonium_sokotranum
Limonium_sundingii
Limonium_jovibarba
Limonium_lobinii
Limonium_mucronatum
Limonium_fallax
Limonium_pectinatum_var_soland
Limonium_pectinatum_var_corcul
Limonium_pectinatum_var_divari
Limonium_papillatum
Limonium_brunneri
Limonium_haitiense
Limonium_braunii
Limonium_anatolicum
Limonium_carnosum
Limonium_palmyrense
Limonium_failachicum
Limonium_suffruticosum
Limonium_iranicum
Limonium_meyeri
Limonium_tomentellum
Limonium_platyphyllum
Limonium_latifolium
Limonium_gmelini
Limonium_californicum
Limonium_guaicuru
Limonium_brasiliense
Limonium_vulgare_eduardi_diasi
Limonium_limbatum
Limonium_vulgare
Limonium_humile
Limonium_carolinianum
Limonium_hungaricum
Limonium_narbonense
Limonium_compactum
Limonium_effusum
Limonium_brevipetiolatum
Limonium_globuliferum
Limonium_cf_pycnanthum
Limonium_lilacinum
Limonium_purpuratum
Limonium_peregrinum
Limonium_capense
A = South Africa
B = Euro-Mediterranean
C = North Africa
D = Irano-Turanian
E = Macaronesia
F = East Asia-Australia
G = Circumboreal
H = America
I = Arabia-NE Africa
30
25
20
15
10
5
0
Millions of years ago

Figure S6 Bar plots of prior and posterior distributions of the number of shifts from BAMM analyses for **(A)** the 'Supermatrix-ITS-like' MCC tree and **(B)** the 'Supermatrix-cpDNA-like' MCC tree. Prior and posterior distributions are denoted by blue and red color, respectively. An intermediate color (grey-red) indicates the range of probability for which prior and posterior distributions overlap. The null model (i.e. constant rates = zero shifts) was never sampled during simulations of the posterior in any of the analyses, showing a strong pattern of rate heterogeneity in our datasets, and significant shifts of posterior from prior distributions.

(A) **(B)**

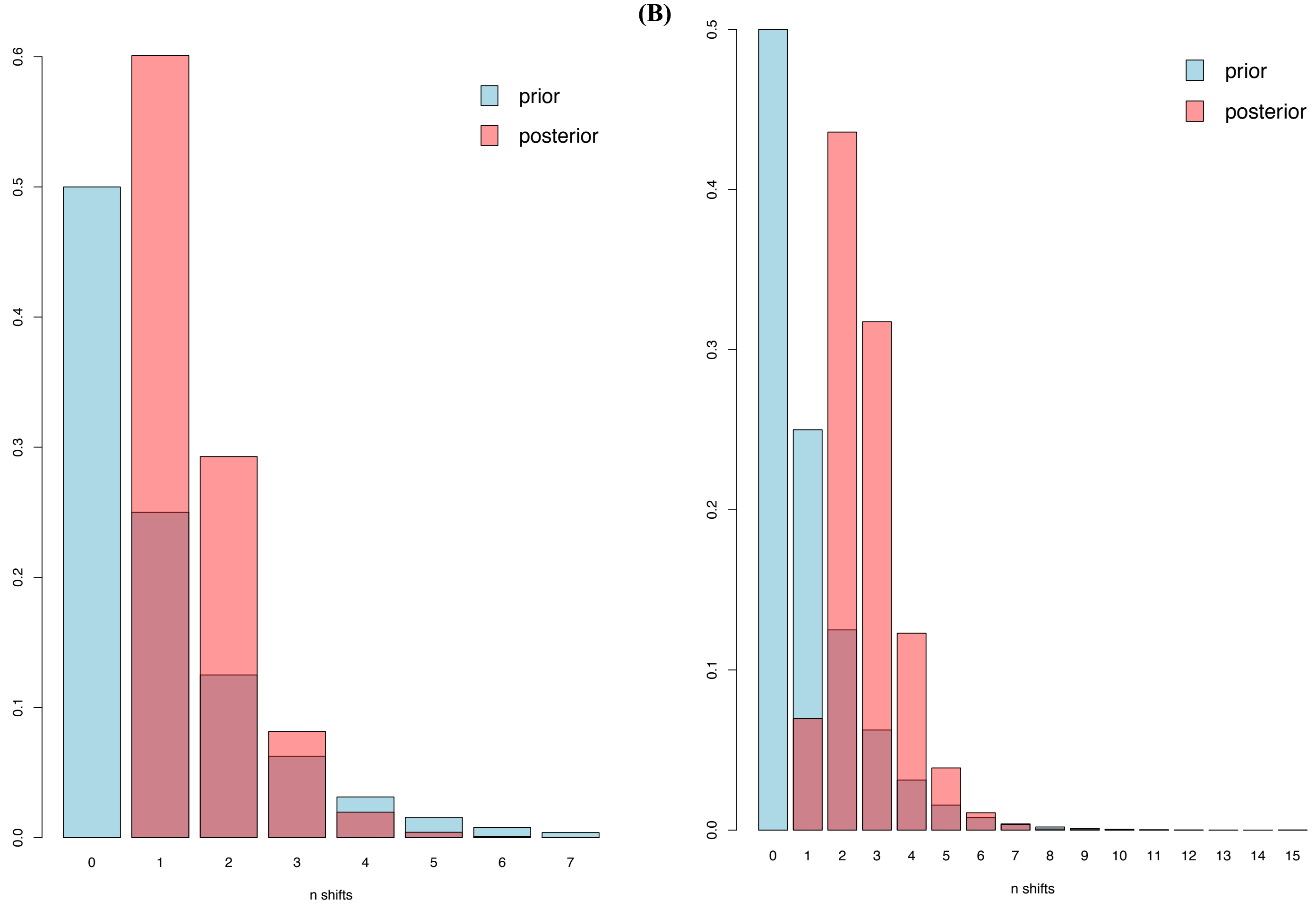

Figure S7 The 95% credible set of shift configurations from BAMM analyses of **(A)** the 'Supermatrix-ITS-like' MCC tree and **(B)** the 'Supermatrix-cpDNA-like' MCC tree.

(A)

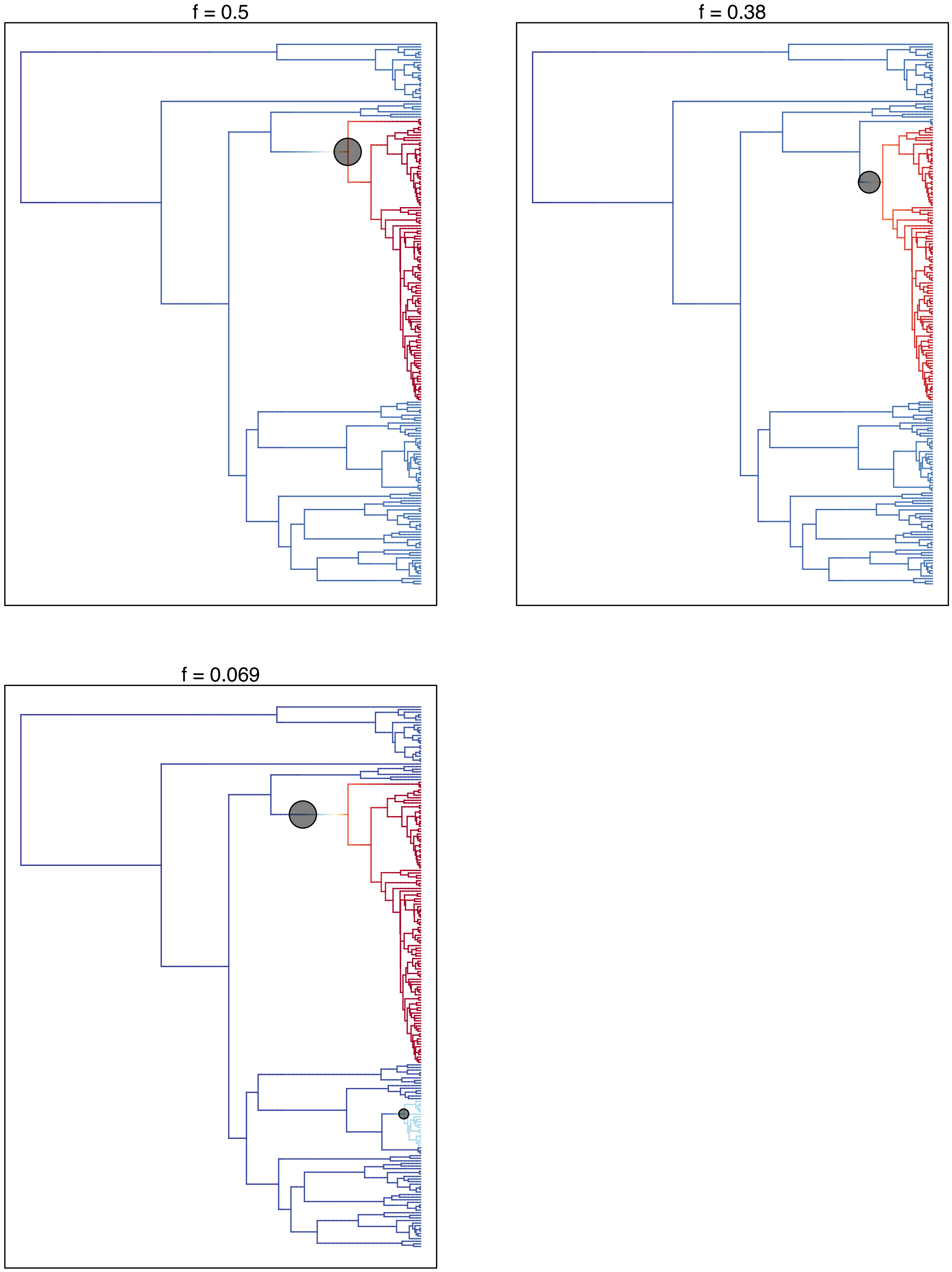

(B)

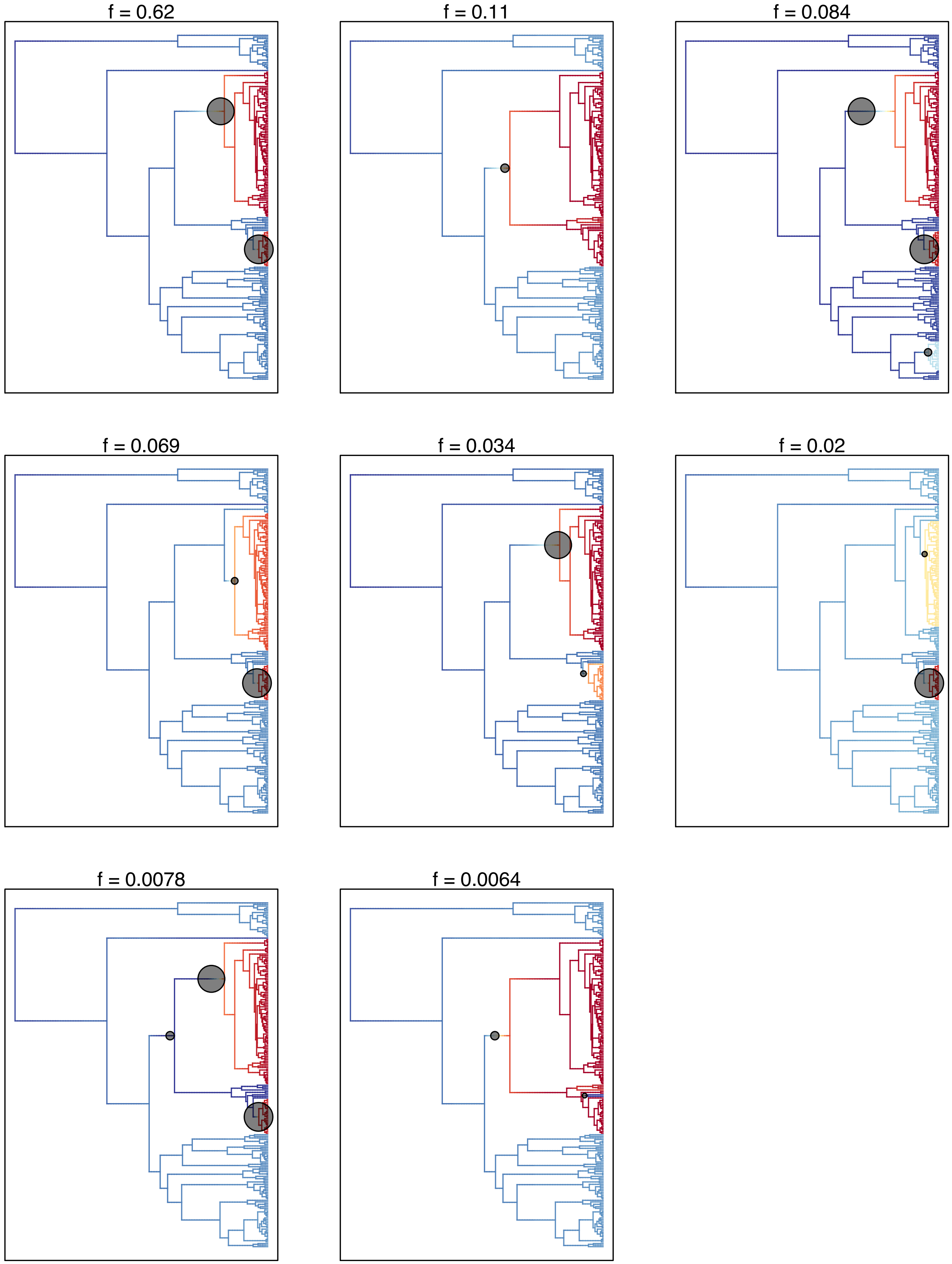

Figure S8 Results on episodic shifts of speciation and extinction rates, and mass extinction events from TESS analyses of **(A)** 'Supermatrix-ITS-like' MCC tree with a model assuming one rate shift, **(B)** 'Supermatrix-ITS-like' MCC tree with a model assuming two rate shifts, **(C)** 'Supermatrix-cpDNA-like' MCC tree with a model assuming one rate shift, and **(D)** 'Supermatrix-cpDNA-like' MCC tree with a model assuming two rate shifts.

(A)

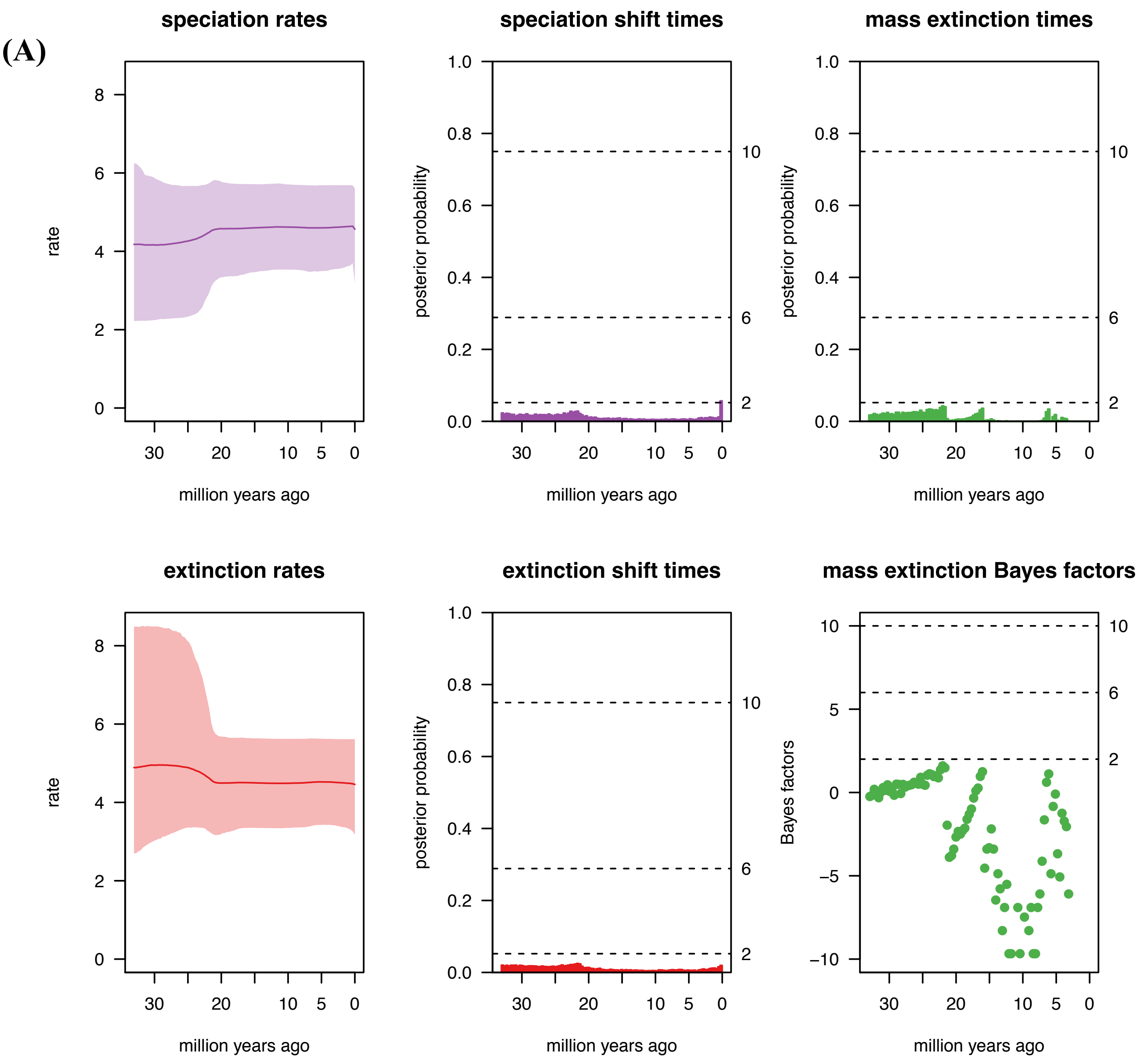

(B)

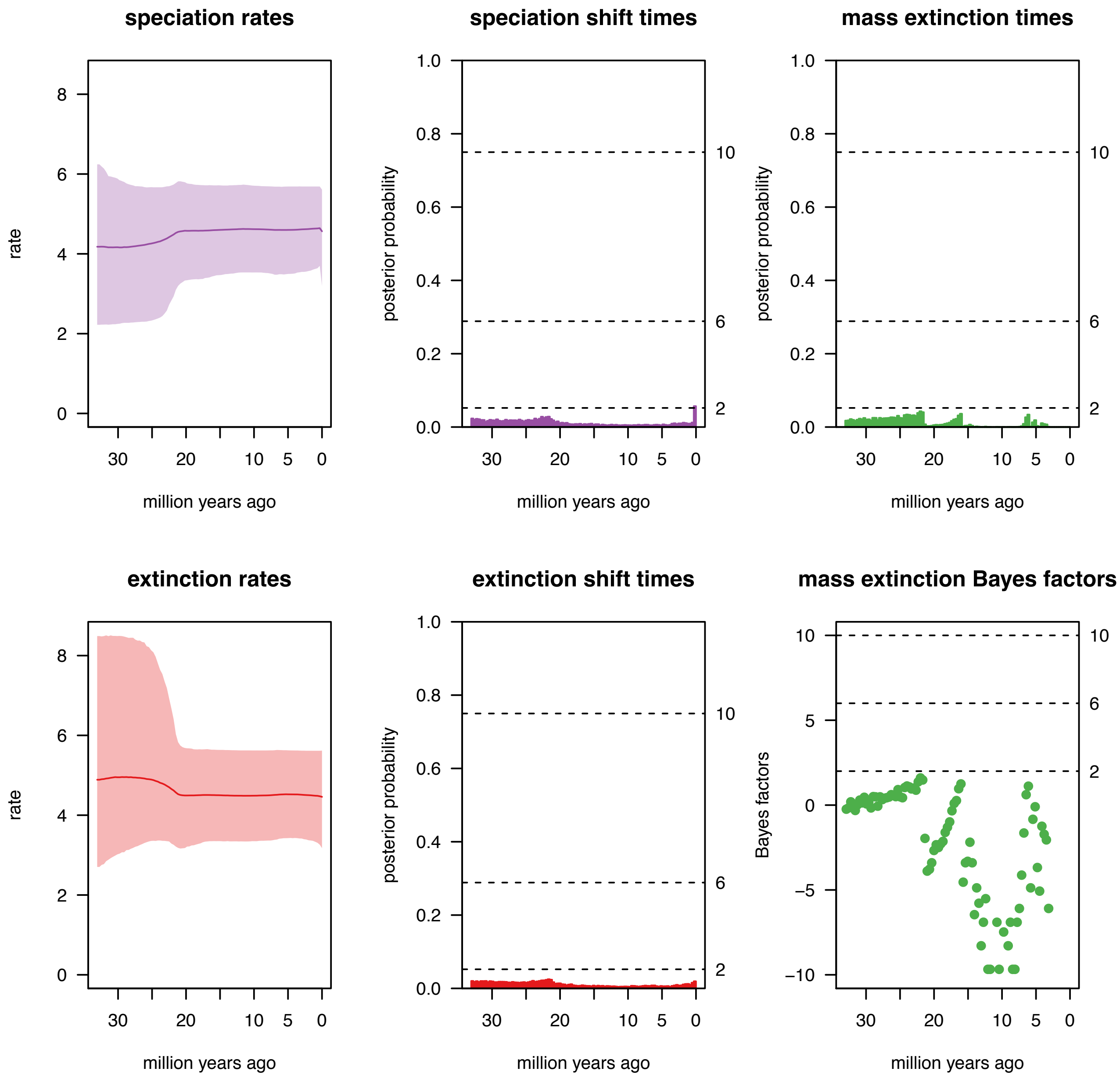
speciation rates
rate
million years ago
speciation shift times
posterior probability
million years ago
mass extinction times
posterior probability
million years ago
extinction rates
rate
million years ago
extinction shift times
posterior probability
million years ago
mass extinction Bayes factors
Bayes factors
million years ago

(C)

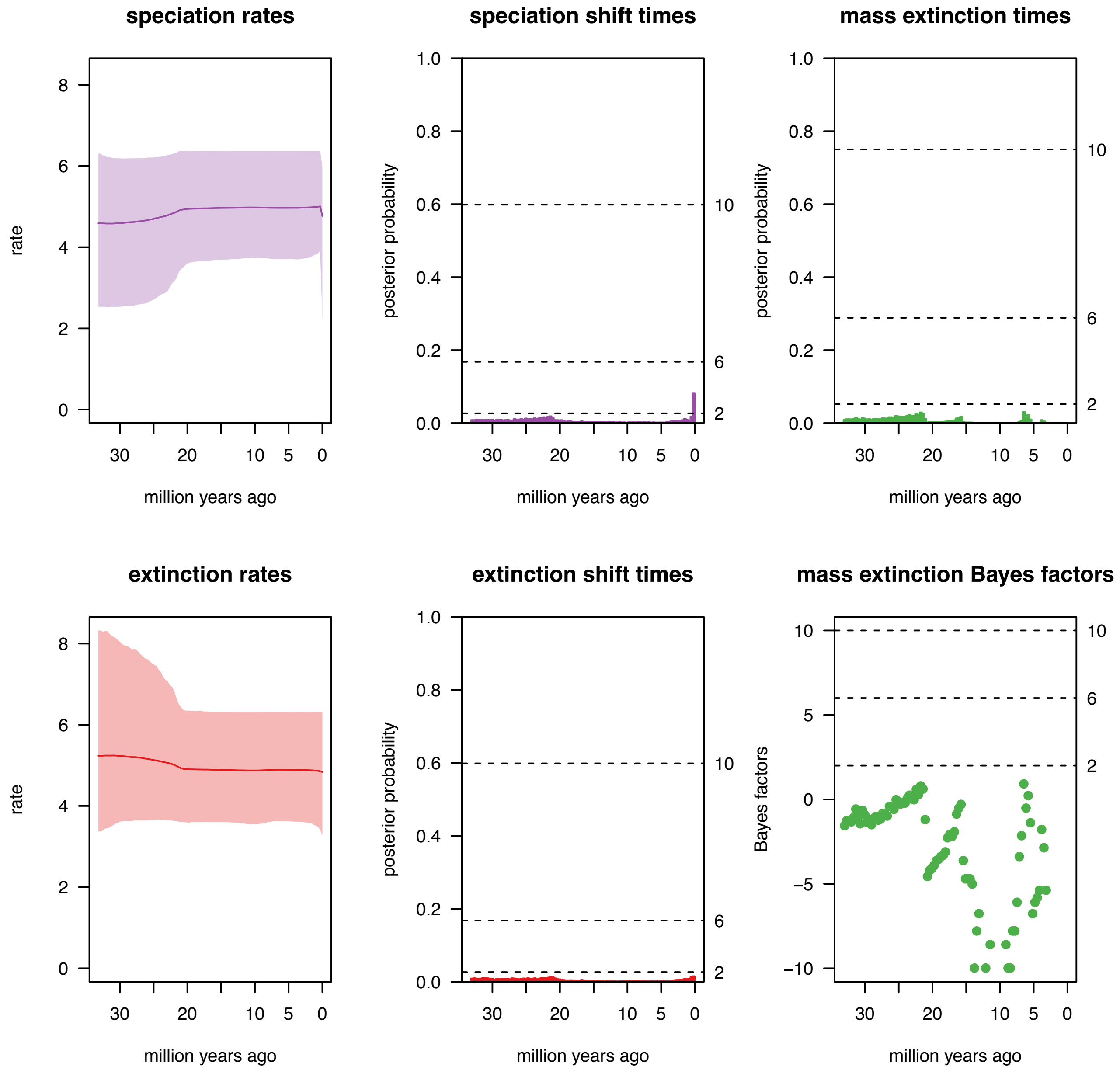

speciation rates
rate
8
6
4
2
0
30
20
10
5
0
million years ago
speciation shift times
posterior probability
1.0
0.8
0.6
0.4
0.2
0.0
10
6
2
million years ago
mass extinction times
posterior probability
million years ago
extinction rates
rate
million years ago
extinction shift times
posterior probability
million years ago
mass extinction Bayes factors
Bayes factors
10
5
0
−5
−10
million years ago

(D)

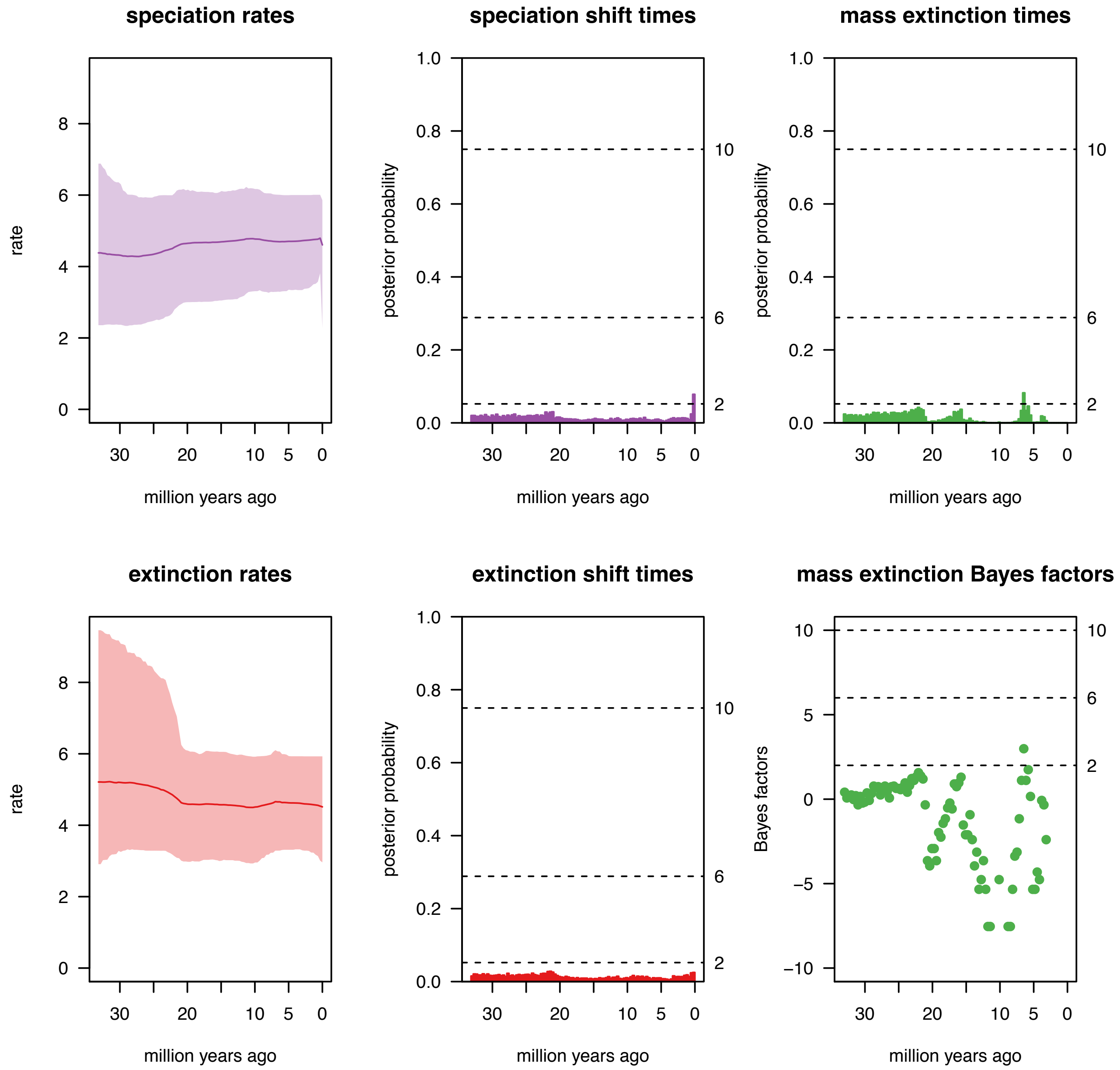
speciation rates
speciation shift times
mass extinction times
extinction rates
extinction shift times
mass extinction Bayes factors
rate
posterior probability
Bayes factors
million years ago
30
20
10
5
0
0.0
0.2
0.4
0.6
0.8
1.0
2
4
6
8
10
−5
−10

Supplementary References

Akhani, H., Malekmohammadi, M., Mahdavi, P., Gharibiyan, A., and Chase, M. W. (2013). Phylogenetics of the Irano-Turanian taxa of *Limonium* (Plumbaginaceae) based on ITS nrDNA sequences and leaf anatomy provides evidence for species delimitation and relationships of lineages. *Bot. J. Linn. Soc.,* 171, 519-550. doi: 10.1111/boj.12015

Arrigoni, P.V. and Diana, S. (1993). Contribution a la connaissance du genre Limonium en Corse. *Candollea,* 48, 631-677.

Arrigoni, P. V., and Diana, S. (1999). Karyology, chorology and bioecology of the genus *Limonium* (Plumbaginaceae) in Sardinia. *Plant Biosyst.*, 133, 63-71. doi: 10.1080/11263509909381533

Artelari, P. (1984). Viosystematiki meleti tou genous Limonium (Plumbaginaceae) stin periohi tou Ioniou pelagous. [Dissertation], [Patras, Greece] Department of Biology, University of Patras.

Artelari, R. (1989). *Limonium creticum* (Plumbaginaceae), a new species from Kriti island (Aegan sea) Greece. *Candollea*, 44, 415-421.

Artelari, R., and Georgiou, O. (1999). Two new species of *Limonium* (Plumbaginaceae) from the island of Kithira (Greece). *Bot. J. Linn. Soc.*, 131, 399-415. doi: 10.1111/j.1095-8339.1999.tb01522.x

Artelari, R., and Georgiou, O. (2002a). The *Limonium ocymifolium* group (Plumbaginaceae) in Greece. –VI International Congress of Systematic and Evolutionary Biology, Patras, Greece, 9-16.

Artelari, R., and Georgiou, O. (2002b). Biosystematic study of the genus Limonium (Plumbaginaceae) in the Aegean area, Greece. III. Limonium on the islands Kithira and Antikithira and the surrounding islets. *Nord. J. Bot.*, 22, 483-502. doi: 10.1111/j.1756-1051.2002.tb01402.x

Artelari, R., and Kamari, G. (1995). *Limonium kardamylii* (Plumbaginaceae), a new species from S Peloponnisos (Greece). *Phyton (Horn)*, 35, 131-137.

Baker, H. G. (1953). Dimorphism and monomorphism in the Plumbaginaceae: II. Pollen and stigmata in the genus Limonium. *Ann. Bot.*, 17, 433-446. doi: 10.1093/oxfordjournals.aob.a083361

Beaulieu, J. M., and O'Meara, B. C. (2016). Detecting hidden diversification shifts in models of trait-dependent speciation and extinction. *Syst. Biol.*, 65, 583-601. doi: 10.1093/sysbio/syw022

Bogdanović, S., Brullo, S., and Mitić, B. (2011). Typification of *Statice cancellata* BERNH. ex BERTOL .(Plumbaginaceae). *Phyton*, 51, 69.

Bogdanović, S., and Brullo, S. (2015). Taxonomic revision of the *Limonium cancellatum* group (Plumbaginaceae) in Croatia. *Phytotaxa*, 215, 1-87. doi: 10.11646/phytotaxa.215.1.1

Borgen, L. (1969). Chromosome numbers of vascular plants from the Canary Islands, with special reference to the occurrence of polyploidy. *Nytt. Mag. Bot,* 16, 81-281.

Borgen, L. (1970) Chromosome numbers of Macaronesian flowering plants. *Nytt. Mag. Bot.,* 17, 145-161.

Brullo, S. and Pavone, P. (1981). Chromosome numbers in the Sicilian species of "Limonium" Miller ("Plumbaginaceae"). *Anales del Jardín Botánico de Madrid*, 37, 535-555.

Brullo, S., Guglielmo, A., and Terrasi M.C. (1990). Osservazioni citotassonomiche su alcune specie di Limonium dell'Italia meridionale. *Giorn. Bot. Ital.,* 124, 75.

Castro, M., and Rossello, J. A. (2007). Karyology of *Limonium* (Plumbaginaceae) species from the Balearic Islands and the western Iberian Peninsula. *Bot. J. Linn. Soc.*, 155, 257-272. doi: 10.1111/j.1095-8339.2007.00703.x

Cowan, R., Ingrouille, M. J., and Lledó, M. D. (1998). The taxonomic treatment of agamosperms in the genus *Limonium* Mill. (Plumbaginaceae). *Folia Geobot.*, 33, 353-366. doi: 10.1007/BF03216212

Crespo, M. B. (2009). A new coastal species of *Limonium* (Plumbaginaceae) from southeastern Spain. *Folia Geobot.*, 44, 177-190. doi: 10.1007/s12224-009-9035-z

Darlington, C. D. and Wylie, A. P., (1956). Chromosome atlas of flowering plants. George Allen and Unwin Ltd., London, UK

Erben, M. (1986). Bemerkungen zur taxonomie der Gattung Limonium III. *Sendtnera- Mitt. Bot. Staatssamml. Münch.,* 22, 203-220.

Erben, M. (1988). Bemerkungen zur Taxonomie der Gattung Limonium IV. *Mitt. Bot. Staatssamml. Münch.*, 27, 381-406.

Erben, M. (1989). Bemerkungen zur Taxonomie der Gattung Limonium V. *Mitt. Bot. Staatssamml. Münch.,* 28, 313-417.

Erben, M. (1991). Bemerkungen zur Taxonomie der Gattung Limonium VI. *Mitt. Bot. Staatssamml. Münch.,* 30, 459-478.

Erben, M. (1993). *Limonium* in *Flora Iberica 3*, ed. Castroviejo, S., Aedo. C., Cirujano, S. et al.. (Madrid: Real Jardín Botánico-C.S.I.C.), 2–143.

Erben, M. (1999). *Limonium nydeggeri*, eine neue Art aus Südwestportugal. *Sendtnera* 6*,* 103–107.

Erben, M. (2001). Bemerkungen zur taxonomie der Gattung Limonium VII. *Sendtnera*, 7, 53-84.

Erben, M. (2006). *Limonium greuteri* (Plumbaginaceae), a new species from the island of Corsica (France). *Willdenowia,* 36, 145-148. doi: doi.org/10.3372/wi.36.36111

Erben, M., and Mucina, L. (2006). *Limonium failachicum* (Plumbaginaceae)—New and so far the only endemic plant from Kuwait. *Folia Geobot.,* 41, 229-235. doi: 10.1007/BF02806481

Febles, R. O. S. A. (1989). Estudios en la flora Macaronésica: algunos números de cromosomas VI. *Botánica Macaronésica*, 17, 57-71.

Fernández-Palacios, J. M., De Nascimento, L., Otto, R., Delgado, J. D., García-del-Rey, E., Arévalo, J. R., et al. (2011). A reconstruction of Palaeo-Macaronesia, with particular reference to the long-term biogeography of the Atlantic island laurel forests. *J. Biogeogr.*, 38, 226-246. doi: 10.1111/j.1365-2699.2010.02427.x

Georgakopoulou, A., Manousou, S., Artelari, R., and Georgiou, O. (2006). Breeding systems and cytology in Greek populations of five *Limonium* species (Plumbaginaceae). *Willdenowia,* 36, 741-750.

Guarino, R., La Rosa, M. and Pignatti S. (2017) *Limonium*. in Flora d'Italia, Vol. 2, 18-48, Edagricole-New Business Media.

Iberite, M., Iamonico, D., and Nicolella, G. (2014). Indagini morfometriche su taxa endemici laziali del genere Limonium (Plumbaginaceae). *Floristica, sistematica ed evoluzione. Palermo: Euroservice Punto Grafica*, 25-26.

Ingrouille, M. J. (1985). The *Limonium auriculae-ursifolium* (POURRET) DRUCE group (Plumbaginaceae) in the Channel Isles. *Watsonia,* 15, 221-229.

Kilian, N., and Leyens, T. (1994). *Limonium lobinii* (Plumbaginaceae), a new species from the Cape Verde Islands, W Africa. *Willdenowia*, 24, 59-63.

Kouzali, I., Artelari, R., and Georgiou, O. (2012). Breeding systems and cytology in Cyprian populations of six *Limonium* species (Plumbaginaceae). *Willdenowia*, 42, 307-313. doi: 10.3372/wi.42.42217

Llorens, L., Gil, L., and Boira, H. (2018). Edaphic characterization of coastal Western Mediterranean *Limonium* (Plumbaginaceae). *Plant Ecol. Evol.*, 151, 175-184. doi: 10.5091/plecevo.2018.1370

Lobin, W., Leyens, T., Kilian, N., Erben, M., and Lewejohann, K. (1995). The genus *Limonium* (Plumbaginaceae) on the Cape Verde Islands, W Africa. *Willdenowia*, 25, 197-214.

Manafzadeh, S., Staedler, Y. M., and Conti, E. (2017). Visions of the past and dreams of the future in the Orient: the Irano-Turanian region from classical botany to evolutionary studies. *Biol. Rev.*, 92, 1365-1388. doi: 10.1111/brv.12287

Meseguer, A. S., Lobo, J. M., Ree, R., Beerling, D. J., and Sanmartín, I. (2015). Integrating fossils, phylogenies, and niche models into biogeography to reveal ancient evolutionary history: the case of *Hypericum* (Hypericaceae). *Syst. Biol.*, 64, 215-232. doi: 10.1093/sysbio/syu088

Özmen, E., Doğan, C., Akaydın, G., and Doğan, M. (2012). Pollen morphology of inland species of Turkish *Limonium* Miller (Plumbaginaceae). *Bio. Di. Con*, 5, 7-18.

Febles, R., and Pérez-Rodríguez, E. Y. (2004). Análisis cariotípico de *Limonium* Mill. Sección *Pteroclados* Boiss. subsección *Nobiles* Boiss. (Plumbaginaceae). *Bot. Macaronésica*, 25, 79-94.

Paradis, G. (2009). Remarques sur la différenciation du genre Limonium (Plumbaginaceae) en Corse. *Bull. Soc. Bot. Centre-Ouest*, 167-186.

Pérez de Paz, J., Febles, R., Acosta, O. F. P., and Corral, M. O. (2017). Flores y polen. Éxito reproductivo de las angiospermas en Canarias. Origen, evolución y conservación. *Rev. Acad. Canar. Cienc,* 29, 111-202.

Ree, R. H., and Sanmartín, I. (2009). Prospects and challenges for parametric models in historical biogeographical inference. *J. Biogeogr.*, 36, 1211-1220. doi: 0.1111/j.1365-2699.2008.02068.x

Rizzotto, M. (1984). A systematic study of the Limonium populations of the Tuscan peninsular coast. *Webbia*, 37, 259-275. doi: 10.1080/00837792.1984.10670279

Rizzotto, M. (2003). Numeri cromosomici per la flora italiana:1442-1445. *Informatore Botanico Italiano,* 35, 95-97.

Róis, A. S., López, C. M. R., Cortinhas, A., Erben, M., Espírito-Santo, D., Wilkinson, M. J., and Caperta, A. D. (2013). Epigenetic rather than genetic factors may explain phenotypic divergence between coastal populations of diploid and tetraploid *Limonium* spp. (Plumbaginaceae) in Portugal. *BMC Plant Biol.,* 13, 205-. doi: 10.1186/1471-2229-13-205

Róis, A. S., Sádio, F., Paulo, O. S., Teixeira, G., Paes, A. P., Espírito-Santo, D., et al. (2016). Phylogeography and modes of reproduction in diploid and tetraploid halophytes of *Limonium* species (Plumbaginaceae): evidence for a pattern of geographical parthenogenesis. *Ann. Bot.*, 117, 37-50. doi: doi.org/10.1093/aob/mcv138

Roskov, Y., Abucay, L., Orrell, T., Nicolson, D., Bailly, N., Kirk, P.M., et al., (2018). Species 2000 & ITIS Catalogue of Life, 30th April 2018. Digital resource at www.catalogueoflife.org/col. Species 2000: Naturalis, Leiden, the Netherlands. ISSN 2405-8858.

Takhtajan, A. (1986). Floristic regions of the world. Berkeley: University of California press.

Sáez, L., and Rosello, J. A. (1999). Is Limonium cavanillesii Erben (Plumbaginaceae) really an extant species?. *Anales del Jardín Botánico de Madrid*, 57, 47-55.

Sanmartín, I., Enghoff, H., and Ronquist, F. (2001). Patterns of animal dispersal, vicariance and diversification in the Holarctic. *Biol. J. Linn. Soc. Lond.*, 73, 345-390. doi: 10.1111/j.1095-8312.2001.tb01368.x

Schäfer, H. (2005). Endemic vascular plants of the Azores: an updated list. *Hoppea*, 66, 275-283.

Zhang, C. Q., and Zheng, X. F. (2016). Karyotype analysis of *Limonium aureum* (L.) Hill. *Journal of Traditional Chinese Veterinary Medicine*, 1, 16.
